# Dysregulation of MicroRNA Regulatory Network in Lower Extremities Arterial Disease

**DOI:** 10.3389/fgene.2019.01200

**Published:** 2019-11-22

**Authors:** Anna Bogucka-Kocka, Daniel P. Zalewski, Karol P. Ruszel, Andrzej Stępniewski, Dariusz Gałkowski, Jacek Bogucki, Łukasz Komsta, Przemysław Kołodziej, Tomasz Zubilewicz, Marcin Feldo, Janusz Kocki

**Affiliations:** ^1^Chair and Department of Biology and Genetics, Medical University of Lublin, Lublin, Poland; ^2^Department of Clinical Genetics, Chair of Medical Genetics, Medical University of Lublin, Lublin, Poland; ^3^Ecotech Complex, Analytical and Programme Centre for Advanced Environmentally-Friendly Technologies, University of Marie Curie-Sklodowska, Lublin, Poland; ^4^Department of Pathology and Laboratory Medicine, Rutgers–Robert Wood Johnson Medical School, New Brunswick, NJ, United States; ^5^Chair and Department of Medicinal Chemistry, Medical University of Lublin, Lublin, Poland; ^6^Department of Vascular Surgery and Angiology, Medical University of Lublin, Lublin, Poland

**Keywords:** miRNA, microRNA, miRNA regulation, miRNA expression, gene expression, low extremities arterial disease, atherosclerosis, biomarker

## Abstract

Atherosclerosis and its comorbidities are the major contributors to the global burden of death worldwide. Lower extremities arterial disease (LEAD) is a common manifestation of atherosclerotic disease of arteries of lower extremities. MicroRNAs belong to epigenetic factors that regulate gene expression and have not yet been extensively studied in LEAD. We aimed to indicate the most promising microRNA and gene expression signatures of LEAD, to identify interactions between microRNA and genes and to describe potential effect of modulated gene expression. High-throughput sequencing was employed to examine microRNAome and transcriptome of peripheral blood mononuclear cells of patients with LEAD, in relation to controls. Statistical significance of microRNAs and genes analysis results was evaluated using DESeq2 and uninformative variable elimination by partial least squares methods. Altered expression of 26 microRNAs (hsa-let-7f-1-3p, hsa-miR-34a-5p, -122-5p, -3591-3p, -34a-3p, -1261, -21-5p, -15a-5p, -548d-5p, -34b-5p, -424-3p, -548aa, -548t-3p, -4423-3p, -196a-5p, -330-3p, -766-3p, -30e-3p, -125b-5p, -1301-3p, -3184-5p, -423-3p, -339-3p, -138-5p, -99a-3p, and -6087) and 14 genes (*AK5*, *CD248*, *CDS2*, *FAM129A*, *FBLN2*, *GGT1*, *NOG*, *NRCAM*, *PDE7A*, *RP11-545E17.3*, *SLC12A2*, *SLC16A10*, *SLC4A10*, and *ZSCAN18*) were the most significantly differentially expressed in LEAD group compared to controls. Discriminative value of revealed microRNAs and genes were confirmed by receiver operating characteristic analysis. Dysregulations of 26 microRNAs and 14 genes were used to propose novel biomarkers of LEAD. Regulatory interactions between biomarker microRNAs and genes were studied *in silico* using R multiMiR package. Functional analysis of genes modulated by proposed biomarker microRNAs was performed using DAVID 6.8 tools and revealed terms closely related to atherosclerosis and, interestingly, the processes involving nervous system. The study provides new insight into microRNA-dependent regulatory mechanisms involved in pathology of LEAD. Proposed microRNA and gene biomarkers of LEAD may provide new diagnostic and therapeutic opportunities.

## Introduction

Peripheral arterial disease (PAD) is one of the most common manifestation of atherosclerosis, a chronic inflammatory process that promotes formation of atheromatous plaques in blood vessels (Norgren et al., 2007; Hamburg and Creager, 2017). PAD is a complex, multifactorial systemic disease linked to genetics, immunity, and environment (Brevetti et al., 2010; Leeper et al., 2012; Fowkes et al., 2017) with severe comorbidities clinically manifested as a myocardial infarction and ischemic stroke (Sigvant et al., 2017). One of the presentations of PAD is lower extremities arterial disease (LEAD), characterized by chronic degenerative changes due to vascular flow deficit caused by stenosis or occlusion of lower limb vessels (Aboyans et al., 2018).

In the last decade, there has been an increasing focus on importance of microRNA (miRNA) diagnostics in diverse diseases (Rupaimoole and Slack, 2017). MiRNA are approximately 22 nucleotides long small RNA molecules, constituting a part of non-coding RNA pool. MiRNA have established role in modulating gene expression (Kim et al., 2016), exhibiting pleiotropic effects, and acting like a switch and a fine-tuner (Mukherji et al., 2011; Lu et al., 2018). MiRNA regulatory networks are considered as an important element in the pathogenesis of atherosclerosis (Zho et al., 2012; Lu et al., 2018; Vogiatzi et al., 2018) with biomarker and therapeutic potential (Hamburg and Leeper, 2015).

Numerous studies established relationships between atherosclerosis-related diseases and alterations in miRNA expression in humans (Fichtlscherer et al., 2010; Bronze-da-Rocha, 2014; Chen and Stewart, 2016; Dolz et al., 2017). Dysregulated expression of miRNAs may serve as a marker of arterial stenosis progression (Jiang et al., 2014; Dolz et al., 2017), plaque stability (Cipollone et al., 2011; Ren et al., 2013; Leistner et al., 2016), and risks of acute ischemic stroke (Li et al., 2015) and cardiovascular death (Karakas et al., 2017). MiRNA expression in various blood components was also correlated with a presence of pro-atherosclerotic risk factors, including elevated lipids levels (Dong et al., 2017), type 2 diabetes mellitus (Al-Kafaji et al., 2010), and high BMI values (Signorelli et al., 2016).

Only a number of studies looked for miRNA signatures in peripheral atherosclerosis, focusing mainly on circulating (plasma, whole blood) miRNA profiling (Li et al., 2011; Stather et al., 2013; Signorelli et al., 2016). MiRNA expression in peripheral blood mononuclear cells (PBMCs) in peripheral atherosclerosis was not extensively studied. PBMCs as an essential element of atherosclerosis-related diseases, carry abundant information about cardiovascular pathophysiology. Differentially expressed miRNAs in PBMCs were already presented as biomarkers of coronary artery disease (CAD) (Hoekstra et al., 2010; Dong et al., 2017).

Selection and monitoring patients with high cardiovascular risk still poses a significant clinical challenge. Despite numerous studies, there is still need for more translational research to understand how the disease is developing in humans. More profound knowledge of pathology, particularly the interactions between molecular and cellular mechanisms, as well as discovery of sensitive and specific biomarkers, are essential to develop optimal diagnostic and treatment approaches.

We applied Next Generation Sequencing (NGS) to investigate miRNA and gene expression profiles in PBMCs from patients with LEAD and healthy controls. The goal was to identify most promising miRNA signatures and genes involved in LEAD which may become novel biomarkers, providing new perspectives on diagnostic and therapeutic opportunities in LEAD.

## Materials and Methods

### Study Population Characteristics

**Figure 1:**
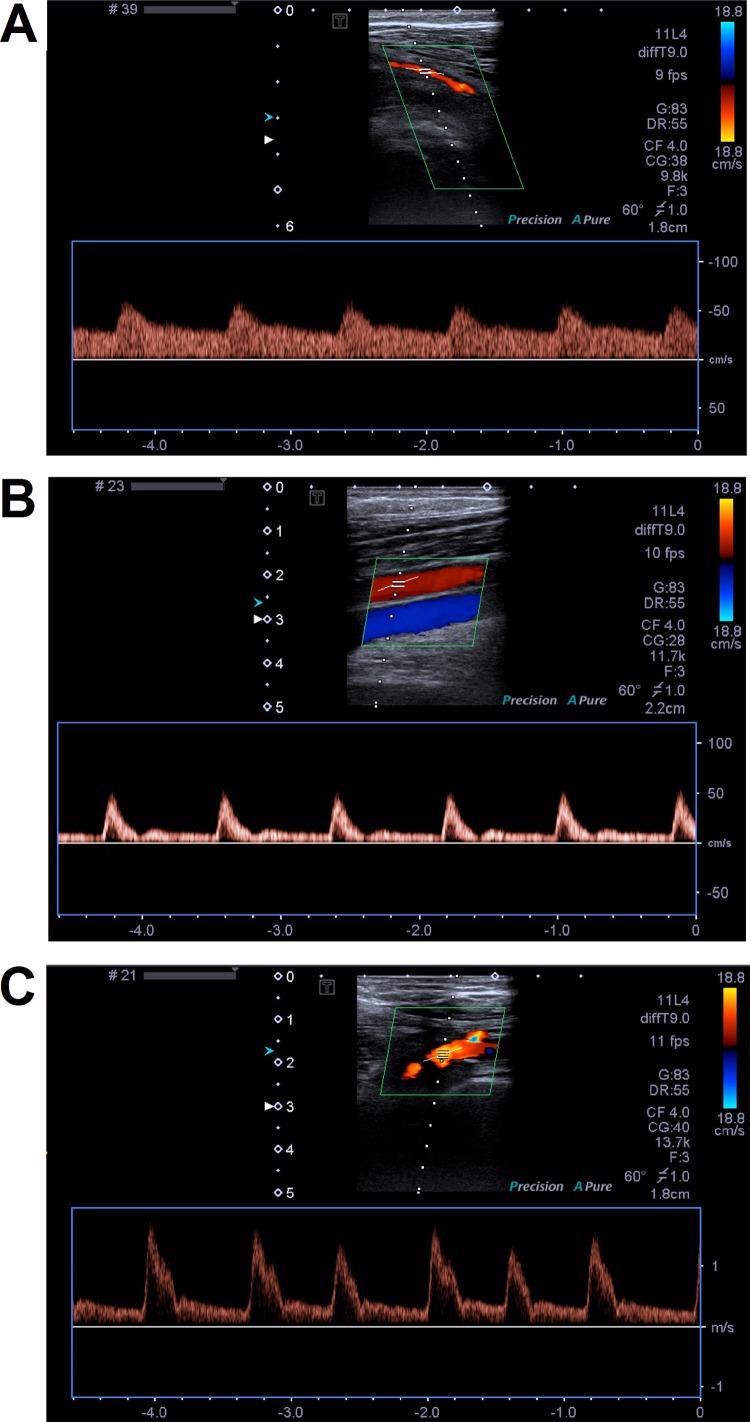
Representative color Doppler images of femoral arteries. Panels **(A)** and **(B)** present femoral artery narrow stenosis caused by atheromatic plaque without calcification. Arterial flow has monophasic waveform with low systolic peaks and continuous diastolic flow. On panel **(B)**, popliteal artery blood flow restored femoral artery flow by inflow from collaterals. On panel **(C)**, femoral artery occlusion and monophasic waveform of flow with high systolic peaks and continuous diastolic flow were observed.

The research was conducted in accordance with the Declaration of Helsinki and approved by Ethics Committee at Medical University of Lublin (decision No. KE-0254/341/2015). Study inclusion occurred between February 2016 and May 2017 involving 40 patients diagnosed with LEAD in Independent Public Clinical Hospital No. 1 in Lublin and 19 non-LEAD volunteers. Informed consent was obtained from all subjects. Characteristics of studied individuals are presented in **Table 1**. Evaluation was performed by vascular surgeon and based on established inclusion criteria. Physical examination consisted of evaluation of peripheral pulses, ankle-brachial index test, treadmill test, angiography, and color flow duplex ultrasound scanning (**Figure 1**). Individuals with LEAD had symptoms of claudication without critical ischemia or tissue loss (Rutherford category 2 or 3). Atherosclerotic lesions were localized in femoral, iliac, or popliteal arteries and were diagnosed with Trans-Atlantic Inter-Society Consensus score B or C. Only patients with chronic complaints originating from LEAD of more than 6 month duration were included. Exclusion criteria were: type 1 diabetes mellitus and previous surgery or percutaneous transluminal angioplasty/stent placement of superficial femoral or iliac arteries. Additional evaluation criteria included smoking habits, medical history, risk factors, pre-existing diagnoses, and medical treatment (**Table 1**).

**Table 1:** Characteristics of 40 patients with LEAD and 19 controls approved to the study.

Characteristic	LEAD population (n = 40)	Control population (n = 19)	*P*
Age	57.58 ± 9.82^*^ 43–71^†^	36.58 ± 9.97^*^ 24–55^†^	1.312E-07
Body Mass Index	27.17 ± 2.621^*^ 21.94–31.64^†^	23.12 ± 3.93^*^ 19.33–32.6^†^	1.729E-04
Smoking	22 (55%)	0 (0%)	1.482E-04
Gender: Male	35 (87.5%)	9 (47%)	2.809E-03
Gender: Female	5 (12.5%)	10 (53%)	
**Indication for intervention**			
Rutherford category 2	34 (85%)	NA	
Rutherford category 3	6 (15%)	NA	
Initial claudication distance (m)	153.63 ± 33.01^*^ 90–200^†^	NA	
Ankle-brachial index	0.683 ± 0.049^*^ 0.59–0.8^†^	NA	
Length of occlusion (cm)	11.25 ± 5.11^*^ 3–25^†^	NA	
**Plaque localization**			
Iliac artery	7 (17.5%)	NA	
Femoral artery	25 (62.5%)	NA	
Popliteal artery	5 (12.5%)	NA	
Iliac and femoral artery	1 (2.5%)	NA	
Femoral and popliteal artery	2 (5%)	NA	
**Risk factors and cardiovascular comorbidities**			
Coronary disease	11 (27.5%)	NA	
Myocardial infarction	8 (20%)	NA	
Diabetes type 2	5 (12.5%)	NA	
Stroke/Transient ischemic attack	2 (5%)	NA	
Hypertension	36 (90%)	NA	
Hypercholesterolemia	31 (77.5%)	NA	
**Medication**			
Statins	34 (85%)	NA	
Acetylsalicylic acid	40 (100%)	NA	
Clopidogrel	8 (20%)	NA	
Beta-adrenergic blockers	27 (67.5%)	NA	
Angiotensin-converting enzyme inhibitor	20 (50%)	NA	
Ca^2+^ channel blockers	11 (27.5%)	NA	
Fibrates	5 (12.5%)	NA	
Metformin	2 (5%)	NA	

*Mean ± SD, ^†^range.

Statistical significance (P) of differences between groups in age and BMI were determined using two-sided Mann Whitney U test. Statistical significance (P) of differences in sex and smoking habits were determined using Chi-Square test. Missing data were addressed to “NA.”

The control (non-LEAD) group contained 19 volunteers. Neither atherosclerotic plaques nor abnormalities in blood flow were observed in iliac, femoral, and popliteal arteries of control individuals during the examination by color flow duplex ultrasound scanning. Only subjects without vascular diseases and comorbidities, including coronary artery disease, myocardial infarction, stroke, diabetes type 2 and without any medication in the medical history were affirmed to the control group. Application of these criteria allows us to select healthy volunteers, however, statistically significant differences in age, BMI, smoking habits, and sex distribution have emerged between LEAD and control groups (**Table 1**).

### Study Material Preparation

Isolation of PBMCs from whole blood samples was conducted by density gradient centrifugation using Gradisol L reagent (Aqua-Med, Poland) (see **Supplementary Material**).

Isolation of small RNA fractions from all PBMCs samples was performed using MirVana microRNA Isolation Kit (Ambion, Lithuania) according to the manufacturer’s protocol. The assessment of quantity and quality of isolated small RNA samples was performed using Agilent 2100 Bioanalyzer (Agilent Small RNA Kit, Agilent Technologies, Lithuania). Software implemented to Agilent 2100 Bioanalyzer was Agilent 2100 Expert Software version B.02.08.SI648.

Total RNA was isolated from PBMCs using TRI Reagent Solution (Applied Biosystems, USA), according to manufacturer’s protocol. The quantity and quality assessment of isolated total RNA was performed using Agilent 2100 Bioanalyzer (Agilent RNA 6000 Pico Kit, Agilent Technologies, USA). The RNA samples with RNA Integrity Number higher than 7 were approved for further experiments.

### miRNA Sequencing

Small RNA libraries were constructed using Ion Total RNA-Seq Kit v2 and barcoded with Ion Xpress RNA-Seq Barcode 01-16 Kit (both Life Technologies, Lithuania). Purifying and size-selecting steps were carried out with Magnetic Bead Cleanup Module kit (Life Technologies, Lithuania). All procedures were performed according to the manufacturer’s protocol “Ion Total RNA-Seq Kit v2” revision B.0. Yield and size distribution of prepared small RNA libraries were assessed with the Agilent 2100 Bioanalyzer instrument and the Agilent High Sensitivity DNA Kit (Agilent Technologies, Lithuania). Barcoded small RNA libraries were diluted to 100 pM concentration with nuclease-free water and pooled (four libraries per chip). Pooled libraries were amplified, prepared for sequencing, and loaded on Ion 540 Chips (Life Technologies, Taiwan) by Ion Chef System (Thermo Fisher Scientific, Singapore). Efficiency of amplification was evaluated using Ion Sphere Quality Control Kit (Life Technologies, USA).

Sequencing was performed using Ion S5 XL System (Thermo Fisher Scientific, USA) and raw data was processed by Torrent Suite Software v5.0.4 (Thermo Fisher Scientific, USA). Raw sequences were aligned to 2,792 human miRNAs from miRBase v21 (http://www.mirbase.org ) using Ion Torrent Small RNA Plugin v5.0.5r3 (Thermo Fisher Scientific, USA) with default settings. For detailed description of the plugin please refer to **Supplementary Material**.

### Transcriptome Sequencing

Due to technical limitations, transcriptome libraries were prepared from 15 total RNA samples isolated from randomly selected representative PBMCs samples (eight from LEAD patients and seven from controls). In order to increase the percentage of coding mRNA, total RNA samples were subjected to ribodepletion procedure using RiboMinus Eukaryote System v2 (Ambion, USA), according to manufacturer’s protocol. Efficiency of rRNA depletion process was verified using Agilent 2100 Bioanalyzer with Agilent RNA 6000 Pico Kit. rRNA-depleted RNA samples were subsequently subjected to transcriptome libraries preparation procedure using the components supplied with Ion Total RNA-Seq Kit v2, Ion Xpress RNA-Seq Barcode 01-16 Kit, and Magnetic Bead Cleanup Module Kit. The procedure was carried out according to manufacturer’s manual “Ion Total RNA-Seq Kit v2” revision B.0. Yield and size distribution of prepared transcriptome libraries were assessed on the Agilent 2100 Bioanalyzer instrument with the Agilent DNA 1000 Kit (Agilent Technologies, Lithuania). Barcoded transcriptome libraries were equalized to 60 pM concentration by dilution in nuclease-free water and multiplexed two samples per chip. Libraries preparation and loading on Ion 540 chips were performed by Ion Chef System. ISP enrichment quality control was carried out with Ion Sphere Quality Control Kit.

Sequencing of transcriptome libraries was performed using Ion S5 XL System and raw data processed by Torrent Suite Software v5.0.4. Raw sequences were aligned to 55,765 genes of hg19 human genome using Ion Torrent RNASeqAnalysis plugin v.5.0.3.0 (Thermo Fisher Scientific, USA).

### Statistical Analysis

LEAD and control groups were evaluated due to differences in age and BMI using two-sided Mann Whitney U test (wilcox.test function in R) and in sex and smoking using Chi-Square test (chisq.test function in R).

Statistical analysis of miRNA and gene sequencing data (resulted from small RNA and transcriptome libraries sequencing, respectively) was performed on biological replicates with R environment (version 3.5.2) and suitable packages.

Control plots of sequencing data, including MA plot, histogram of *P* value frequency and boxplot of counts statistics, were performed using DESeq2 package (Love et al., 2014). Volcano plot for differentially expressed miRNAs, heatmaps with Euclidean clustering, Principal Component Analysis (PCA) plots were performed using R basic functions and packages: data.table 1.11.8, DESeq2 1.18.1, dplyr 0.7.8, ggplot2 3.1.0, ggrepel 0.8.0, gridExtra 2.3, pheatmap 1.0.10, and scatterplot3d 0.3-41 packages.

Differential expression analysis was performed by DESeq2 package 1.18.1, according to R code described in reference manual. MiRNAs and genes with mean of reads lower than one were filtered out. MiRNAs and genes with *P* value below 0.05, adjusted by Benjamini-Hochberg false discovery rate, were considered as statistically significant.

Further confirmation of the differential potential of miRNAs and genes was carried out with UVE-PLS (uninformative variable elimination by partial least squares) method (Centner et al., 1996) using plsVarSel package 0.9.3 (Mehmood et al., 2012), according to R code described in reference manual. UVE-PLS analysis was applied to filtered read counts data (mean of reads lower than 1) and transformed using regularized log normalization (rlog function in DESeq2 package). In order to find appropriate number of PLS components for UVE-PLS, normalized data were primarily subjected to standard PLS analysis with leave-one-out cross-validation using plsr function in plsVarSel package. Ultimately, UVE-PLS analysis was performed with four PLS components, 1,000 iterations, and default cut-off threshold.

Correlations between miRNA expression and characteristics of studied groups were performed using DESeq2 method for categorical variables (sex, smoking) and two-sided Spearman rank correlation test covered in cor.test R function for continuous variables (age, BMI).

Predicting value of selected miRNAs and genes was assessed using receiver operating characteristic (ROC) analysis, carried out with pROC package version 1.12.1 (Robin et al., 2011) according to reference manual.

Deconvolution of miRNA expression data was performed using UNDO 1.26.0 package (Wang et al., 2015) on data normalized by DESeq function implemented in DESeq2 package. For deconvolution of gene expression data, a “quanTIseq” method (Finotello et al., 2019) implemented in immunedeconv 2.0.0 package (Sturm et al., 2019) was applied to tpm-normalized data using scater 1.12.2 package (McCarthy et al., 2017).

Identification of validated (miRecords, miRTarBase, TarBase databases) and predicted (DIANA-microT, ElMMo, MicroCosm, miRanda, miRDB, PicTar, PITA, TargetScan databases) interactions between selected miRNAs and genes was performed using multiMiR package 1.2.0 (Ru et al., 2014) and reference manual. Obtained interactions were presented in the regulatory network, visualized using Cytoscape v3.5.1 software (Shannon et al., 2003).

Functional analysis for genes contained in the network was performed using DAVID (Database for Annotation, Visualization, and Integrated Discovery) 6.8 database (Huang et al., 2009a; Huang et al., 2009b) using default whole genome background for *Homo sapiens*. For each analyzed gene, associated terms of KEGG (Kyoto Encyclopedia of Genes and Genomes) pathway maps, Reactome database, and GAD (Genetic Association Database) database were harvested. The enrichment analysis of GO (Gene Ontology) terms was carried out separately for up- and downregulated genes.

## Results

### Study Population Analysis

Representative examples of color duplex ultrasound examination of femoral artery occlusions and flow were shown on the **Figure 1**. Characteristics of 40 LEAD patients and 19 non-LEAD controls are presented in **Table 1**. Inclusion of healthy, LEAD-negative confirmed individuals in control group resulted in differences between patients and control groups in sex, age, BMI (body mass index), and smoking with respective *P* values 2.809E-03, 1.312E-07, 1.729E-04, and 1.482E-04 (**Table 1** and **Supplementary Figure 1**).

### Primary Results

Representative electrophoregrams of small RNA samples, total RNA samples, and corresponding libraries were presented in **Supplementary Figures 2** and **3**. Parameters describing small RNA samples, small RNA libraries, and results of sequencing data primary analysis of small RNA libraries were shown in **Supplementary Table 1**. Parameters of transcriptome libraries and results of sequencing data primary analysis of transcriptome libraries were presented in **Supplementary Table 2**. Sequencing data control plots (boxplot of Cook’s distances across samples, MA plot, and histogram of *P* values frequency) for small RNA and transcriptome analysis are presented in **Supplementary Figures 4** and **5**, respectively.

### Differential Expression Analysis of miRNA

MiRNA expression levels were compared between 40 LEAD patients and 19 non-LEAD controls. For differential expression analysis of miRNA, DESeq2, and UVE-PLS methods were applied to investigate expression data of 2,792 miRNAs and common significantly dysregulated miRNAs indicated by both methods were selected.

**Figure 2:**
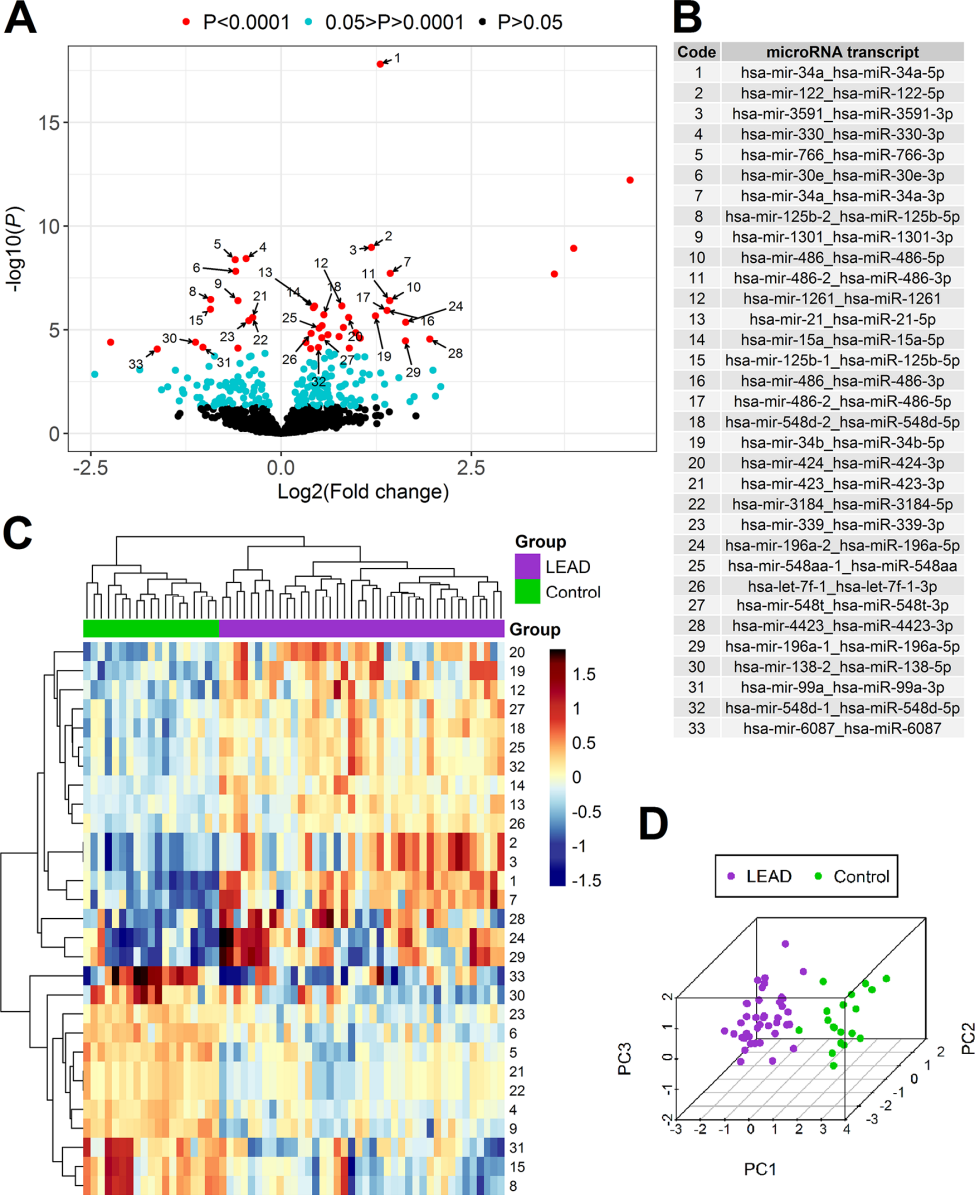
Differential expression analysis of miRNA in PBMCs samples derived from 40 patients with LEAD (LEAD) and 19 non-LEAD controls (Control). Volcano plot **(A)** illustrating the arrangement of negative log10 of *P* values and log2 fold changes for 1,181 differentially expressed miRNA transcripts indicated using DESeq2 method. Thirty-three miRNA transcripts resulted from DESeq2 method with *P* < 0.0001 overlapping with informative miRNAs returned from UVE-PLS analysis were pointed with numbers corresponding to the code and names in table on panel **(B)**. Heatmap with Euclidean clustering **(C)** and 3D PCA plot **(D)**, generated based on expression of selected 29 miRNA transcripts (after excluding four miRNA transcripts belonging to miR-486 family). Numbers of heatmap rows correspond to transcript names according to “Code” column on panel **(B)**.

DESeq2 filtering and comparison analysis of the miRNA expression signatures in PBMCs derived from LEAD patients and non-LEAD controls revealed 1,181 differentially expressed miRNA transcripts in LEAD patients (**Figure 2A**). Two hundred thirty-one miRNA transcripts (134 upregulated and 97 downregulated) were significantly differentially expressed with *P* < 0.05 (**Supplementary Tables 3** and **4**, respectively).

To limit false positive results, a set of 47 differentially expressed miRNA transcripts (for 39 miRNAs) of high significance (*P* < 0.0001) was chosen for further comparison with UVE-PLS results.

To optimally filter miRNAs with uninformative character, the UVE-PLS method was used. Distribution of PLS (Partial Least Squares) components and predictive ability of applied PLS model were presented on **Supplementary Figure 6**. Application of UVE-PLS to filtered and normalized miRNA expression data has returned 86 informative miRNA transcripts, 37 were upregulated and 49 were downregulated (**Supplementary Tables 5** and **6**, respectively).

The comparison of 47 differentially expressed miRNA transcripts identified by DESeq2 method (with *P* < 0.0001) and 86 differentially expressed miRNA transcripts identified by UVE-PLS method disclosed 33 miRNA transcripts (for 28 miRNAs) common for both methods (**Figure 2**).

PCA analysis and heatmap with Euclidean clustering were performed to visualize clustering pattern of samples and 33 selected miRNA transcripts (**Supplementary Figure 7**). Expression of four miRNA transcripts belonging to miR-486 family (hsa-mir-486-2_hsa-miR-486-3p, hsa-mir-486_hsa-miR-486-5p, hsa-mir-486_hsa-miR-486-3p, hsa-mir-486-2_hsa-miR-486-5p) disturbed clear separation of LEAD and control groups. Exclusion of these four miRNA transcripts from PCA analysis and heatmap with Euclidean clustering improved ability to differentiate LEAD and control groups by remaining 29 miRNA transcripts (**Figures 2C, D**).

In order to identify factor(s) affecting expression of four excluded miRNA transcripts, correlations with age, BMI, sex, and smoking habits were evaluated. Statistically significant correlation was found between expression of 4 excluded miRNA transcripts belonging to miR-486 family and age, BMI, and smoking habits (**Supplementary Table 7**). Therefore, expression of these miRNA transcripts was presumably affected by differences in age, BMI, and smoking habits, rather than by presence of LEAD. For this reason, these miRNA transcripts were excluded from further confirmation of predictive capability.

Predicting value of differential expression of remaining 29 miRNA transcripts was evaluated using ROC analysis carried out with pROC package. Areas under ROC curves were above 0.8 for all evaluated miRNA transcripts, indicating good performance of LEAD classification (**Table 2**, **Supplementary Figure 8** and **Supplementary Table 8**). These 29 miRNA transcripts give 26 miRNAs (15 upregulated and 11 downregulated), which constitute a proposed panel of miRNA biomarkers of LEAD (**Table 2**).

**Table 2:** Set of 29 differentially expressed miRNA transcripts with *P* < 0.0001 (from DESeq2 analysis) and with significance confirmed by UVE-PLS in patients with LEAD, in comparison with non-LEAD controls. Indicated 29 miRNA transcripts give 26 miRNAs (miRNA IDs).

No.	miRNA transcript	miRNA ID^*^	*P*	Fold change	PLS coefficient	ROC-AUC
**Upregulated miRNA transcripts**						
1.	hsa-mir-34a_hsa-miR-34a-5p	hsa-miR-34a-5p	1.59E-18	2.4673	4.30E-02	0.9697
2.	hsa-mir-122_hsa-miR-122-5p	hsa-miR-122-5p	1.09E-09	2.2755	3.22E-02	0.9079
3.	hsa-mir-3591_hsa-miR-3591-3p	hsa-miR-3591-3p	1.09E-09	2.2749	3.21E-02	0.9079
4.	hsa-mir-34a_hsa-miR-34a-3p	hsa-miR-34a-3p	1.94E-08	2.6999	3.79E-02	0.9053
5.	hsa-mir-1261_hsa-miR-1261	hsa-miR-1261	7.06E-07	1.7390	1.98E-02	0.8961
6.	hsa-mir-21_hsa-miR-21-5p	hsa-miR-21-5p	7.29E-07	1.3550	7.46E-03	0.9237
7.	hsa-mir-15a_hsa-miR-15a-5p	hsa-miR-15a-5p	8.64E-07	1.3423	1.12E-02	0.9250
8.	hsa-mir-548d-2_hsa-miR-548d-5p	hsa-miR-548d-5p	1.90E-06	1.4763	1.04E-02	0.8724
9.	hsa-mir-34b_hsa-miR-34b-5p	hsa-miR-34b-5p	2.14E-06	2.3585	2.24E-02	0.8776
10.	hsa-mir-424_hsa-miR-424-3p	hsa-miR-424-3p	2.54E-06	1.8492	1.28E-02	0.8329
11.	hsa-mir-196a-2_hsa-miR-196a-5p	hsa-miR-196a-5p	4.36E-06	3.1111	3.91E-02	0.8553
12.	hsa-mir-548aa-1_hsa-miR-548aa	hsa-miR-548aa	8.36E-06	1.4134	6.82E-03	0.8579
13.	hsa-let-7f-1_hsa-let-7f-1-3p	hsa-let-7f-1-3p	1.49E-05	1.3152	8.39E-03	0.8566
14.	hsa-mir-548t_hsa-miR-548t-3p	hsa-miR-548t-3p	2.45E-05	1.4475	7.90E-03	0.8474
15.	hsa-mir-4423_hsa-miR-4423-3p	hsa-miR-4423-3p	2.85E-05	3.8730	3.69E-02	0.8276
16.	hsa-mir-196a-1_hsa-miR-196a-5p	hsa-miR-196a-5p	3.42E-05	3.0991	3.04E-02	0.8132
17.	hsa-mir-548d-1_hsa-miR-548d-5p	hsa-miR-548d-5p	7.20E-05	1.4049	7.06E-03	0.8408
**Downregulated miRNA transcripts**						
1.	hsa-mir-330_hsa-miR-330-3p	hsa-miR-330-3p	3.73E-09	0.7264	−1.32E-02	0.9316
2.	hsa-mir-766_hsa-miR-766-3p	hsa-miR-766-3p	4.26E-09	0.6585	−1.45E-02	0.9579
3.	hsa-mir-30e_hsa-miR-30e-3p	hsa-miR-30e-3p	1.54E-08	0.6616	−1.38E-02	0.9118
4.	hsa-mir-125b-2_hsa-miR-125b-5p	hsa-miR-125b-5p	3.54E-07	0.5270	−2.10E-02	0.9013
5.	hsa-mir-1301_hsa-miR-1301-3p	hsa-miR-1301-3p	3.92E-07	0.6743	−1.62E-02	0.9066
6.	hsa-mir-125b-1_hsa-miR-125b-5p	hsa-miR-125b-5p	1.04E-06	0.5256	−1.69E-02	0.8789
7.	hsa-mir-3184_hsa-miR-3184-5p	hsa-miR-3184-5p	2.59E-06	0.7722	−7.79E-03	0.9026
8.	hsa-mir-423_hsa-miR-423-3p	hsa-miR-423-3p	2.59E-06	0.7722	−7.79E-03	0.9039
9.	hsa-mir-339_hsa-miR-339-3p	hsa-miR-339-3p	3.65E-06	0.7448	−2.01E-02	0.8763
10.	hsa-mir-138-2_hsa-miR-138-5p	hsa-miR-138-5p	4.05E-05	0.4586	−4.17E-02	0.8224
11.	hsa-mir-99a_hsa-miR-99a-3p	hsa-miR-99a-3p	7.04E-05	0.4906	−1.92E-02	0.8079
12.	hsa-mir-6087_hsa-miR-6087	hsa-miR-6087	8.46E-05	0.3240	−2.60E-02	0.8211

*According to miRBase 22 (http://www.mirbase.org/). The table presents P values (FDR with Benjamini-Hochberg correction) and fold changes obtained from DESeq2 analysis, PLS coefficients obtained from UVE-PLS analysis and areas under ROC curves (ROC-AUC) resulted from ROC analysis. MiRNA transcripts were divided into upregulated and downregulated groups and ordered according to increasing P value.

Deconvolution analysis of miRNA expression data using UNDO package provided estimated proportions of two cell subpopulations in LEAD and Control groups. Distributions of these proportions were presented in **Supplementary Figure 9**.

### Differential Expression Analysis of Genes

RNA samples derived from randomly selected eight LEAD patients and seven non-LEAD controls were subjected to transcriptome sequencing. Differential expression analysis of genes was performed using DESeq2 and UVE-PLS methods and common significantly dysregulated genes indicated by both methods were selected.

**Figure 3:**
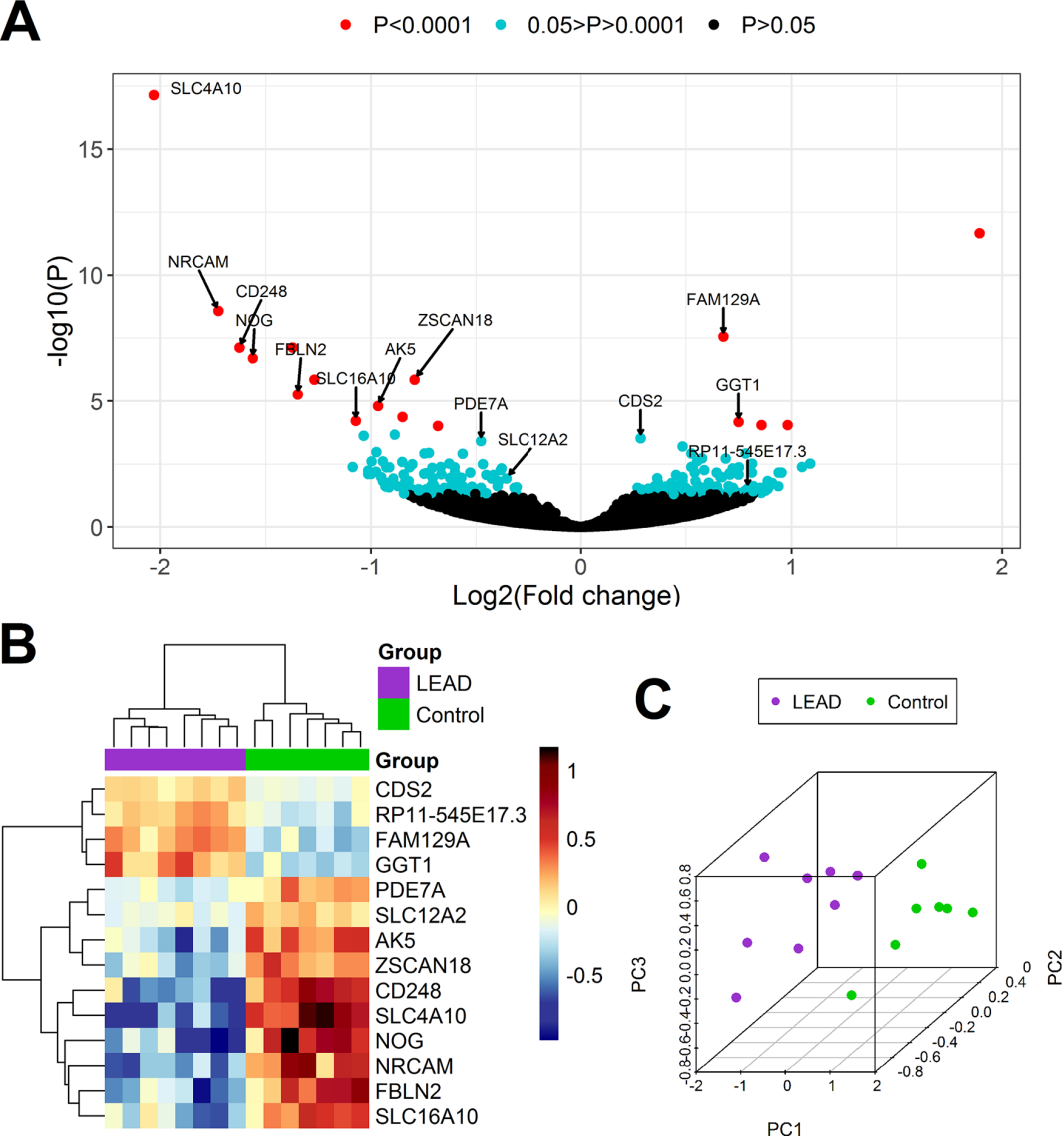
Differential expression analysis of genes in PBMCs samples derived from 8 patients with LEAD (LEAD) and 7 non-LEAD controls (Control). Volcano plot **(A)** illustrating the arrangement of negative log10 of *P* values and log2 fold changes for 17,868 differentially expressed genes obtained from DESeq2 analysis. Heatmap with Euclidean clustering **(B)** and 3D PCA plot **(C)** were generated based on expression of 14 genes determined as indicative for LEAD by both DESeq2 and UVE-PLS methods.

DESeq2 analysis revealed 17,868 differentially expressed genes in LEAD group when compared to non-LEAD controls (**Figure 3A**). Genes resulted with significantly changed expression (*P* < 0.05) formed a set of 221 genes—108 were upregulated and 113 were downregulated (**Supplementary Tables 9** and **10**, respectively).

UVE-PLS analysis indicated 14 genes (4 upregulated and 10 downregulated) with informative value to differentiate LEAD and control groups (*AK5*, *CD248*, *CDS2*, *FAM129A*, *FBLN2*, *GGT1*, *NOG*, *NRCAM*, *PDE7A*, *RP11-545E17.3*, *SLC12A2*, *SLC16A10*, *SLC4A10*, and *ZSCAN18*). Distribution of PLS components and predictive ability of applied PLS model were presented on **Supplementary Figure 10**.

The comparison of the set of 221 differentially expressed genes revealed in DESeq2 method (*P* < 0.05), with the set of 14 differentially expressed genes disclosed in UVE-PLS method, indicated, that all 14 genes selected in UVE-PLS analysis were included in the set of 221 genes obtained from DESeq2 analysis.

Differential expression of these 14 selected genes was confirmed by Euclidean clustering and PCA analysis (**Figures 3B, C**).

ROC analysis showed that expression of those 14 genes has strong predictive value with an area under the ROC curve >0.964 (**Table 3**, **Supplementary Table 11** and **Supplementary Figure 11**).

**Table 3:** Set of 14 differentially expressed genes with *P* < 0.05 (from DESeq2 analysis) and with significance confirmed by UVE-PLS genes in patients with LEAD, in comparison with non-LEAD controls.

Gene symbol	Gene name	*P*	Fold change	PLS coefficient	ROC-AUC
**Upregulated genes**					
*FAM129A*	Family with sequence Similarity 129 member A	2.78E-08	1.5991	2.04E-03	1.000
*GGT1*	Gamma-glutamyltransferase 1	6.78E-05	1.6811	1.94E-03	1.000
*CDS2*	CDP-diacylglycerol synthase 2	3.02E-04	1.2174	8.69E-04	0.982
*RP11-545E17.3*	—	2.99E-02	1.7321	1.24E-03	1.000
**Downregulated genes**					
*SLC4A10*	Solute carrier family 4 member 10	7.09E-18	0.2448	−5.87E-03	1.000
*NRCAM*	Neuronal cell adhesion molecule	2.73E-09	0.3024	−3.71E-03	1.000
*CD248*	CD248 molecule	7.49E-08	0.3241	−4.59E-03	1.000
*NOG*	Noggin	2.03E-07	0.3390	−4.42E-03	1.000
*ZSCAN18*	Zinc finger and SCAN Domain containing 18	1.41E-06	0.5774	−2.33E-03	1.000
*FBLN2*	Fibulin 2	5.54E-06	0.3928	−3.48E-03	1.000
*AK5*	Adenylate kinase 5	1.57E-05	0.5112	−2.69E-03	1.000
*SLC16A10*	Solute carrier family 16 member 10	6.17E-05	0.4752	−2.60E-03	0.982
*PDE7A*	Phosphodiesterase 7A	3.87E-04	0.7184	−1.19E-03	0.964
*SLC12A2*	Solute carrier family 12 member 2	1.17E-02	0.7812	−1.10E-03	1.000

The table presents P values (FDR with Benjamini-Hochberg correction) and fold changes obtained from DESeq2 analysis, PLS coefficients obtained from UVE-PLS analysis and areas under ROC curves (ROC-AUC) resulted from ROC analysis. Genes were divided into upregulated and downregulated groups and ordered according to increasing P value.

Therefore, 14 evaluated genes constitute a proposed panel of transcriptomic biomarkers of LEAD (**Table 3**).

Deconvolution of gene expression data revealed information about 11 immune cell subtypes in the subjects included to transcriptome analysis (**Supplementary Figure 12**). We did not observed significant differences between samples. It suggests that there is no meaningful impact of subpopulation composition in PBMC on study outcome.

### *In Silico* Identification of miRNA: Gene Interactions

**Figure 4:**
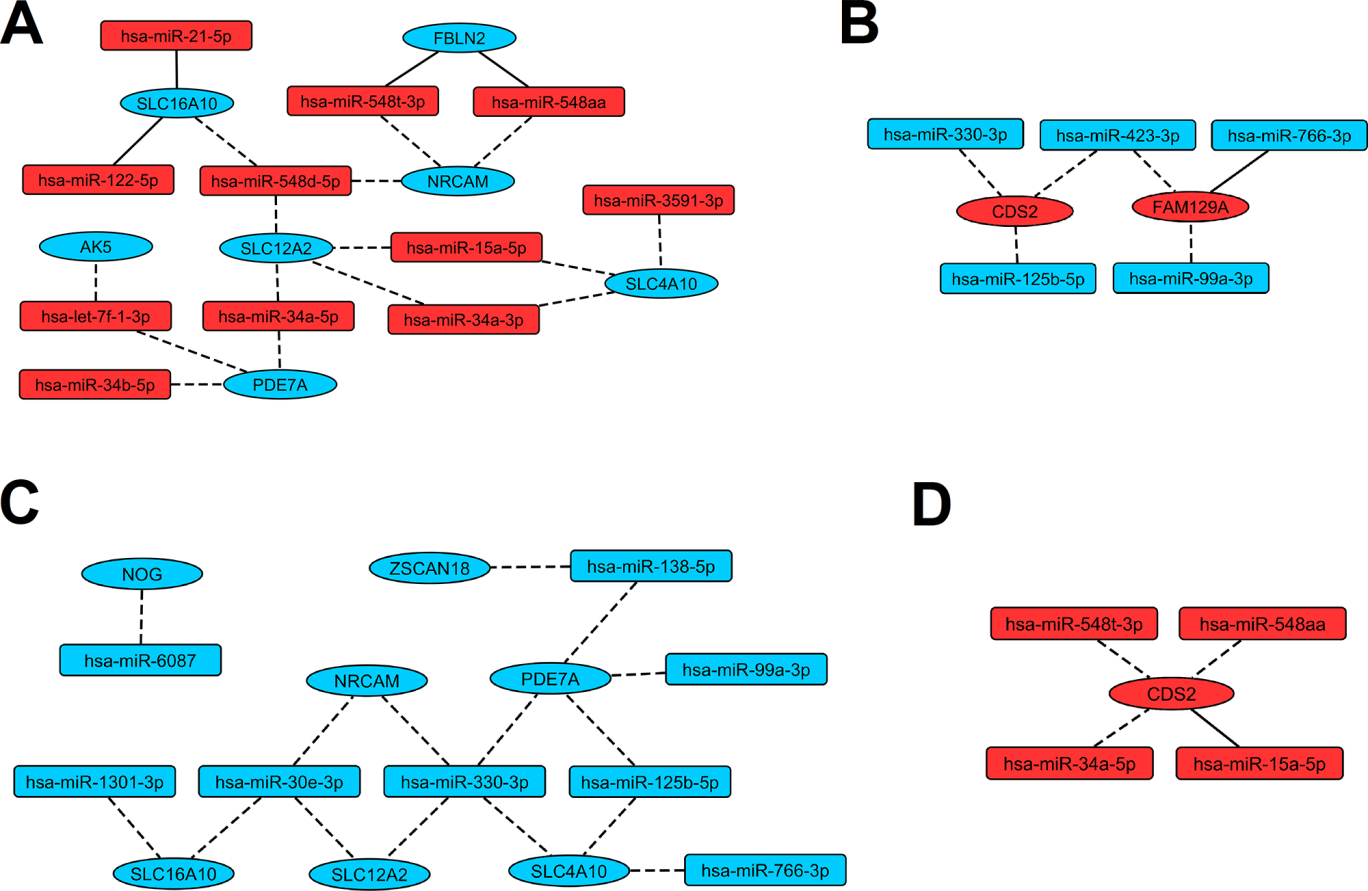
Regulatory networks presenting interactions between miRNAs and genes revealed as indicative for LEAD. Red and blue color of nodes mean respectively upregulated and downregulated miRNAs or genes. Solid and dashed edges indicate validated and predictive interactions, respectively. Panel **(A)** presents interactions between upregulated miRNAs and downregulated genes, panel **(B)** presents interactions between downregulated miRNAs and upregulated genes, panel **(C)** presents interactions between downregulated miRNAs and downregulated genes, panel **(D)** presents interactions between upregulated miRNAs and upregulated genes.

In order to identify miRNA:gene interactions between 26 selected miRNAs and 14 selected genes, both groups were processed by multiMiR package. Analysis returned six validated interactions (**Supplementary Table 12**) and 43 top 10% predicted interactions (**Supplementary Table 13**). Determined interactions formed a regulatory network containing 20 miRNAs and 11 genes, which was constructed using Cytoscape software (**Figure 4**).

### Functional Analysis of miRNA Targets

Functional analysis of 11 target genes (*AK5*, *CDS2*, *FAM129A*, *FBLN2*, *NOG*, *NRCAM*, *PDE7A*, *SLC12A2*, *SLC16A10*, *SLC4A10*, and *ZSCAN18*), present in the regulatory network, was performed using DAVID 6.8 tools and resulted associations are presented in **Table 4**.

**Table 4:** Functional analysis of eleven genes, which dysregulated expression in patients with LEAD was connected to miRNA modulatory function.

**Functional analysis of upregulated genes ** ***(CDS2, FAM129A)***	
*KEGG, Reactome, GAD and GAD Class terms*	
**Gene**	**Terms**
*CDS2*	**KEGG**: Glycerophospholipid metabolism, phosphatidylinositol signaling system, metabolic pathways **Reactome**: Synthesis of PG (phosphatidylglycerol) **GAD**: Type 2 Diabetes|edema|rosiglitazone, tobacco use disorder **GAD class**: Pharmacogenomic, chemdependency
*FAM129A*	**GAD**: Insulin resistance, insulin, celiac disease, echocardiography **GAD class**: Immune, cardiovascular, metabolic
*Gene Ontology terms associated with both CDS2 and FAM129A*	
**Category**	**Terms**
GO biological process	Phosphate-containing compound metabolic process, phosphorus metabolic process, cellular biosynthetic process, organic substance biosynthetic process, biosynthetic process, membrane, cytoplasm, cellular metabolic process, primary metabolic process, organic substance metabolic process, metabolic process
GO cellular component	Membrane, cytoplasm, cellular metabolic process, primary metabolic process, organic substance metabolic process, metabolic process, membrane-bounded organelle, organelle, intracellular part, intracellular, cell part
**Functional analysis of downregulated genes ** ***(AK5, ZSCAN18, NOG, FBLN2, NRCAM, PDE7A, SLC12A2, SLC16A10, SLC4A10)***	
*KEGG, Reactome, GAD and GAD Class terms*	
**Gene**	**Terms**
*FBLN2*	**Reactome**: Molecules associated with elastic fibers **GAD**: Personality, Alzheimer disease, hemoglobins, alcoholism, kidney aging **GAD class**: Chemdependency, psych, neurological, aging, hematological
*NRCAM*	**KEGG**: Cell adhesion molecules (CAMs) **Reactome**: Neurofascin interactions, bicarbonate transporters, NrCAM interactions, interaction between L1 and ankyrins **GAD**: Schizophrenia, tobacco use disorder, autism, mathematics ability, autism obsessive compulsive disorder, several psychiatric disorders **GAD class**: chemdependency, psych, other
*PDE7A*	**KEGG**: Purine metabolism, Morphine addiction
	**Reactome**: G alpha (s) signaling events
	**GAD**: Hemoglobins, HIV-1, tobacco use disorder, type 2 diabetes|edema|rosiglitazone
	**GAD class**: Chemdependency, hematological, infection, pharmacogenomic
*SLC12A2*	**KEGG**: Pancreatic secretion, Vibrio cholera infection, salivary secretion **Reactome**: Cation-coupled chloride cotransporters **GAD**: Schizophrenia, tobacco use disorder, body weights and measures, celiac disease, myocardial infarction, brain imaging in schizophrenia (interaction), carcinoid tumor, hearing loss noise-induced **GAD class**: Chemdependency, psych, other, neurological, metabolic, cancer, cardiovascular, immune
*SLC16A10*	**KEGG**: Protein digestion and absorption, thyroid hormone signaling pathway Reactome: Amino acid transport across the plasma membrane **GAD**: Cholesterol LDL **GAD class**: Metabolic
*SLC4A10*	**Reactome**: Bicarbonate transporters **GAD**: Tobacco use disorder, glaucoma open-angle, hepatitis C|remission spontaneous **GAD Class**: Chemdependency, vision, infection
*AK5*	**KEGG**: Purine metabolism, metabolic pathways, biosynthesis of antibiotics **Reactome:** Interconversion of nucleotide di- and triphosphates **GAD:** Heart failure, leukocyte count, sodium, stroke, tobacco use disorder **GAD class:** Cardiovascular, chemdependency, hematological, metabolic, neurological
*NOG*	**KEGG:** TGF-beta signaling pathway **Reactome:** Signaling by BMP (Bone morphogenetic proteins) **GAD:** Albumins, body height, bone mineral density, cleft lip, gamma-glutamylcyclotransferase, height, neural tube defects, nonsyndromic cleft lip with or without cleft palate, obesity|POF–premature ovarian failure|polycystic ovarian syndrome|polycystic ovary syndrome|primary ovarian insufficiency|puberty, delayed|puberty, precocious|thrombophilia|tobacco use disorder, osteoporosis **GAD class:** developmental, metabolic
*ZSCAN18*	**KEGG:** Purine metabolism, morphine addiction **Reactome:** G alpha (s) signaling events **GAD:** Hemoglobins, HIV-1, tobacco use disorder, type 2 diabetes|edema|rosiglitazone **GAD class:** chemdependency, hematological, infection, pharmacogenomics
*Gene Ontology terms associated with at least two genes (EASE score < 0.1)*	
**Category**	**Terms**
GO biological process	Anion transport, developmental growth, chloride transport, central nervous system development, inorganic anion transport, growth, neuron development, regulation of cell size, anion transmembrane transport, sodium ion transport
GO molecular function	Secondary active transmembrane transporter activity, active transmembrane transporter activity, inorganic anion transmembrane transporter activity, symporter activity, substrate-specific transmembrane transporter activity, transmembrane transporter activity, anion transmembrane transporter activity, substrate-specific transporter activity
GO cellular component	Integral component of plasma membrane, intrinsic component of plasma membrane, plasma membrane region, basolateral plasma membrane, plasma membrane part

Analysis was performed using DAVID 6.8 database and categories including Kyoto Encyclopedia of Genes and Genomes (KEGG), Reactome, Genetic Association Database (GAD), Genetic Association Database Class (GAD Class), and Gene Ontology (GO).

All analyzed genes, except *FBLN2*, were associated with at least one term linked to atherosclerosis-related disease or risk factor, including heart failure, stroke, body weights and measures, cardiovascular, cholesterol LDL (low density lipoproteins), myocardial infarction, synthesis of phosphatidylglycerol, tobacco use disorder, type 2 diabetes, and obesity. Surprisingly, all but two genes (*FAM129A*, *SLC16A10*) were associated with chemical dependency, addictive diseases, and neurological disorders. GO enrichment analysis assigned upregulated genes to phosphate-containing compound metabolic processes and downregulated genes were ascribed to transmembrane transport of chloride and sodium ions (**Table 4**).

## Discussion

Despite great advances in cardiovascular research, PAD related diseases (including LEAD) still represent the major health problem with serious clinical complications. Investigation and treatment of LEAD has been hindered by the multifactorial character of the disease, diverse symptomatology, lack of relevant *in vitro* disease models and problems with acquiring suitable specimens. There is a need for novel, low-invasive biomarkers for early detection of LEAD and monitoring disease progression.

In presented study we conducted comparative analysis of microRNAome and transcriptome from PBMCs of patients with LEAD and healthy controls. Expression profiles were determined by NGS. Integrated analysis of microRNAome and transcriptome is important for our understanding of miRNA functions, giving specific insights into a broad layer of post-transcriptional control (Rajewsky, 2006). Identification of miRNA influenced gene expression patterns facilitates linking specific miRNA:genes interaction networks associated with this disease.

Our experiment involved utilization of statistical and bioinformatical tools to analyze miRNA and gene expression datasets and to determine miRNA:gene regulatory network. We applied strict rules for elimination or alleviation of technical, detection, and biological biases (Hansen et al., 2011; Timmons et al., 2015)- for detailed description of laboratory and other procedures please refer to Supplementary Material. We selected most promising 26 miRNAs and 14 genes, which potentially may serve as biomarkers for LEAD (**Tables 2** and **3**, respectively). The threshold of statistical significance was elevated to *P* < 0.0001 for miRNA selection with DESeq2 analysis to limit false positive results. Elimination of uninformative variables using UVE-PLS allowed us to present more reliable results. Those criteria for miRNA and gene signatures selection were introduced to eliminate qPCR validation. ROC analysis confirmed good diagnostic value of proposed biomarkers (**Tables 2** and **3**, **Supplementary Tables 8** and **11**, **Supplementary Figures 8** and **11**). Determined miRNA:gene interactions formed a proposed regulatory network (**Figure 4**), although further confirmation of predictive interactions should be performed in future studies. The preliminary functional analysis suggests that proposed biomarkers may provide useful information on the pathogenesis of LEAD.

Euclidean clustering and PCA analysis of determined potential biomarker miRNAs clearly segregates studied individuals into LEAD and control groups, but after excluding four initially selected miRNA transcripts (hsa-mir-486-2_hsa-miR-486-3p, hsa-mir-486_hsa-miR-486-5p, hsa-mir-486_hsa-miR-486-3p, hsa-mir-486-2_hsa-miR-486-5p) which belong to miR-486 family (compare **Figures 2C, D** with **Supplementary Figure 7**). One of the reasons for the occurrence of this phenomenon can be erythrocyte contamination and/or hemolysis in studied samples, since miR-486 has been reported as erythrocyte miRNA (Pizzamiglio et al., 2017). However, that process was not observed in studied blood samples or PBMCs preparations. PBMCs isolation procedure included four washing steps, in order to avoid erythrocyte contamination of PBMCs samples. The other reason could be the influence of factors, like age, BMI, sex, or smoking habits, which may affect expression of miR-486 family transcripts. Indeed, correlation analysis indicated age, BMI, and smoking habits as influencing factors (**Supplementary Table 7**), what may explain weaker biomarker correlation of these miRNA transcripts despite high statistical significance of differentiation LEAD and control groups.

The idea of creating panels and monitoring peripheral atherosclerosis-associated blood biomarkers, including miRNA profiling, is not novel and has been well studied (Patino et al., 2005; Mohr and Liew, 2007; Zampetaki et al., 2012). Stather et al. indicated 12 miRNAs with good diagnostic value in PAD by profiling 754 miRNAs in peripheral blood using quantitative RT-PCR (Stather et al., 2013). Using the same method, Signorelli et al. indicated association between presence of PAD and increased serum level of miR-130a, miR-27b, and miR-210, showing significant correlation between BMI and miR-130a, as well as between claudication distance and miR-210 (Signorelli et al., 2016). Overexpression of these three miRNAs in serum may also serve as early marker of a PAD-related disease—atherosclerosis obliterans (Li et al., 2011). Vegter et al. demonstrated relationship between downregulation of miR-18a-5p, miR-27a-3p, miR-199a-3p, miR-223-3p, and miR-652-3p in plasma and severity of PAD symptoms in patients with heart failure (Vegter et al., 2017).

Huang and collaborators, using massively parallel sequencing of plasma miRNAs, showed that downregulation of miR-125b is associated with increased occurrence of acute myocardial infarction (AMI) in Chinese cohorts (Huang et al., 2014). Therefore, lower level of this miRNA in our LEAD group may be a sign of higher risk of cardiac complications.

Rationale for use of PBMCs in our study was the fact, that this cell pool was not studied extensively in LEAD. PBMCs represent a subpopulation of white blood cells containing lymphocytes and monocytes, constituting an important element of inflammation process in atherosclerosis. Transcriptional profiling of this subpopulation should provide an abundance of information about vascular occlusive diseases. PBMCs are also highly accessible, what facilitates their utilization in medical procedures.

Dong et al. reported that increased expression levels of miR-24, miR-33a, miR-103a, miR-122, miR-34a, and miR-21 in PBMCs are indicators of lipids levels in stable CAD (Dong et al., 2017). In that study, miR-34a and miR-21 differed insignificantly between CAD and control groups, but in other studies both miRNAs were significantly upregulated in plasma and atherosclerotic plaques of CAD patient (Raitoharju et al., 2011; Han et al., 2015). Therefore, overexpression of miR-34a and miR-21 in PBMCs, found in our study, may distinguish LEAD from CAD. This hypothesis needs to be confirmed in further studies.

Genes recognized as targets of biomarker miRNAs were already presented to play a role in pathology of atherosclerosis. Elevated expression of *CDS2* promotes synthesis of diacylglycerol and increase in lipid droplets formation in HeLa cells (Qi et al., 2016). Upregulation of *CDS2*, noticed in our study, presumably indicates intense lipid synthesis promoting foam cells formation in atherosclerosis plaques.

Downregulation of *SLC16A10* observed in our patients may suggest a decrease in uptake and secretion of thyroid hormones (Friesema et al., 2008), mimicking hypothyroidism, the condition associated with other atherosclerosis risk factors like elevated blood pressure and increased levels of LDL, cholesterol, C-reactive protein, and homocysteine (Ichiki, 2010).

Shankar et al. reported positive association between serum GGT (gamma-glutamyltransferase) levels and PAD in male patients (Shankar et al., 2008). Our study demonstrated upregulation of *GGT1* gene in patients with LEAD, the effect which may cause an increase in serum GGT, which confirms the association described by Shankar and collaborators.

*FAM129A* was reported to be dysregulated in atopy and asthma, where, similarly to atherosclerosis, inflammation is a prominent element of the disease (Yick et al., 2014). During asthma progression, airway undergoes a process of remodeling similar to atherosclerotic vascular wall transformation (Fixman et al., 2007). Recently, *FAM129A* was presented as an asthma steroid response modulator (McGeachie et al., 2018). This suggests, that similar pathologic mechanism, connecting epigenetic regulation of *FAM129A* expression, inflammation, and steroid metabolism, may play role in asthma and LEAD.

Pathological processes in LEAD manifest in different manner, following different pathways depending on the particular case scenario, which is not surprising as in a multifactorial disease comprising a plethora of environmental, genetic, and epigenetic factors. It is still unknown, how miRNA expression or influence of environmental factors may affect different presentation of the disease, warranting more research on those mechanisms.

In order to compare our data with current knowledge we have collected literature data in **Supplementary Table 14**. Surprisingly, out of 26 microRNA and 14 mRNA genes only seven microRNAs (let-7f-1-3p, miR-122, miR-34a-3p, miR-21-5p, miR-30e-3p, miR-125b-5p, miR-423-3p) overlapped with literature data as being important in atherosclerotic process. This could be easily explained by deep differences in methodology of studies performed to date. One can notice, that differences exist in almost all aspects of studies designs (please refer to **Supplementary Table 14** and **Supplementary Material**). Taken together, that will make direct comparison of data and scientific reasoning difficult and may explain mayor differences observed.

Although our research provides new elements of knowledge about application of miRNA and genes as biomarkers in LEAD, we are aware of several limitations:

1. It remains uncertain whether alterations of proposed biomarkers were predictive of or responsive to LEAD development.
2. PBMCs represent a heterogeneous pool of various cell subpopulations (lymphocytes, monocytes), which may differ in miRNA and gene expression patterns. To evaluate potential impact of those nuances we made deconvolution of miRNA and gene expression data. Results suggest minor influence of PBMCs subpopulations composition on sequencing outcome (**Supplementary Figures 9** and **12**). However, one considers further investigations of these differences.
3. Although the selected changes in microRNA and genes expression levels were highly significant (*P* < 0.0001 for miRNA, Benjamini-Hochberg correction, UVE-PLS confirmation), studies with larger cohorts should be performed to confirm our results.
4. Participants subjected to transcriptomic analysis represent only a part of population for miRNA expression analysis, what may provide not comprehensive data of gene signatures in LEAD.
5. Significant differences in sex, age, BMI, and smoking habits between LEAD and control groups as well as other factors in LEAD group, such as co-existing diagnoses (i.e. type 2 diabetes mellitus, other cardiovascular diseases) and medications could influence the outcome.
6. Majority of miRNA:gene interactions in presented miRNA regulatory network had a putative character and require further *in vitro* and *in vivo* validation in external studies

In the light of these limitations, further investigations in larger cohort studies are needed to validate discriminative capability of found biomarkers and to explore their biological relevance. Detailed description of environmental, behavioral, and clinical factors of studied subjects would allow shedding more light on the complexity of LEAD pathology.

## Data Availability Statement

All datasets generated for this study are included in the Data Sheet2_v1.xlsx file and can be found in the FigShare repository https://doi.org/10.6084/m9.figshare.9918773.v3

## Ethics Statement

The studies involving human participants were reviewed and approved by Ethics Committee at Medical University of Lublin (decision No. KE-0254/341/2015). The patients/participants provided their written informed consent to participate in this study.

## Author Contributions

AB-K and JK conceived and designed the study and coordinate the research team. TZ and MF performed data collection, examination of the patients, and provided biological material for the study. AS provided equipment for next generation sequencing and technical support for the study. DZ, KR, and PK performed biological material preparation and next generation sequencing. AB-K, DZ, KR, ŁK, and JB performed data analysis. AB-K, DZ, KR, DG, and MF wrote the manuscript. All the authors read and approved the final manuscript.

## Funding

This work was supported by Statutory Funds of the Medical University of Lublin (DS43 to AB-K) provided by the Polish Ministry of Science and Higher Education for Medical University of Lublin, Poland.

## Conflict of Interest

AB-K, DZ, KR, AS, TZ, MF, and JK are co-authors of patent application “New miRNA markers of arterial atherosclerosis and application of miRNA markers to diagnosis of arterial atherosclerosis” No. P. 424674, submitted to Polish Patent Office.

The remaining authors declare that the research was conducted in the absence of any commercial or financial relationships that could be construed as a potential conflict of interest.
