# Supplementary material for: Dysregulation of MicroRNA Regulatory Network in Lower Extremities Arterial Disease
Source: Front Genet. 2019 Nov 22;10:1200. doi: 10.3389/fgene.2019.01200 (PMC6892359; doi:10.3389/fgene.2019.01200)
Supplement: Supplementary file 1 [file DataSheet_1.docx]

Supplementary Material

# SUPPLEMENTARY DATA

## Peripheral Blood Mononuclear Cells (PBMCs) isolation

Isolation of PBMCs from whole blood samples was conducted by density gradient centrifugation using Gradisol L reagent (Aqua-Med, Poland).

Whole blood samples obtained from study individuals were collected into 2.7-mL S-Monovette vials (Sarstedt, Germany), containing K3 ethylenediaminetetraacetic acid (EDTA) as anticoagulant factor. Samples were directly submitted to PBMCs isolation by density gradient centrifugation using Gradisol L reagent (Aqua-Med, Poland). Whole blood samples were diluted in 1:1 ratio with PBS (phosphate-buffered saline, without calcium and magnesium ions, Biomed-Lublin, Poland) in sterile, 15-mL conical tubes (Falcon, Corning Science Mexico, Mexico). Diluted blood was gently layered with sterile disposable Pasteur pipette on 3 mL Gradisol L in a new, sterile, 15-mL conical tube. Tubes were centrifuged at 2,000 rpm for 30 minutes at room temperature (5810 R Centrifuge, Eppendorf, Germany) with acceleration and deceleration rates set to 3. Buffy coats containing PBMCs were harvested with a sterile Pasteur pipette and transferred to a new, sterile, 15-mL centrifuge tubes. Cells were gently re-suspended in 10 mL of PBS by pipetting. The tubes were centrifuged at 2,000 rpm for 10 minutes at room temperature (5810 R Centrifuge, Eppendorf, Germany). All but 0.5 mL of the supernatant was removed. Cell pellets were gently re-suspended in the remaining volume. Entire volume was distributed to several sterile, 1.5-mL centrifuge tubes (DNA LoBind Tube, Eppendorf, Germany). The tubes were filled up to 1.5 mL with PBS and inverted three times to wash the cells. Then, the tubes were centrifuged at 2,000 rpm for 10 minutes at 4^o^C (5415 R Centrifuge, Eppendorf, Germany). This step was remade 2 times in order to remove any residual erythrocytes and microvesicles. After last centrifugation, supernatant was discarded and the tubes with PBMCs pellets were stored in -80^o^C till the isolation of RNA.

## Ion Torrent Small RNA Plugin v5.0.5r3 analysis workflow

***Initial alignment of reads and microRNA count***

Reads are aligned to mature microRNAs from miRbase v21 using tmap or bowtie2. Whole genome (hg19) is used for alignment of microRNA reads unaligned to miRbase. Other RNA molecules reads (tRNAs, rRNAs, mRNAs etc...) are also aligned to hg19 and counted. microRNA reads are counted using featureCounts with options *Minimum overlap*: 15 and *Minimum MQV for microRNA alignment:* 0.

***microRNA read filtering***

Reads with minimum number of 3’ adaptor bases were filtered in. Advanced parameter *Minimum 3’ adaptor bases required for microRNA alignment step* was set to *> 0* (default full adaptor length is 30 base pairs). cutadapt was runned on all reads with or without identified 3’ adaptor in order to filtered out reads shorter than 15 bp (with advanced parameter: *Minimum read length*).

List of applied microRNA read alignment and read filtering advance parameters:

*Minimum 3 prime adaptor bases required for microRNA alignment step*: 0-30, 30 by default

*Maximum mismatches for a microRNA alignment* to be valid 1 by default

*Maximum indels for a microRNA alignment* to be valid 1 by default

*Maximum 5p soft clipping for a microRNA alignment* to be valid 0

*Maximum 3p soft clipping for a microRNA alignment* to be valid 3

*Minimum alignment length for a microRNA alignment* to be valid 16

***Rescue of unmapped reads***

Unmapped reads or reads that were not successfully aligned to miRBase or which did not have the required number of 3’ adaptor bases are further aligned to the whole genome (Rescue Reference) with bowtie2.

**1.3 Quality of data and bias management**

In experiments one can observe **technical, detection** and **biological** biases (Hansen et al., 2011, Timmons et al., 2015).

**Technical bias** may be caused when, for example, different lab workers are responsible for conducting experiments, elongated procedures are applied and day-to-day inconsistencies appear (Leek et al., 2010). Another element of technical variability are batch effects being an ubiquitous form of noise compromising individual measurements and challenging significance, exactness and precision of experimental data, thus questioning scientific conclusions draw from such distracted sources (Oytam et al., 2016). Although batch effects are not removed by normalization procedures and may affect both specific subsets of genes and different genes in different ways (Leek et al., 2010) one of advantages of RNAseq method is its relatively low technical variability, making single sequencing of biological sample sufficient for analysis (Marioni et al., 2008, Hansen et al., 2011).

We substantially decreased **technical bias** by introducing strict laboratory procedures.

Blood used in experiments was probed in Independent Public Clinical Hospital No. 1 in Lublin. This was advantageous because LEAD assessment was provided always by the same vascular surgeon (MF). The hospital is about 350 meters from the lab where PBMCs isolation and storage was conducted. KPR was present during blood probing and transported samples in cooling container immediately and directly to laboratory.

In order to avoid cross-contamination of biological material by PCR products originating from other analyses, it is advisable to separate spatially RNA isolation and sequencing (KPR, personal communication). Spatial separation of PBMCs isolation from blood and storage, RNA isolation and storage (“Laboratory 1”, three separate areas), library preparation, sequencing procedures and storage (“Laboratory 2”, three separate areas) was strictly maintained. Blood after delivery to the laboratory was proceeded immediately for PBMCs isolation and subsequent storage in -80ºC. RNA isolation was conducted either on the same day or day after PBMCs isolation and RNA was stored in -80ºC for later quality assessment and library preparation.

Usually a batch of 10-12 microRNA samples was randomly distributed between KPR and DPZ and proceeded for library preparation. According to library preparation manual it was impossible to complete library construction in one day, what prompted us to plan workflow accordingly: working day 1 – Agilent assessment of microRNA quality and ligation (16h - overnight); working day 2 – reverse transcription and magnetic beads clean-up of cDNA sample; working day 3 – amplification with barcoding and magnetic beads clean-up of barcoded library, storage. After acquiring at least 11 barcoded libraries, library assessment on HS DNA chips was conducted on working day 4. Working day 5 was the day of mathematical calculations of library dilutions and sequencing planning. On working day 6 library dilutions were prepared and planned number of barcoded libraries was loaded on 540 sequencing chips. Use of Ion Chef robot in library amplification, Ion Sphere Particles preparation and final 540 chip loading excluded human error during those procedures. Single Ion Chef run prepared two ion chips sequenced on working day 7 along with Ion Sphere Particles quality control. Usually working days 6 and 7 were on the following week. Same batches of Library preparation kits, Ion Chef kits and Sequencing kits were applied for the sequencing procedure.

Workflow checkpoints were introduced:

- microRNA quality and quantity assessment on Agilent small RNA chips (Supplementary Figure 2),
- total RNA quality and quantity assessment on Agilent 6000 picoRNA chips (Supplementary Figure 3),
- library quality and quantity assessment on Agilent HS DNA chips (Supplementary Figures 2) and Agilent DNA 1000 chips (Supplementary Figure 3),
- Ion Sphere Quality Control (Supplementary Table 1 and 2).

Sequencing data quality plots (Supplementary Figure 4 and 5) show similar values of Cook’s distances across samples pointing to high homogeneity of obtained data which indicates good technical experiment preparation.

Principal Component Analysis (PCA) is helpful in quantification of different variables (both biological and technical) effects on data. If PCA does not correlate with examined variables it may indicate existence of unknown cause of batch effects in the data (Leek et al. 2010). Groupings on PCA plots representing both microRNA and mRNA expression are well separated and cohesive. Clustering plots shows separate clusters for LEAD cases and controls. This may indicate minor (if any) influence of batch effects and existence of some degree of natural biological variability in our data.

**Detection bias** occurs when some genes might not be detected with satisfying reliability as being differentially expressed or not detected at all. This might be due to probed sequence specificity or technology applied. Today science community does not have tools to circumvent such bias. This requires technology, which would detect all transcripts with equal probability regardless of their abundance (Timmons et al. 2015). One of potential solutions of this problem might be good coverage of sequencing. It was shown, that satisfactory coverage for microRNA sequencing for differential expression is 1-2 × 10^6 reads aligned to miRBase (Metpally et al., 2013; Campbell et al., 2015) and 10-25 × 10^6 reads for transcriptome differential analysis (Liu Y. et al., 2014; ENCODE 2011 RNA-Seq). Our conclusions were drawn on average of 3,340,547.94 ± 1,718,496.22 micro RNA reads aligned to miRBase v21 (median 2,989,149.5) and average of 10,997,417.67 ± 2,834,110.87 reads mapped to genes for transcriptome analysis (median 10,812,464) (Supplementary Tables 1 and 2) being in line with literature consensus.

One deals with **biological bias** analyzing specialized tissue, where due to its function transcriptome expression patterns are already biased towards the factors identified by functional enrichment analysis (Timmons et al., 2015). Other source of biological bias may be biological variability within the probed group that will affect gene expression amongst different individuals. It can be sampled only by multiple biological samples measurements. Misleading and irreproducible significant results may occur when small number of biological replicates is proceeded (n ≤ 2) making impossible to know whether expression patterns are specific to individuals or are characteristic for study group (Hansen et al., 2011).

In order to cope with **biological bias** we applied higher statistical threshold as a default (FDR with Benjamini-Hochberg correction at p < 0.0001) as was suggested by Timmons et al. 2015. Moreover we applied UVE-PLS analysis removing uninformative variables (noise reduction). At the end we validated our microRNA and mRNA panel with ROC analysis. In comparison to the literature, we examined relatively large groups of subjects: 40 LEAD patients and 19 healthy controls for microRNA expression assessment and groups of 8 LEAD patients and 7 healthy controls for transcriptome assessment (Hansen 2011 et al., Supplementary Table 14). Importantly, cognitive value of presented research is high, due to the fact, that group of patients chosen for transcriptome sequencing was randomly selected from subjects already assessed for microRNA expression, giving unique opportunity for evaluating reciprocal regulation of miRNA::mRNA networks. This approach markedly reduced any technical or biological bias because all procedures were carried out on material originating from the same subjects, probed at the same time and in the same physiological condition.

**1.4 Associations of LEAD with other diseases.**

Functional analysis of eleven target genes (*AK5*, *CDS2*, *FAM129A*, *FBLN2*, *NOG*, *NRCAM*, *PDE7A*, *SLC12A2*, *SLC16A10*, *SLC4A10* and *ZSCAN18*), which undergo miRNA-mediated control of expression in LEAD, does not point directly to atherosclerosis solely. There are several GAD terms linked to nervous system disorders e.g. schizophrenia (*NRCAM*, *SLC12A2*), Alzheimer disease (*FBLN2*), autism (*NRCAM*) and neural tube defects (*NOG*) (Table 4). In our study, upregulated miR-548 family members (miR-548d-5p, miR-548t-3p and miR-548aa) appeared to be modulators of either of *FBLN2*, *NRCAM* or *SLC12A2* (Figure 4) indicating possible contribution of these miRNAs to cardiovascular and neurological diseases. This connection is supported by other study, where elevated expression of miR-34a and miR-548d, indicative for LEAD miRNAs found in our study, was described as biomarkers of schizophrenia (Lai et al., 2011). Schizophrenia was also reported as one of the risk factors for development of peripheral atherosclerosis (Hsu et al., 2016). Altered expression of other miRNA found as LEAD biomarker in our study, miR-34a-5p, was reported as plasma signature of Alzheimer’s disease (Cosín-Tomás et al., 2017).

Lipidome of the nervous system is extremely diverse, providing plasticity, fluidity and thickness of neuronal membranes (Ziegler et al., 2017). Dysregulation in the lipid pool may cause different disorder states. In cardiovascular system it may contribute to atherosclerosis and in nervous system it may promote neurological and neurodegenerative disorders (Lathe et al., 2014). Our results confirmed close relationship between atherosclerosis and nervous system diseases.

Functional analysis also showed association of seven genes (*AK5*, *CDS2*, *NRCAM*, *PDE7A*, *SLC12A2*, *SLC4A10* and *ZSCAN18*) with tobacco use disorder. Thus, discriminative character of those genes may be a result in smoking prevalence in LEAD group rather than LEAD presence. Smoking habits are well established risk factors of peripheral atherosclerosis and those seven genes could be involved in LEAD development through smoking habits related mechanisms.

**References for Supplementary Data**

Campbell, J.D., Liu, G., Luo, L., Xiao, J., Gerrein, J., Juan-Guardela, B., et al. (2015). Assessment of microRNA differential expression and detection in multiplexed small RNA sequencing data. *RNA* 21, 164-171. doi: 10.1261/rna.046060.114

Cosín-Tomás, M., Antonell, A., Lladó, A., Alcolea, D., Fortea, J., Ezquerra, M., et al. (2017). Plasma miR-34a-5p and miR-545-3p as Early Biomarkers of Alzheimer's Disease: Potential and Limitations. *Mol Neurobiol*. 54, 5550-5562. doi: 10.1007/s12035-016-0088-8

ENCODE 2011 RNA-Seq Guidelines: <https://www.encodeproject.org/documents/cede0cbe-d324-4ce7-ace4-f0c3eddf5972/@@download/attachment/ENCODE%20Best%20Practices%20for%20RNA_v2.pdf> (accession date 17.09.2019)

Hansen, K.D., Wu, Z., Irizarry, R.A., and Leek, J.T. (2011). Sequencing technology does not eliminate biological variability. *Nature biotechnology.* 29, 572–573. doi: 10.1038/nbt.1910

Hsu, W.Y., Lin, C.L., and Kao, C.H. (2016). A population-based cohort study on Peripheral Arterial Disease in patients with schizophrenia. *PLoS One*. 11, e0148759. doi: 10.1371/journal.pone.0148759

Lai, C.Y., Yu, S.L., Hsieh, M.H., Chen, C.H., Chen, H.Y., Wen, C.C., et al. (2011). MicroRNA expression aberration as potential peripheral blood biomarkers for schizophrenia. *PLoS One*. 6, e21635. doi: 10.1371/journal.pone.0021635

Lathe, R., Sapronova, A., and Kotelevtsev, Y. (2014). Atherosclerosis and Alzheimer—diseases with a common cause? Inflammation, oxysterols, vasculature. *BMC Geriatr*. 14, 36. doi: 10.1186/1471-2318-14-36

Leek, J.T., Scharpf, R.B., Bravo, H.C., Simcha, D., Langmead, B., Johnson, W. E., et al. (2010). Tackling the widespread and critical impact of batch effects in high-throughput data. *Nature reviews. Genetics* 11, 733-739. doi: 10.1038/nrg2825

Liu, Y., Zhou, J., and White, K.P. (2014). RNA-seq differential expression studies: more sequence or more replication?. *Bioinformatics* 30, 301-304. doi: 10.1093/bioinformatics/btt688

Marioni, J.C., Mason, C.E., Mane, S.M., Stephens, M., and Gilad, Y. (2008). RNA-seq: an assessment of technical reproducibility and comparison with gene expression arrays. *Genome research* 18, 1509-1517. doi: 10.1101/gr.079558.108

Metpally, R.P., Nasser, S., Malenica, I., Courtright, A., Carlson, E., Ghaffari, L., et al. (2013). Comparison of Analysis Tools for miRNA High Throughput Sequencing Using Nerve Crush as a Model. *Frontiers in Genetics* 4, 20. doi: 10.3389/fgene.2013.00020

Oytam, Y., Sobhanmanesh, F., Duesing, K., Bowden, J.C., Osmond-McLeod, M., and Ross, J. (2016). Risk-conscious correction of batch effects: maximising information extraction from high-throughput genomic datasets. *BMC bioinformatics* 17, 332. doi: 10.1186/s12859-016-1212-5

Timmons, J.A., Szkop, K.J., and Gallagher, I.J. (2015). Multiple sources of bias confound functional enrichment analysis of global -omics data. *Genome biology* 16, 186. doi: 10.1186/s13059-015-0761-7

Ziegler, A.B., Thiele, C., Tenedini, F., Richard, M., Leyendecker, P., Hoermann, A., et al. (2017). Cell-Autonomous Control of Neuronal Dendrite Expansion via the Fatty Acid Synthesis Regulator SREBP. Cell Rep. 21, 3346-3353. doi: 10.1016/j.celrep.2017.11.069

# SUPPLEMENTARY FIGURES AND TABLES

## Supplementary Figures


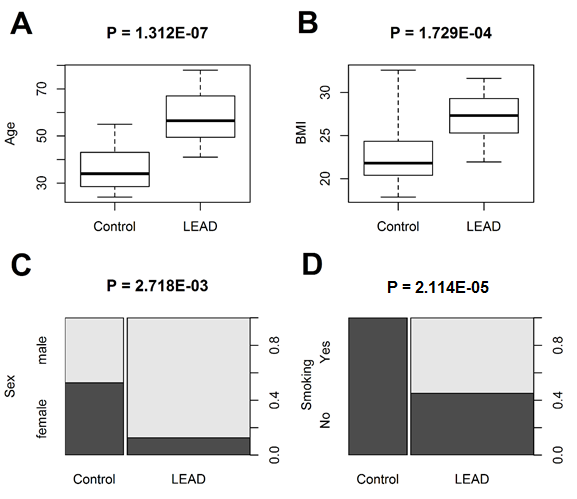


**Supplementary Figure 1:** Evaluation of differences between LEAD group and Control group for age, BMI, sex and smoking. *P* values were calculated using two-sided Mann-Whitney U test (for age and BMI) and two-sided Chi-square test (for sex and smoking). Boxplots present age **(A)** and BMI **(B)** distribution in studied groups. Whiskers define region between minimum and maximum values, boxes cover values between 25% and 75% quantile and horizontal lines inside boxes mark median value. **(C)** Spineplot presents percentage distribution of sex (black – female, gray – male) in studied groups. **(D)** Spineplot presents percentage distribution of smoking (black – no-smokers, grey – smokers) in studied groups.


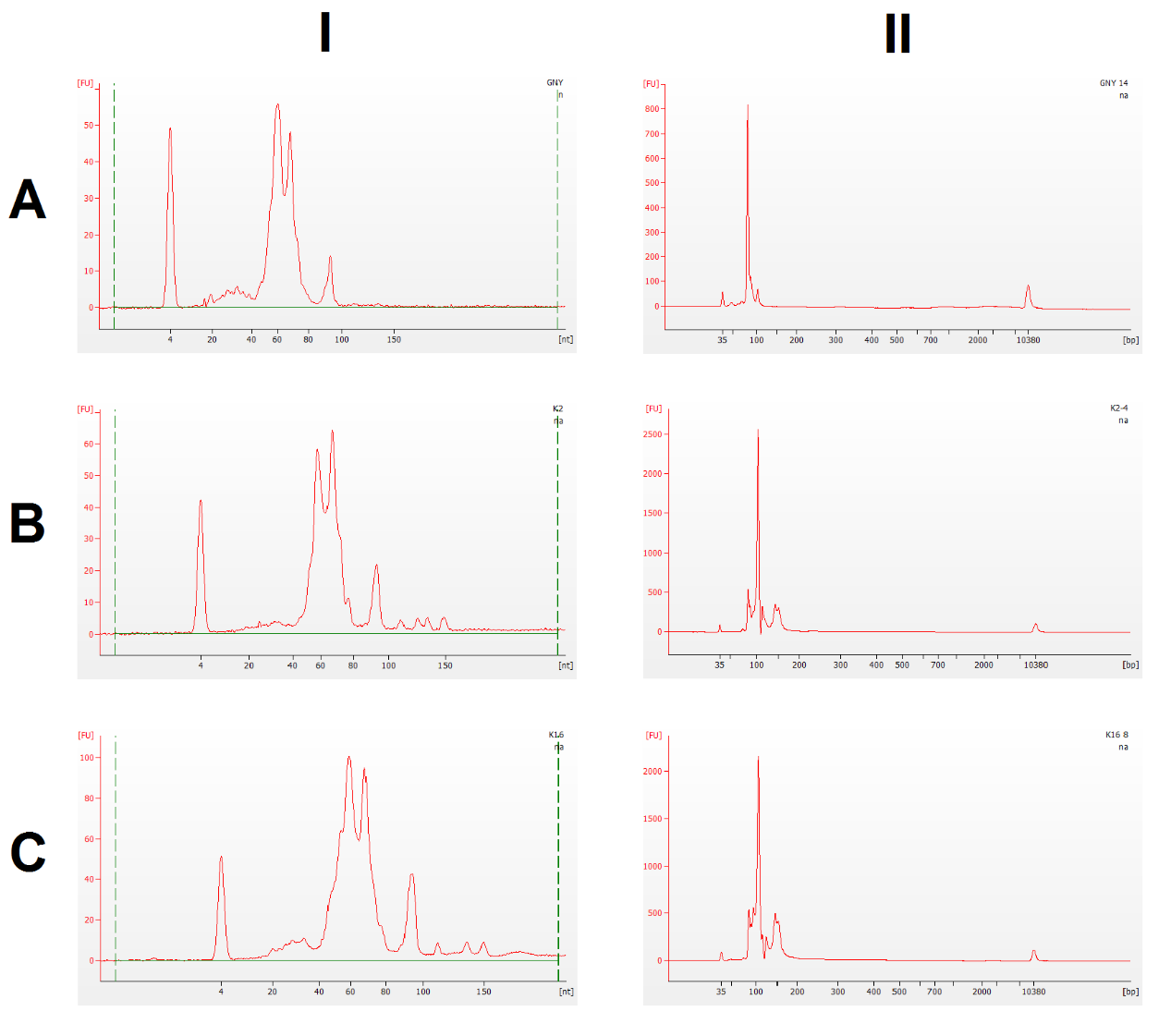


**Supplementary Figure 2:** Representative electrophoregrams of small RNA (**IA**, **IB** and **IC**) and corresponding libraries (**IIA**, **IIB** and **IIC**) of three randomly selected samples. Evaluation were performed using the Agilent 2100 Bioanalyzer instrument with the Agilent Small RNA Kit (for small RNA samples) and Agilent High Sensitivity DNA Kit (for small RNA libraries).


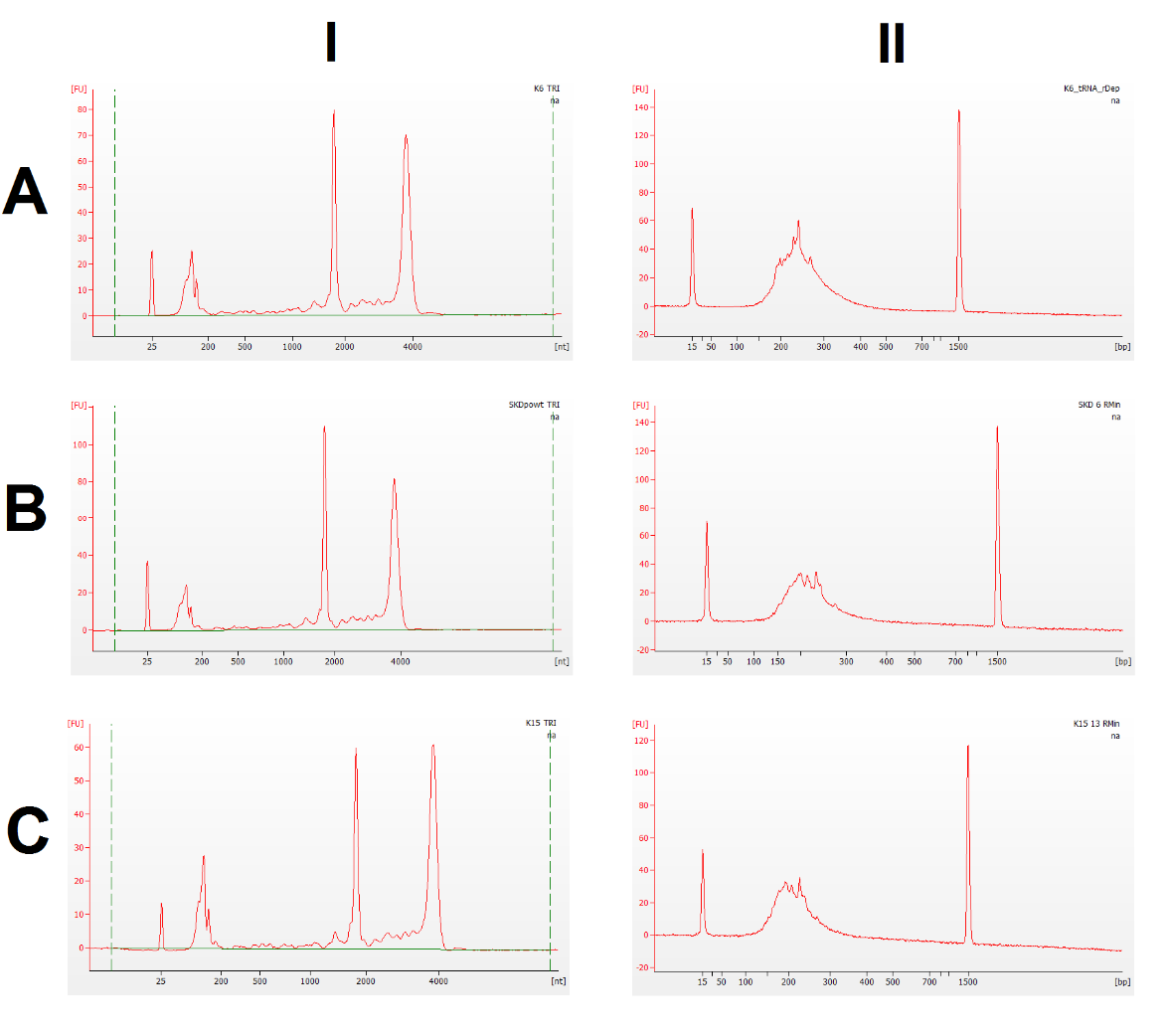


**Supplementary Figure 3:** Representative electrophoregrams of total RNA (**IA**, **IB** and **IC**) and corresponding libraries (**IIA**, **IIB** and **IIC**) of three randomly selected samples. Evaluations were performed using the Agilent 2100 Bioanalyzer instrument with the Agilent RNA 6000 Pico Kit (for total RNA samples) and Agilent DNA 1000 Kit (for libraries).


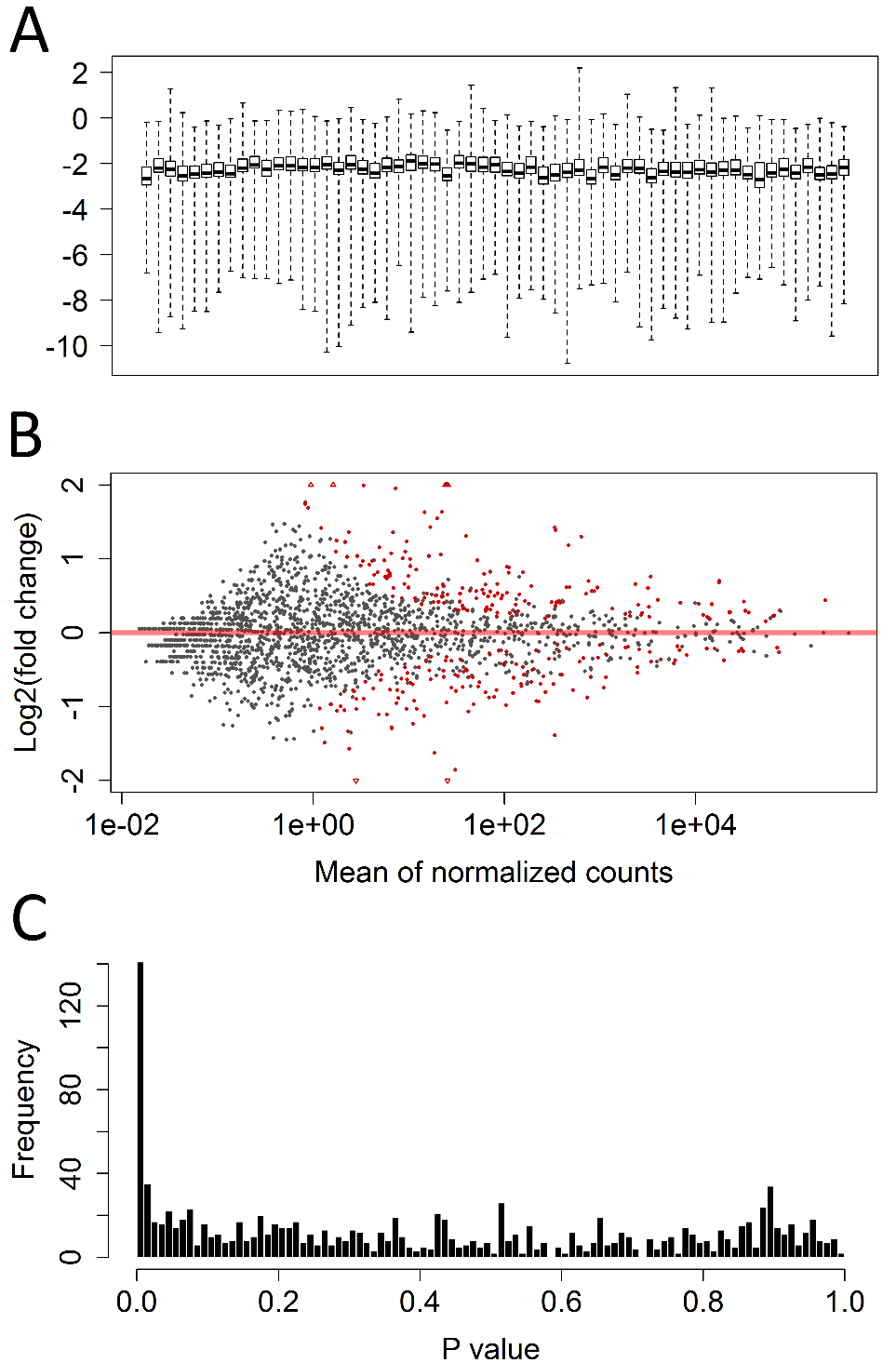


**Supplementary Figure 4:** Control plots for data obtained from sequencing of small RNA libraries. Boxplot (**A**) presents Cook’s distances across samples. Whiskers define the region between minimum and maximum value of Cook’s distance, boxes cover the region between 25% and 75% quartile, horizontal lines inside boxes mark median value. MA plot (**B**) shows log2 of fold changes of differentially expressed miRNAs over the average of normalized counts. Red points indicate miRNAs with *P* value < 0.1. **C,** Histogram of DESeq2 *p* values frequency.


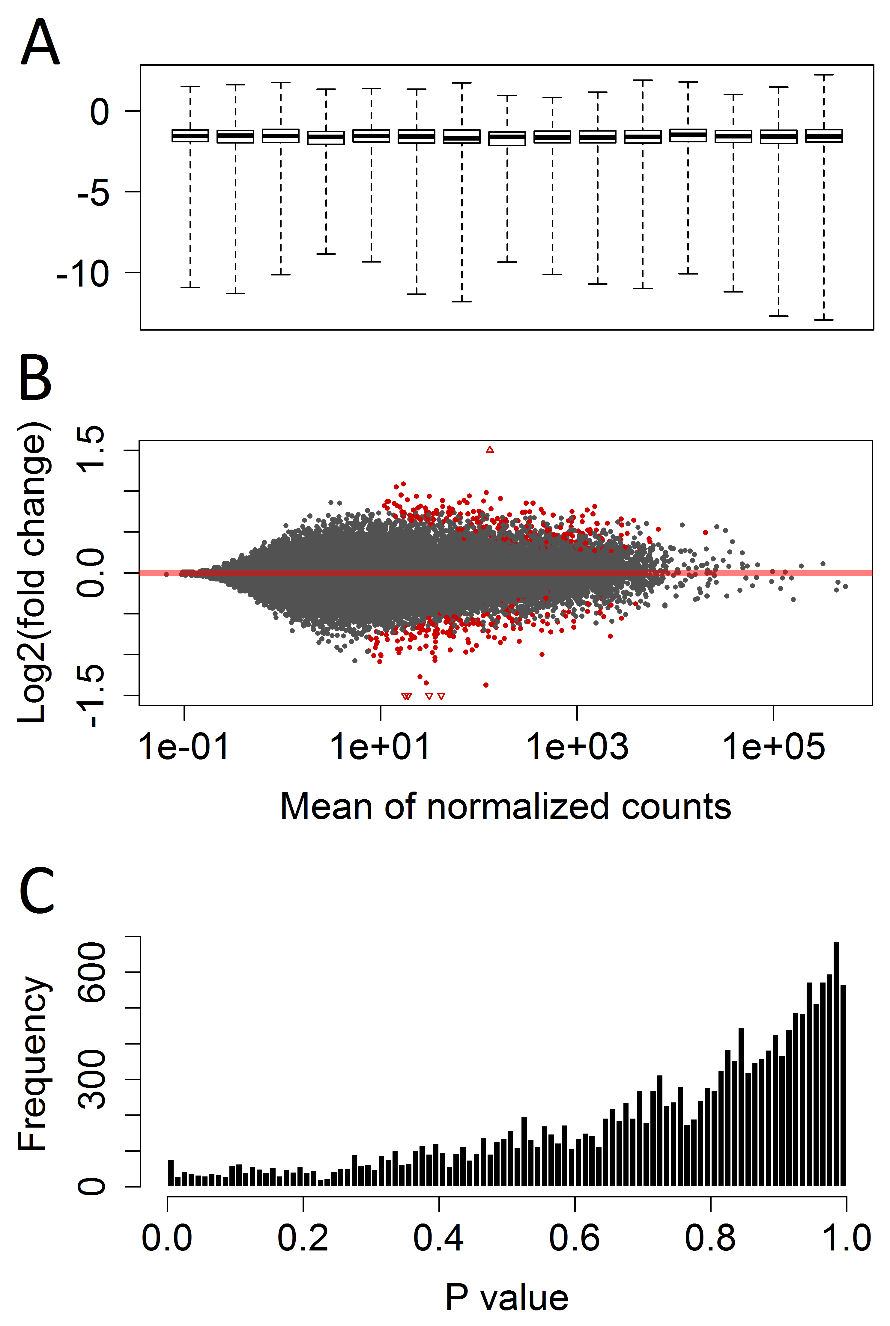


**Supplementary Figure 5:** Control plots for data from sequencing of transcriptome libraries. **A,** Boxplot presenting Cook’s distances across samples. Dashed lines define the region between minimum and maximum value of Cook’s distance, boxes cover the region between 25% and 75% quartile and horizontal lines inside boxes mark the median value. **B,** MA plot showing log2 of fold changes of differentially expressed genes over the average of normalized counts. Red points indicate genes with *P* value < 0.1. **C,** Histogram of DESeq2 *p* values frequency.


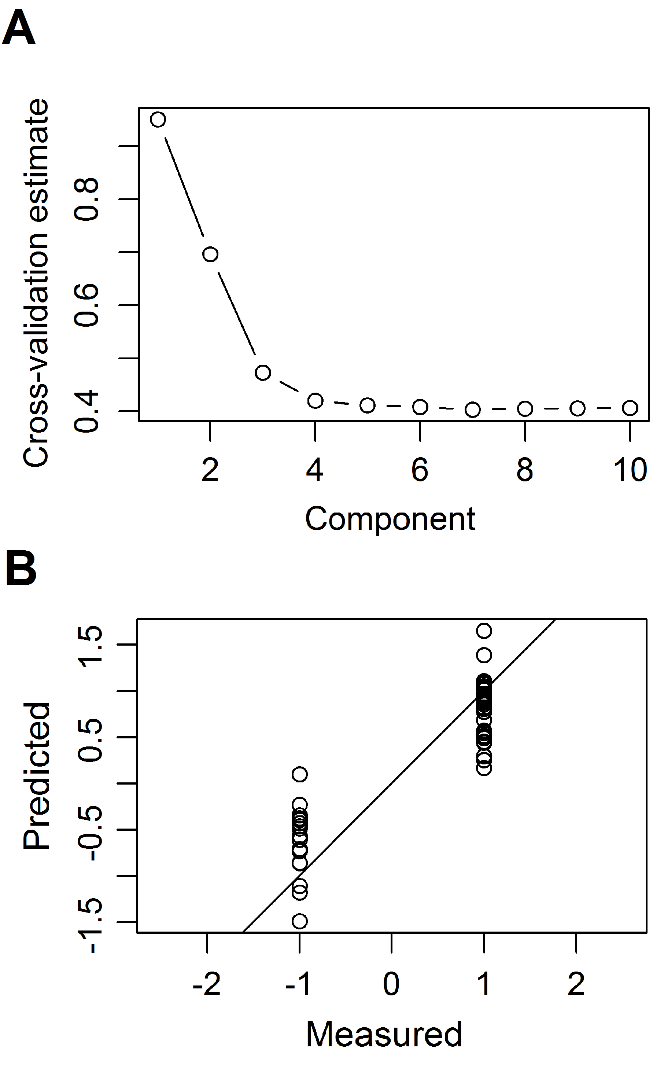


**Supplementary Figure 6:** MiRNA differential expression analysis in LEAD group in comparison to control group, performed using UVE-PLS method. **A,** Plot illustrating the arrangement of PLS component number and cross-validation estimates. **B,** Plot presenting a predictive ability of PLS model.


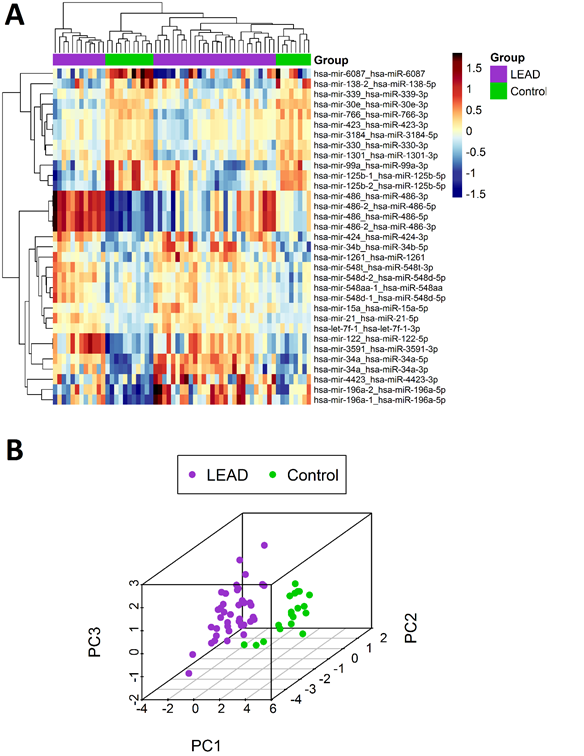


**Supplementary Figure 7:** : Heatmap with Euclidean clustering (**A**) and 3D PCA plot (**B**) generated based on expression of initially selected 33 differentially expressed miRNA transcripts in PBMCs samples derived from patients with LEAD (LEAD) and non-LEAD controls (Control).


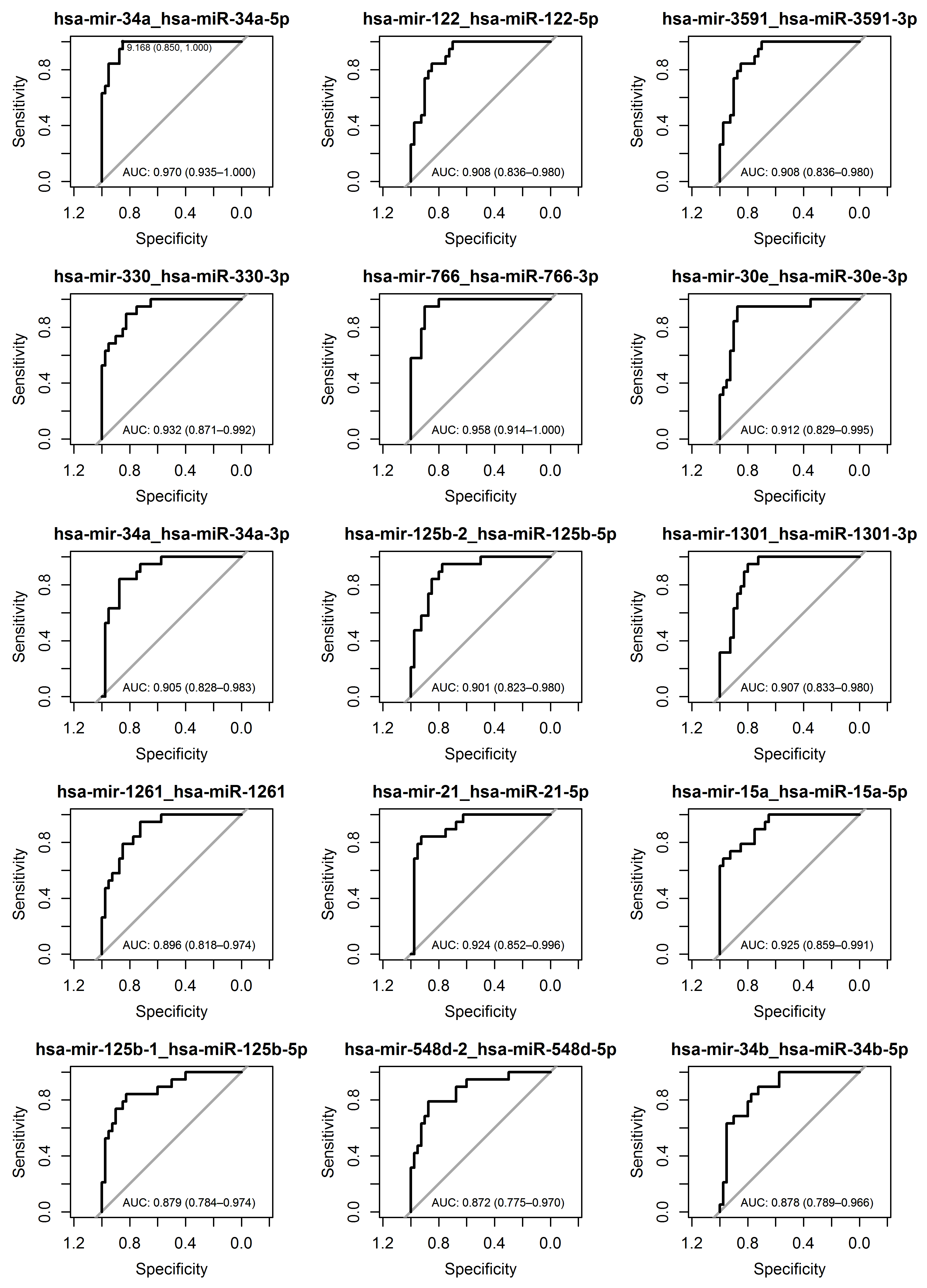


**Supplementary Figure 8:** Results of Receiver Operating Characteristics (ROC) analysis of 29 miRNA transcripts selected as indicative for LEAD. Plots present ROC curves and areas under curves (AUC) with 95% confidence interval (in brackets).


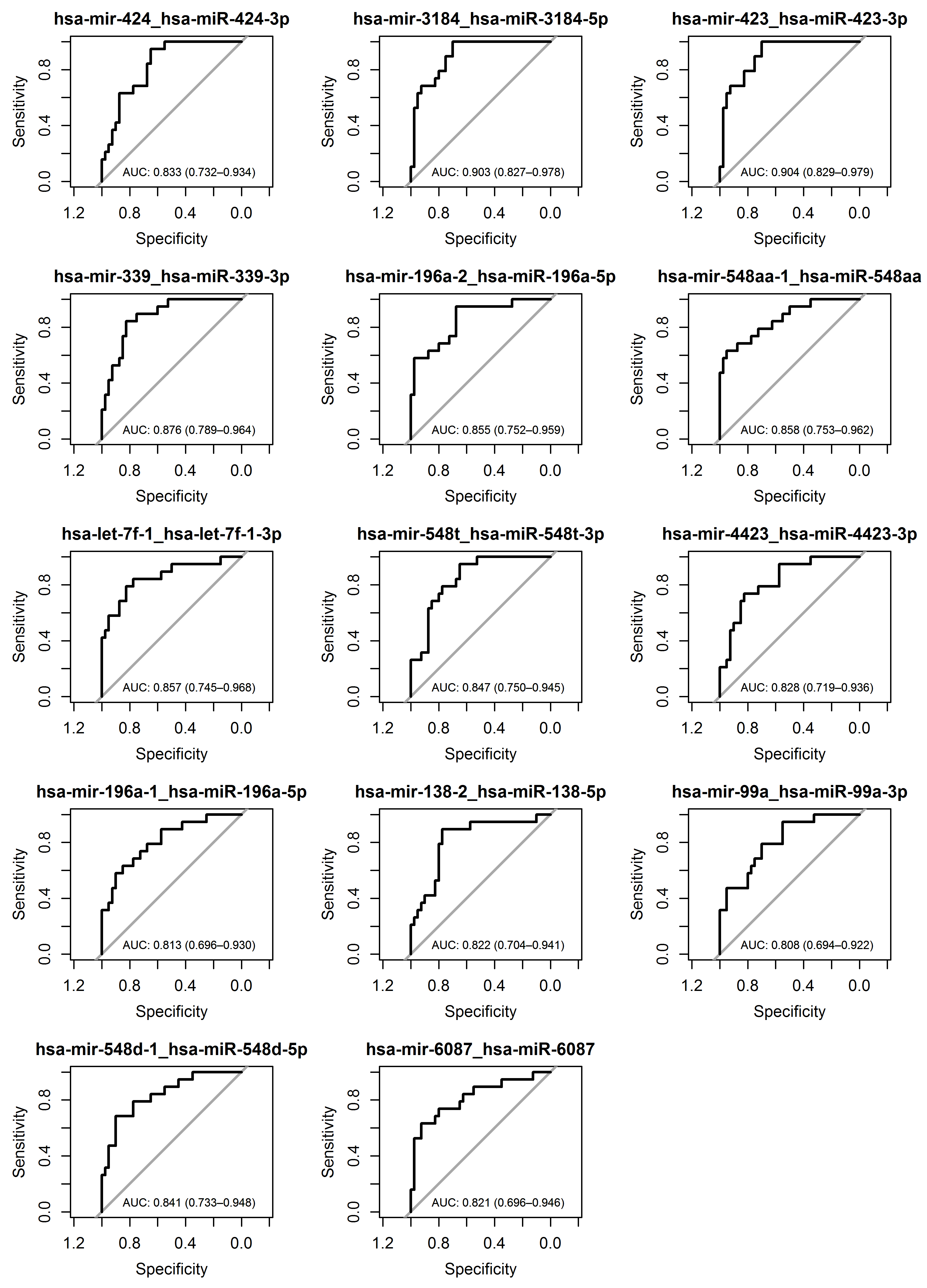


**Supplementary Figure 8:** (continued).


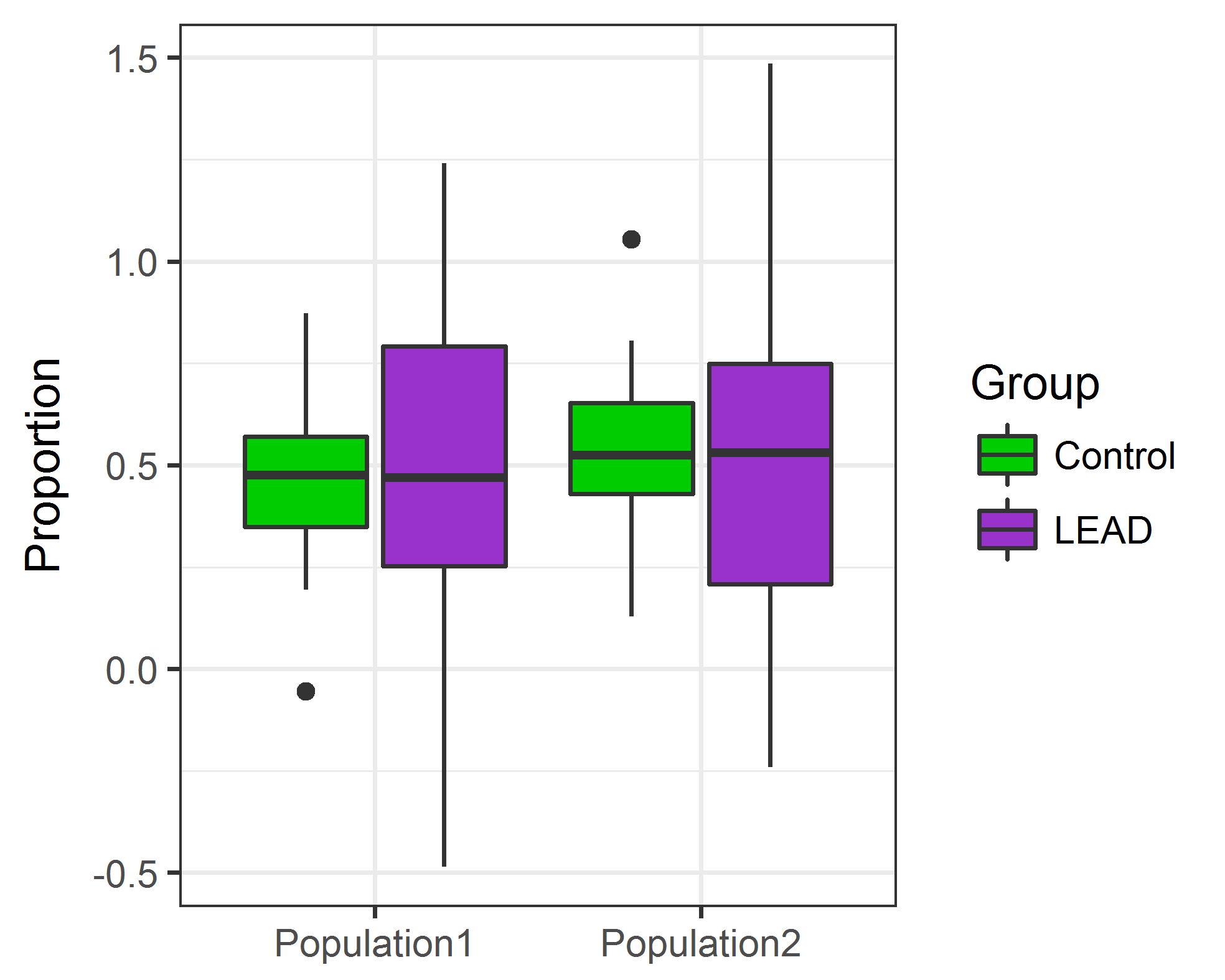


**Supplementary Figure 9.** Distribution of estimated proportions of two cell subpopulations in LEAD and Control groups.


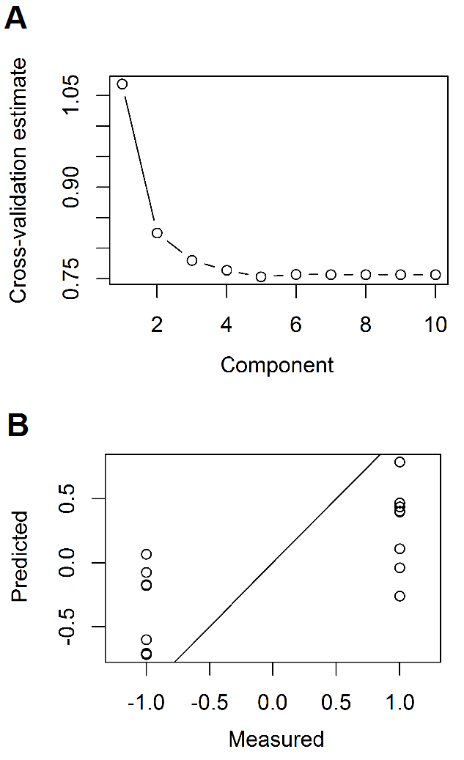


**Supplementary Figure 10:** Differential genes expression analysis in LEAD group in comparison to control group, performed using UVE-PLS method. **A,** Plot illustrating the arrangement of PLS component number and cross-validation estimates. **B,** Plot presenting a predictive ability of PLS model.


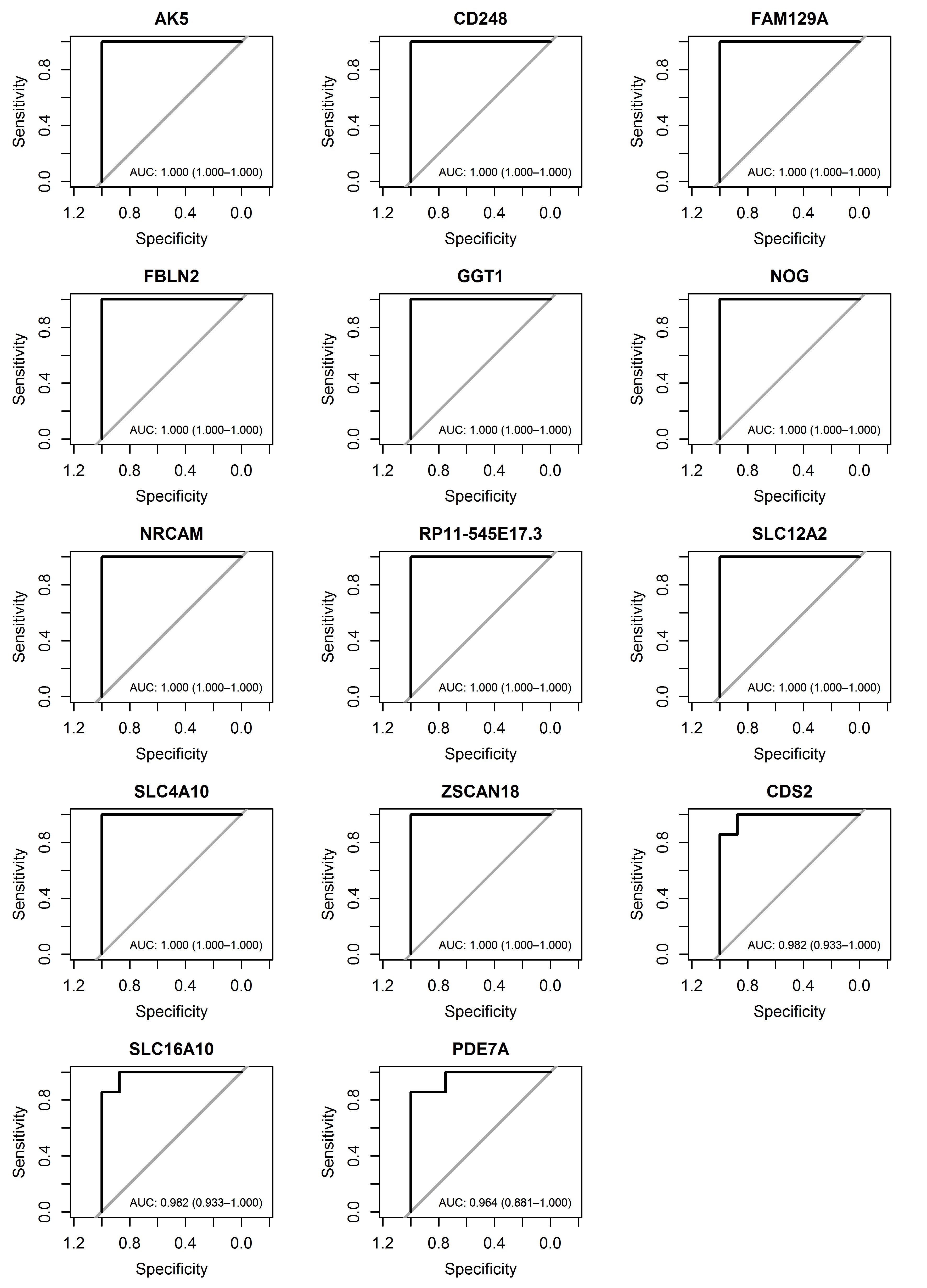


**Supplementary Figure 11:** Results of Receiver Operating Characteristics (ROC) analysis of 14 the most significantly differentially expressed genes in LEAD group in comparison to control group. Plots present ROC curves and areas under curves (AUC) with 95% confidence interval (in brackets).


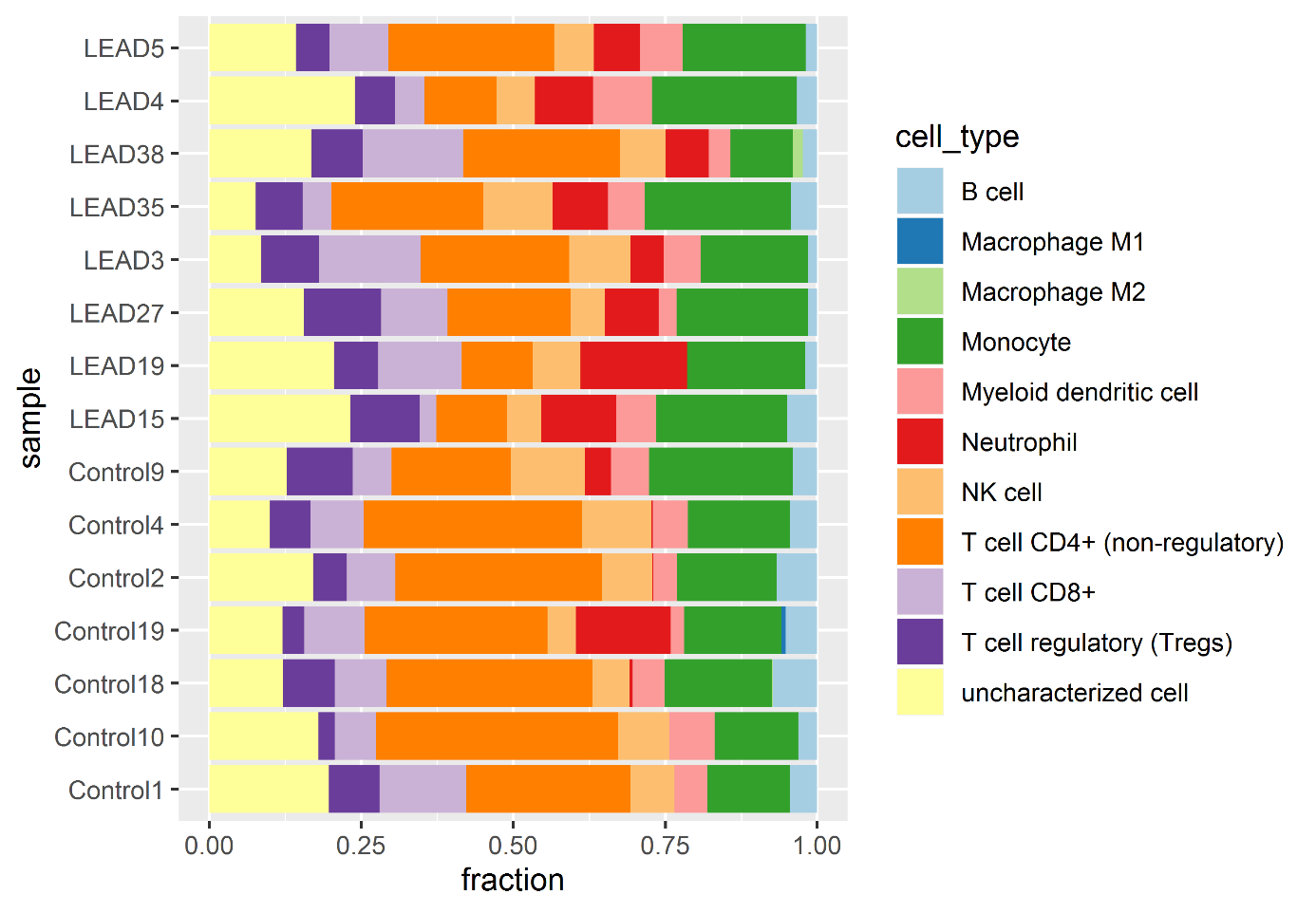


**Supplementary Figure 12.** Deconvolution results of gene expression data.

## Supplementary Tables

**Supplementary Table 1:** Parameters of small RNA samples, small RNA libraries and results of sequencing data primary analysis of small RNA libraries performed with Ion Torrent small RNA Plugin v5.0.5r3.

| **Parameter** | **Mean ± Standard Deviation** | **Median** |
| --- | --- | --- |
| Percentage of microRNA in small RNA samples | 18.1 ± 8.71 | 18 |
| Percentage of 94-114 bp^1^ region in small RNA libraries | 50.2 ± 6.87 | 49 |
| Molar concentration of small RNA libraries (pM) | 116,484 ± 56,834.36 | 121,442 |
| Ion Sphere Particles enrichment quality control | 32% ± 5.793% | 31% |
| Total number of reads | 8,999,655.35 ± 4,299,396.6 | 8,304,933 |
| Reads passing filter | 7,879,127.71 ± 3,965,311.27 | 7,286,090 |
| Number of aligned reads | 7,307,058.08 ± 3,716,607.61 | 6,806,400 |
| Percentage of aligned reads | 92.42% ± 1.6% | 92.68% |
| Number of reads aligned to mirBase v21 | 3,340,547.94 ± 1,718,496.22 | 2,989,149.5 |
| Percentage of reads aligned to mirBase v21 | 43.65% ± 10.48% | 43.81% |
| Number of reads aligned to precursors | 115,690.59 ± 70,460.21 | 109,584 |
| microRNAs with 1+ reads | 1,071.14 ± 379.04 | 1,199.5 |
| microRNAs with 10+ reads | 618.39 ± 86.06 | 624.5 |
| microRNAs with 100+ reads | 318.89 ± 54.32 | 321 |
| microRNAs with 1,000+ reads | 146.38 ± 26.59 | 147.5 |
| microRNAs with 10,000+ reads | 61.45 ± 17.24 | 66 |
| Non-uniquely mapped reads | 0 | 0 |
| No feature mappings | 607.65 ± 344.91 | 551.5 |
| Ambiguous mappings | 0 | 0 |
| Percentage of coding reads | 15.55% ± 4.31% | 15.04% |
| Percentage of rRNA reads | 0.73% ± 0.61% | 0.54% |
| Percentage of tRNA reads | 7.39% ± 5.87% | 5.22% |
| Percentage of snoRNA reads | 7.12% ± 4.19% | 6.25% |
| Percentage of snRNA reads | 0.99% ± 0.62% | 0.89% |
| Percentage of lincRNA reads | 1.06% ± 0.32% | 0.98% |
| Percentage of pseudogene reads | 0.26% ± 0.06% | 0.26% |
| ^1^base pair | | |

**Supplementary Table 2:** Parameters of transcriptome libraries and results of sequencing data primary analysis of transcriptome libraries performed with Ion Torrent RNASeqAnalysis plugin v.5.0.3.0.

| **Parameter** | **Mean ± Standard Deviation** | **Median** |
| --- | --- | --- |
| Molar concentration of libraries (pM) | 95,820 ± 19,008.24 | 96,800 |
| Percentage of 50-160 bp^1^ fragments in libraries | 10.8 ± 4.35 | 12 |
| Ion Sphere Particles enrichment quality control | 25.375% ± 4.406% | 27.5% |
| Total reads | 35,522,569.27 ± 2,982,842.56 | 34,853,503 |
| Aligned reads | 34,005,405.27 ± 2,926,723.36 | 33,038,587 |
| Percent aligned reads | 95.73% ± 1.56% | 95.98% |
| Mean read length | 111.08 ± 13.072 | 106.3 |
| Genes Detected | 14,625.33 ± 5,281.79 | 16,359 |
| Isoforms Detected | 47,142.27 ± 4,939.31 | 48,947 |
| Reads mapped to genes | 10,997,417.67 ± 2,834,110.87 | 10,812,464 |
| Genes with 1+ reads | 29,093.8 ± 7,308.93 | 30,863 |
| Genes with 10+ reads | 14,625.33 ± 5,281.79 | 16,359 |
| Genes with 100+ reads | 8,401.4 ± 2,298.27 | 9,286 |
| Genes with 1000+ reads | 1,648.07 ± 550.11 | 1,591 |
| Genes with 10000+ reads | 77.4 ± 17.64 | 74 |
| Isoforms Detected | 47,142.27 ± 4,939.31 | 48,947 |
| Total base reads | 3,939,321,714 ± 525,374,072.19 | 3,824,252,079 |
| Total aligned bases | 3,129,486,869 ± 410,961,931.82 | 3,037,508,241 |
| Percent aligned bases | 79.51% ± 3.1% | 78.34% |
| Percent coding bases | 12.97% ± 4.16% | 12.62% |
| Percent UTR^2^ bases | 24.81% ± 5.86% | 24.41% |
| Percent ribosomal bases | 6.08% ± 1.4% | 5.78% |
| Percent intronic bases | 39.99% ± 4.97% | 40.49% |
| Percent intergenic bases | 17.16% ± 5.42% | 16.35% |
| Strand balance | 50.13% ± 1.04% | 50.22% |
| ^1^base pair  ^2^Untranslated Region | | |

**Supplementary Table 3.** The set of 134 upregulated microRNA transcripts in LEAD group compared to healthy controls, resulted from DESeq2 analysis with *P* < 0.05. MicroRNA transcripts were ordered according to increasing *P* value.

| No. | microRNA transcript | *P* value | Fold change |
| --- | --- | --- | --- |
|  | hsa-mir-34a_hsa-miR-34a-5p | 1.585E-18 | 2.467 |
|  | hsa-mir-124-2_hsa-miR-124-3p | 6.088E-13 | 24.425 |
|  | hsa-mir-3591_hsa-miR-3591-3p | 1.087E-09 | 2.275 |
|  | hsa-mir-122_hsa-miR-122-5p | 1.087E-09 | 2.276 |
|  | hsa-mir-124-1_hsa-miR-124-3p | 1.196E-09 | 14.609 |
|  | hsa-mir-34a_hsa-miR-34a-3p | 1.937E-08 | 2.700 |
|  | hsa-mir-124-3_hsa-miR-124-3p | 2.056E-08 | 12.259 |
|  | hsa-mir-486_hsa-miR-486-5p | 3.923E-07 | 2.686 |
|  | hsa-mir-486-2_hsa-miR-486-3p | 3.923E-07 | 2.687 |
|  | hsa-mir-1261_hsa-miR-1261 | 7.061E-07 | 1.739 |
|  | hsa-mir-21_hsa-miR-21-5p | 7.287E-07 | 1.355 |
|  | hsa-mir-15a_hsa-miR-15a-5p | 8.637E-07 | 1.342 |
|  | hsa-mir-486-2_hsa-miR-486-5p | 1.184E-06 | 2.621 |
|  | hsa-mir-486_hsa-miR-486-3p | 1.184E-06 | 2.623 |
|  | hsa-mir-548d-2_hsa-miR-548d-5p | 1.897E-06 | 1.476 |
|  | hsa-mir-34b_hsa-miR-34b-5p | 2.138E-06 | 2.359 |
|  | hsa-mir-424_hsa-miR-424-3p | 2.538E-06 | 1.849 |
|  | hsa-mir-196a-2_hsa-miR-196a-5p | 4.356E-06 | 3.111 |
|  | hsa-mir-548aa-2_hsa-miR-548aa | 6.423E-06 | 1.450 |
|  | hsa-mir-542_hsa-miR-542-3p | 7.831E-06 | 1.763 |
|  | hsa-mir-548aa-1_hsa-miR-548aa | 8.363E-06 | 1.413 |
|  | hsa-mir-450b_hsa-miR-450b-5p | 1.404E-05 | 1.973 |
|  | hsa-let-7f-1_hsa-let-7f-1-3p | 1.486E-05 | 1.315 |
|  | hsa-mir-1537_hsa-miR-1537-3p | 1.762E-05 | 1.533 |
|  | hsa-mir-424_hsa-miR-424-5p | 2.162E-05 | 1.696 |
|  | hsa-mir-548t_hsa-miR-548t-3p | 2.446E-05 | 1.448 |
|  | hsa-mir-1268b_hsa-miR-1268b | 2.538E-05 | 2.055 |
|  | hsa-mir-4423_hsa-miR-4423-3p | 2.852E-05 | 3.873 |
|  | hsa-mir-196a-1_hsa-miR-196a-5p | 3.421E-05 | 3.099 |
|  | hsa-mir-454_hsa-miR-454-3p | 4.139E-05 | 1.253 |
|  | hsa-mir-548d-1_hsa-miR-548d-5p | 7.198E-05 | 1.405 |
|  | hsa-mir-450a-2_hsa-miR-450a-5p | 7.808E-05 | 1.860 |
|  | hsa-let-7b_hsa-let-7b-5p | 8.134E-05 | 1.308 |
|  | hsa-mir-548d-1_hsa-miR-548d-3p | 1.208E-04 | 1.544 |
|  | hsa-mir-362_hsa-miR-362-3p | 1.533E-04 | 1.625 |
|  | hsa-mir-19a_hsa-miR-19a-5p | 1.886E-04 | 1.572 |
|  | hsa-mir-576_hsa-miR-576-3p | 2.011E-04 | 2.001 |
|  | hsa-mir-34c_hsa-miR-34c-5p | 2.011E-04 | 2.143 |
|  | hsa-mir-548e_hsa-miR-548e-3p | 2.390E-04 | 1.486 |
|  | hsa-mir-590_hsa-miR-590-3p | 3.818E-04 | 1.299 |
|  | hsa-mir-7977_hsa-miR-7977 | 4.360E-04 | 1.349 |
|  | hsa-mir-450a-1_hsa-miR-450a-5p | 4.977E-04 | 1.759 |
| **Supplementary Table 3.** Continued | | | |
|  | hsa-let-7b_hsa-let-7b-3p | 5.777E-04 | 1.349 |
|  | hsa-mir-183_hsa-miR-183-5p | 7.230E-04 | 1.581 |
|  | hsa-let-7f-1_hsa-let-7f-5p | 7.971E-04 | 1.230 |
|  | hsa-mir-1246_hsa-miR-1246 | 8.952E-04 | 3.983 |
|  | hsa-mir-4791_hsa-miR-4791 | 1.007E-03 | 1.871 |
|  | hsa-mir-7641-1_hsa-miR-7641 | 1.215E-03 | 2.476 |
|  | hsa-mir-7847_hsa-miR-7847-3p | 1.230E-03 | 2.107 |
|  | hsa-mir-6503_hsa-miR-6503-3p | 1.350E-03 | 1.497 |
|  | hsa-mir-885_hsa-miR-885-5p | 1.463E-03 | 2.230 |
|  | hsa-mir-21_hsa-miR-21-3p | 1.691E-03 | 1.579 |
|  | hsa-mir-22_hsa-miR-22-3p | 1.698E-03 | 1.327 |
|  | hsa-mir-574_hsa-miR-574-5p | 1.739E-03 | 1.333 |
|  | hsa-mir-190b_hsa-miR-190b | 1.913E-03 | 1.520 |
|  | hsa-mir-7641-2_hsa-miR-7641 | 2.058E-03 | 2.924 |
|  | hsa-mir-629_hsa-miR-629-5p | 2.093E-03 | 1.303 |
|  | hsa-mir-199b_hsa-miR-199b-5p | 2.093E-03 | 1.480 |
|  | hsa-let-7a-3_hsa-let-7a-5p | 2.620E-03 | 1.214 |
|  | hsa-mir-9-2_hsa-miR-9-5p | 2.672E-03 | 1.405 |
|  | hsa-mir-3182_hsa-miR-3182 | 2.694E-03 | 2.083 |
|  | hsa-let-7a-2_hsa-let-7a-5p | 3.011E-03 | 1.212 |
|  | hsa-mir-9-3_hsa-miR-9-5p | 3.011E-03 | 1.403 |
|  | hsa-let-7a-1_hsa-let-7a-5p | 3.364E-03 | 1.208 |
|  | hsa-mir-374a_hsa-miR-374a-3p | 3.364E-03 | 1.444 |
|  | hsa-mir-449a_hsa-miR-449a | 4.103E-03 | 1.974 |
|  | hsa-mir-24-2_hsa-miR-24-3p | 4.176E-03 | 1.160 |
|  | hsa-mir-5582_hsa-miR-5582-3p | 4.176E-03 | 1.791 |
|  | hsa-mir-96_hsa-miR-96-5p | 4.176E-03 | 2.562 |
|  | hsa-let-7f-2_hsa-let-7f-5p | 4.330E-03 | 1.208 |
|  | hsa-mir-193a_hsa-miR-193a-5p | 4.704E-03 | 1.456 |
|  | hsa-mir-9-1_hsa-miR-9-5p | 4.709E-03 | 1.413 |
|  | hsa-mir-582_hsa-miR-582-5p | 4.709E-03 | 1.527 |
|  | hsa-mir-18b_hsa-miR-18b-5p | 4.812E-03 | 1.329 |
|  | hsa-let-7i_hsa-let-7i-5p | 4.833E-03 | 1.316 |
|  | hsa-mir-548f-2_hsa-miR-548f-3p | 5.239E-03 | 1.744 |
|  | hsa-mir-15a_hsa-miR-15a-3p | 5.401E-03 | 1.480 |
|  | hsa-mir-4458_hsa-miR-4458 | 5.486E-03 | 4.272 |
|  | hsa-mir-155_hsa-miR-155-3p | 5.659E-03 | 1.706 |
|  | hsa-mir-548f-3_hsa-miR-548f-3p | 5.820E-03 | 1.708 |
|  | hsa-mir-6503_hsa-miR-6503-5p | 5.844E-03 | 1.380 |
|  | hsa-mir-32_hsa-miR-32-5p | 5.941E-03 | 1.274 |
|  | hsa-mir-186_hsa-miR-186-5p | 6.858E-03 | 1.362 |
|  | hsa-mir-4802_hsa-miR-4802-5p | 7.730E-03 | 1.522 |
|  | hsa-mir-24-1_hsa-miR-24-3p | 1.046E-02 | 1.138 |
|  | hsa-mir-3074_hsa-miR-3074-5p | 1.047E-02 | 1.138 |
|  | hsa-mir-188_hsa-miR-188-3p | 1.050E-02 | 1.422 |
|  | hsa-mir-32_hsa-miR-32-3p | 1.132E-02 | 1.230 |
|  | hsa-mir-5100_hsa-miR-5100 | 1.170E-02 | 1.441 |
| **Supplementary Table 3.** Continued | | | |
|  | hsa-mir-624_hsa-miR-624-5p | 1.220E-02 | 1.226 |
|  | hsa-mir-548c_hsa-miR-548c-5p | 1.220E-02 | 1.243 |
|  | hsa-mir-624_hsa-miR-624-3p | 1.220E-02 | 1.445 |
|  | hsa-mir-1277_hsa-miR-1277-3p | 1.220E-02 | 1.533 |
|  | hsa-mir-548z_hsa-miR-548z | 1.270E-02 | 1.243 |
|  | hsa-mir-195_hsa-miR-195-5p | 1.282E-02 | 1.340 |
|  | hsa-mir-181b-1_hsa-miR-181b-3p | 1.411E-02 | 1.434 |
|  | hsa-mir-548ac_hsa-miR-548ac | 1.440E-02 | 1.702 |
|  | hsa-mir-676_hsa-miR-676-3p | 1.587E-02 | 4.073 |
|  | hsa-mir-140_hsa-miR-140-5p | 1.658E-02 | 1.197 |
|  | hsa-mir-3150b_hsa-miR-3150b-5p | 1.658E-02 | 3.356 |
|  | hsa-mir-3150a_hsa-miR-3150a-3p | 1.658E-02 | 3.356 |
|  | hsa-mir-4286_hsa-miR-4286 | 1.708E-02 | 1.182 |
|  | hsa-mir-186_hsa-miR-186-3p | 1.708E-02 | 1.216 |
|  | hsa-mir-503_hsa-miR-503-5p | 1.708E-02 | 1.477 |
|  | hsa-mir-548d-2_hsa-miR-548d-3p | 1.762E-02 | 1.324 |
|  | hsa-mir-4710_hsa-miR-4710 | 1.762E-02 | 2.348 |
|  | hsa-mir-582_hsa-miR-582-3p | 1.793E-02 | 1.420 |
|  | hsa-mir-548c_hsa-miR-548c-3p | 1.818E-02 | 1.727 |
|  | hsa-mir-497_hsa-miR-497-5p | 1.870E-02 | 1.551 |
|  | hsa-mir-193a_hsa-miR-193a-3p | 1.917E-02 | 1.434 |
|  | hsa-mir-25_hsa-miR-25-5p | 2.009E-02 | 1.897 |
|  | hsa-mir-19b-1_hsa-miR-19b-3p | 2.357E-02 | 1.174 |
|  | hsa-mir-503_hsa-miR-503-3p | 2.425E-02 | 2.055 |
|  | hsa-mir-33a_hsa-miR-33a-5p | 2.426E-02 | 1.161 |
|  | hsa-mir-616_hsa-miR-616-5p | 2.523E-02 | 1.254 |
|  | hsa-mir-200b_hsa-miR-200b-5p | 2.555E-02 | 1.806 |
|  | hsa-mir-1262_hsa-miR-1262 | 2.937E-02 | 3.234 |
|  | hsa-mir-92b_hsa-miR-92b-5p | 3.010E-02 | 1.960 |
|  | hsa-mir-148a_hsa-miR-148a-3p | 3.429E-02 | 1.207 |
|  | hsa-mir-4521_hsa-miR-4521 | 3.499E-02 | 1.574 |
|  | hsa-mir-1255a_hsa-miR-1255a | 3.728E-02 | 1.529 |
|  | hsa-mir-450a-2_hsa-miR-450a-2-3p | 3.910E-02 | 1.766 |
|  | hsa-mir-508_hsa-miR-508-3p | 3.910E-02 | 2.568 |
|  | hsa-mir-365b_hsa-miR-365b-3p | 4.113E-02 | 1.336 |
|  | hsa-mir-9-1_hsa-miR-9-3p | 4.173E-02 | 1.423 |
|  | hsa-mir-664a_hsa-miR-664a-5p | 4.261E-02 | 1.248 |
|  | hsa-mir-19b-2_hsa-miR-19b-3p | 4.573E-02 | 1.172 |
|  | hsa-mir-548au_hsa-miR-548au-5p | 4.573E-02 | 1.386 |
|  | hsa-mir-3154_hsa-miR-3154 | 4.573E-02 | 1.882 |
|  | hsa-mir-598_hsa-miR-598-3p | 4.601E-02 | 1.172 |
|  | hsa-mir-365a_hsa-miR-365a-3p | 4.601E-02 | 1.324 |
|  | hsa-mir-506_hsa-miR-506-5p | 4.601E-02 | 1.964 |
|  | hsa-mir-548h-4_hsa-miR-548h-3p | 4.719E-02 | 1.234 |
|  | hsa-mir-628_hsa-miR-628-5p | 4.806E-02 | 1.208 |

**Supplementary Table 4.** The set of 97 downregulated microRNA transcripts in LEAD group compared to healthy controls, resulted from DESeq2 analysis with *P* < 0.05. MicroRNA transcripts were ordered according to increasing *P* value.

| No. | microRNA transcript | *P* value | Fold change |
| --- | --- | --- | --- |
|  | hsa-mir-330_hsa-miR-330-3p | 3.735E-09 | 0.726 |
|  | hsa-mir-766_hsa-miR-766-3p | 4.262E-09 | 0.659 |
|  | hsa-mir-30e_hsa-miR-30e-3p | 1.540E-08 | 0.662 |
|  | hsa-mir-125b-2_hsa-miR-125b-5p | 3.537E-07 | 0.527 |
|  | hsa-mir-1301_hsa-miR-1301-3p | 3.923E-07 | 0.674 |
|  | hsa-mir-125b-1_hsa-miR-125b-5p | 1.039E-06 | 0.526 |
|  | hsa-mir-423_hsa-miR-423-3p | 2.588E-06 | 0.772 |
|  | hsa-mir-3184_hsa-miR-3184-5p | 2.588E-06 | 0.772 |
|  | hsa-mir-339_hsa-miR-339-3p | 3.649E-06 | 0.745 |
|  | hsa-mir-4488_hsa-miR-4488 | 4.052E-05 | 0.212 |
|  | hsa-mir-138-2_hsa-miR-138-5p | 4.052E-05 | 0.459 |
|  | hsa-mir-99a_hsa-miR-99a-3p | 7.039E-05 | 0.491 |
|  | hsa-mir-491_hsa-miR-491-5p | 7.778E-05 | 0.675 |
|  | hsa-mir-6087_hsa-miR-6087 | 8.461E-05 | 0.324 |
|  | hsa-mir-30e_hsa-miR-30e-5p | 1.381E-04 | 0.863 |
|  | hsa-mir-151a_hsa-miR-151a-3p | 1.886E-04 | 0.545 |
|  | hsa-mir-181c_hsa-miR-181c-5p | 1.890E-04 | 0.762 |
|  | hsa-mir-222_hsa-miR-222-3p | 2.011E-04 | 0.750 |
|  | hsa-mir-197_hsa-miR-197-3p | 4.174E-04 | 0.687 |
|  | hsa-mir-138-1_hsa-miR-138-5p | 5.777E-04 | 0.519 |
|  | hsa-mir-4284_hsa-miR-4284 | 8.415E-04 | 0.276 |
|  | hsa-mir-106b_hsa-miR-106b-3p | 8.415E-04 | 0.788 |
|  | hsa-mir-485_hsa-miR-485-3p | 8.974E-04 | 0.382 |
|  | hsa-mir-148b_hsa-miR-148b-3p | 9.393E-04 | 0.780 |
|  | hsa-mir-26a-2_hsa-miR-26a-5p | 1.398E-03 | 0.834 |
|  | hsa-mir-6836_hsa-miR-6836-3p | 1.428E-03 | 0.183 |
|  | hsa-mir-181c_hsa-miR-181c-3p | 1.530E-03 | 0.748 |
|  | hsa-mir-328_hsa-miR-328-3p | 1.873E-03 | 0.675 |
|  | hsa-mir-3960_hsa-miR-3960 | 2.094E-03 | 0.426 |
|  | hsa-mir-5193_hsa-miR-5193 | 3.279E-03 | 0.512 |
|  | hsa-mir-196b_hsa-miR-196b-3p | 3.279E-03 | 0.644 |
|  | hsa-mir-619_hsa-miR-619-5p | 3.347E-03 | 0.528 |
|  | hsa-mir-551b_hsa-miR-551b-3p | 3.364E-03 | 0.665 |
|  | hsa-mir-5585_hsa-miR-5585-3p | 3.485E-03 | 0.517 |
|  | hsa-mir-193b_hsa-miR-193b-3p | 3.485E-03 | 0.605 |
|  | hsa-mir-6516_hsa-miR-6516-5p | 3.535E-03 | 0.695 |
|  | hsa-mir-652_hsa-miR-652-5p | 3.632E-03 | 0.810 |
|  | hsa-mir-30d_hsa-miR-30d-5p | 4.453E-03 | 0.840 |
|  | hsa-mir-4742_hsa-miR-4742-3p | 4.605E-03 | 0.650 |
|  | hsa-mir-432_hsa-miR-432-3p | 4.709E-03 | 0.465 |
|  | hsa-mir-4429_hsa-miR-4429 | 4.742E-03 | 0.598 |
|  | hsa-mir-3940_hsa-miR-3940-3p | 4.812E-03 | 0.573 |
|  | hsa-mir-4523_hsa-miR-4523 | 4.872E-03 | 0.647 |
| **Supplementary Table 4.** Continued | | | |
|  | hsa-mir-6803_hsa-miR-6803-3p | 4.918E-03 | 0.708 |
|  | hsa-mir-6791_hsa-miR-6791-3p | 5.659E-03 | 0.356 |
|  | hsa-mir-665_hsa-miR-665 | 5.941E-03 | 0.507 |
|  | hsa-mir-331_hsa-miR-331-3p | 5.980E-03 | 0.783 |
|  | hsa-mir-874_hsa-miR-874-5p | 6.027E-03 | 0.616 |
|  | hsa-mir-26a-1_hsa-miR-26a-1-3p | 6.027E-03 | 0.662 |
|  | hsa-mir-548ax_hsa-miR-548ax | 7.207E-03 | 0.701 |
|  | hsa-mir-92a-1_hsa-miR-92a-3p | 7.399E-03 | 0.837 |
|  | hsa-mir-4492_hsa-miR-4492 | 7.900E-03 | 0.337 |
|  | hsa-mir-1973_hsa-miR-1973 | 7.900E-03 | 0.407 |
|  | hsa-mir-1250_hsa-miR-1250-5p | 7.944E-03 | 0.685 |
|  | hsa-mir-182_hsa-miR-182-5p | 9.215E-03 | 0.629 |
|  | hsa-mir-326_hsa-miR-326 | 9.433E-03 | 0.726 |
|  | hsa-mir-181d_hsa-miR-181d-5p | 9.433E-03 | 0.780 |
|  | hsa-mir-6772_hsa-miR-6772-3p | 1.047E-02 | 0.409 |
|  | hsa-mir-4792_hsa-miR-4792 | 1.170E-02 | 0.412 |
|  | hsa-mir-6802_hsa-miR-6802-3p | 1.488E-02 | 0.769 |
|  | hsa-mir-26a-1_hsa-miR-26a-5p | 1.549E-02 | 0.869 |
|  | hsa-mir-1273g_hsa-miR-1273g-3p | 1.747E-02 | 0.586 |
|  | hsa-mir-7110_hsa-miR-7110-3p | 1.833E-02 | 0.465 |
|  | hsa-mir-323b_hsa-miR-323b-3p | 1.833E-02 | 0.478 |
|  | hsa-mir-99a_hsa-miR-99a-5p | 1.950E-02 | 0.711 |
|  | hsa-mir-543_hsa-miR-543 | 1.993E-02 | 0.573 |
|  | hsa-mir-433_hsa-miR-433-3p | 2.009E-02 | 0.572 |
|  | hsa-mir-671_hsa-miR-671-3p | 2.099E-02 | 0.816 |
|  | hsa-mir-127_hsa-miR-127-3p | 2.189E-02 | 0.568 |
|  | hsa-mir-370_hsa-miR-370-3p | 2.413E-02 | 0.585 |
|  | hsa-mir-3617_hsa-miR-3617-5p | 2.413E-02 | 0.585 |
|  | hsa-mir-4659b_hsa-miR-4659b-3p | 2.523E-02 | 0.626 |
|  | hsa-mir-27b_hsa-miR-27b-3p | 2.523E-02 | 0.810 |
|  | hsa-mir-766_hsa-miR-766-5p | 2.583E-02 | 0.796 |
|  | hsa-mir-770_hsa-miR-770-5p | 2.678E-02 | 0.396 |
|  | hsa-mir-4659a_hsa-miR-4659a-5p | 2.733E-02 | 0.624 |
|  | hsa-mir-432_hsa-miR-432-5p | 3.026E-02 | 0.549 |
|  | hsa-mir-493_hsa-miR-493-3p | 3.044E-02 | 0.579 |
|  | hsa-mir-25_hsa-miR-25-3p | 3.044E-02 | 0.891 |
|  | hsa-mir-6894_hsa-miR-6894-3p | 3.429E-02 | 0.576 |
|  | hsa-mir-495_hsa-miR-495-3p | 3.429E-02 | 0.599 |
|  | hsa-mir-92a-2_hsa-miR-92a-3p | 3.516E-02 | 0.856 |
|  | hsa-mir-668_hsa-miR-668-3p | 3.581E-02 | 0.488 |
|  | hsa-mir-409_hsa-miR-409-3p | 3.581E-02 | 0.584 |
|  | hsa-mir-664a_hsa-miR-664a-3p | 3.581E-02 | 0.871 |
|  | hsa-mir-1301_hsa-miR-1301-5p | 3.687E-02 | 0.644 |
|  | hsa-mir-1254-2_hsa-miR-1254 | 4.011E-02 | 0.577 |
|  | hsa-mir-425_hsa-miR-425-5p | 4.093E-02 | 0.881 |
|  | hsa-mir-125a_hsa-miR-125a-5p | 4.172E-02 | 0.762 |
| **Supplementary Table 4.** Continued | | | |
|  | hsa-mir-431_hsa-miR-431-3p | 4.173E-02 | 0.543 |
|  | hsa-mir-4662b_hsa-miR-4662b | 4.173E-02 | 0.723 |
|  | hsa-mir-4662a_hsa-miR-4662a-5p | 4.173E-02 | 0.723 |
|  | hsa-mir-425_hsa-miR-425-3p | 4.173E-02 | 0.896 |
|  | hsa-mir-3617_hsa-miR-3617-3p | 4.402E-02 | 0.501 |
|  | hsa-mir-196b_hsa-miR-196b-5p | 4.601E-02 | 0.837 |
|  | hsa-mir-599_hsa-miR-599 | 4.647E-02 | 0.481 |
|  | hsa-mir-889_hsa-miR-889-3p | 4.679E-02 | 0.546 |

**Supplementary Table 5.** The set of 37 upregulated microRNA transcripts in LEAD group compared to healthy controls, resulted from UVE-PLS analysis. MicroRNA transcripts were ordered according to decreasing PLS coefficients.

| **No** | **microRNA transcript** | **PLS coefficient** |
| --- | --- | --- |
| 1. | hsa-mir-34a_hsa-miR-34a-5p | 4.297E-02 |
| 2. | hsa-mir-196a-2_hsa-miR-196a-5p | 3.913E-02 |
| 3. | hsa-mir-34a_hsa-miR-34a-3p | 3.786E-02 |
| 4. | hsa-mir-4423_hsa-miR-4423-3p | 3.686E-02 |
| 5. | hsa-mir-122_hsa-miR-122-5p | 3.216E-02 |
| 6. | hsa-mir-3591_hsa-miR-3591-3p | 3.213E-02 |
| 7. | hsa-mir-196a-1_hsa-miR-196a-5p | 3.036E-02 |
| 8. | hsa-mir-1277_hsa-miR-1277-3p | 2.747E-02 |
| 9. | hsa-mir-486_hsa-miR-486-5p | 2.669E-02 |
| 10. | hsa-mir-486-2_hsa-miR-486-3p | 2.668E-02 |
| 11. | hsa-mir-497_hsa-miR-497-5p | 2.275E-02 |
| 12. | hsa-mir-486_hsa-miR-486-3p | 2.254E-02 |
| 13. | hsa-mir-486-2_hsa-miR-486-5p | 2.251E-02 |
| 14. | hsa-mir-34b_hsa-miR-34b-5p | 2.235E-02 |
| 15. | hsa-mir-1261_hsa-miR-1261 | 1.978E-02 |
| 16. | hsa-mir-449a_hsa-miR-449a | 1.814E-02 |
| 17. | hsa-mir-1277_hsa-miR-1277-5p | 1.810E-02 |
| 18. | hsa-mir-9-2_hsa-miR-9-5p | 1.806E-02 |
| 19. | hsa-mir-576_hsa-miR-576-3p | 1.763E-02 |
| 20. | hsa-mir-34c_hsa-miR-34c-5p | 1.649E-02 |
| 21. | hsa-mir-362_hsa-miR-362-3p | 1.534E-02 |
| 22. | hsa-mir-374a_hsa-miR-374a-3p | 1.411E-02 |
| 23. | hsa-let-7b_hsa-let-7b-3p | 1.314E-02 |
| 24. | hsa-mir-424_hsa-miR-424-3p | 1.285E-02 |
| 25. | hsa-mir-15a_hsa-miR-15a-5p | 1.117E-02 |
| 26. | hsa-mir-548d-2_hsa-miR-548d-5p | 1.044E-02 |
| 27. | hsa-mir-22_hsa-miR-22-3p | 1.039E-02 |
| 28. | hsa-mir-548d-1_hsa-miR-548d-3p | 1.006E-02 |
| 29. | hsa-mir-19a_hsa-miR-19a-5p | 9.779E-03 |
| 30. | hsa-let-7f-1_hsa-let-7f-1-3p | 8.392E-03 |
| 31. | hsa-mir-32_hsa-miR-32-5p | 8.046E-03 |
| 32. | hsa-mir-548t_hsa-miR-548t-3p | 7.900E-03 |
| 33. | hsa-mir-21_hsa-miR-21-5p | 7.459E-03 |
| 34. | hsa-mir-590_hsa-miR-590-3p | 7.454E-03 |
| 35. | hsa-mir-548d-1_hsa-miR-548d-5p | 7.062E-03 |
| 36. | hsa-mir-33a_hsa-miR-33a-5p | 6.832E-03 |
| 37. | hsa-mir-548aa-1_hsa-miR-548aa | 6.820E-03 |

**Supplementary Table 6.** The set of 49 downregulated microRNA transcripts in LEAD group compared to healthy controls, resulted from UVE-PLS analysis. MicroRNA transcripts were ordered according to decreasing PLS coefficients.

| **No** | **microRNA transcript** | **PLS coefficient** |
| --- | --- | --- |
| 1. | hsa-mir-26a-2_hsa-miR-26a-5p | -3.747E-03 |
| 2. | hsa-mir-222_hsa-miR-222-3p | -4.699E-03 |
| 3. | hsa-mir-484_hsa-miR-484 | -4.935E-03 |
| 4. | hsa-mir-30d_hsa-miR-30d-5p | -5.084E-03 |
| 5. | hsa-mir-148b_hsa-miR-148b-3p | -5.501E-03 |
| 6. | hsa-mir-769_hsa-miR-769-5p | -6.567E-03 |
| 7. | hsa-mir-25_hsa-miR-25-3p | -6.674E-03 |
| 8. | hsa-mir-140_hsa-miR-140-3p | -7.176E-03 |
| 9. | hsa-mir-30e_hsa-miR-30e-5p | -7.488E-03 |
| 10. | hsa-mir-423_hsa-miR-423-3p | -7.793E-03 |
| 11. | hsa-mir-3184_hsa-miR-3184-5p | -7.793E-03 |
| 12. | hsa-mir-361_hsa-miR-361-3p | -8.301E-03 |
| 13. | hsa-mir-106b_hsa-miR-106b-3p | -8.531E-03 |
| 14. | hsa-mir-148b_hsa-miR-148b-5p | -8.868E-03 |
| 15. | hsa-mir-671_hsa-miR-671-5p | -9.463E-03 |
| 16. | hsa-mir-548ax_hsa-miR-548ax | -9.647E-03 |
| 17. | hsa-mir-92a-1_hsa-miR-92a-3p | -9.695E-03 |
| 18. | hsa-mir-652_hsa-miR-652-5p | -9.763E-03 |
| 19. | hsa-mir-92a-2_hsa-miR-92a-3p | -1.015E-02 |
| 20. | hsa-mir-326_hsa-miR-326 | -1.061E-02 |
| 21. | hsa-mir-551b_hsa-miR-551b-3p | -1.229E-02 |
| 22. | hsa-mir-151a_hsa-miR-151a-3p | -1.272E-02 |
| 23. | hsa-mir-181c_hsa-miR-181c-5p | -1.291E-02 |
| 24. | hsa-mir-150_hsa-miR-150-5p | -1.300E-02 |
| 25. | hsa-mir-330_hsa-miR-330-3p | -1.318E-02 |
| 26. | hsa-mir-328_hsa-miR-328-3p | -1.355E-02 |
| 27. | hsa-mir-30e_hsa-miR-30e-3p | -1.380E-02 |
| 28. | hsa-let-7g_hsa-let-7g-3p | -1.381E-02 |
| 29. | hsa-mir-342_hsa-miR-342-3p | -1.402E-02 |
| 30. | hsa-mir-766_hsa-miR-766-3p | -1.451E-02 |
| 31. | hsa-mir-6803_hsa-miR-6803-3p | -1.487E-02 |
| 32. | hsa-mir-1301_hsa-miR-1301-3p | -1.618E-02 |
| 33. | hsa-mir-197_hsa-miR-197-3p | -1.618E-02 |
| 34. | hsa-mir-125b-1_hsa-miR-125b-5p | -1.689E-02 |
| 35. | hsa-mir-99a_hsa-miR-99a-3p | -1.916E-02 |
| 36. | hsa-mir-339_hsa-miR-339-3p | -2.009E-02 |
| 37. | hsa-mir-99a_hsa-miR-99a-5p | -2.093E-02 |
| 38. | hsa-mir-125b-2_hsa-miR-125b-5p | -2.101E-02 |
| 39. | hsa-mir-485_hsa-miR-485-3p | -2.166E-02 |
| 40. | hsa-mir-196b_hsa-miR-196b-3p | -2.179E-02 |
| 41. | hsa-mir-874_hsa-miR-874-5p | -2.236E-02 |
| 42. | hsa-mir-6087_hsa-miR-6087 | -2.596E-02 |
| 43. | hsa-mir-3607_hsa-miR-3607-5p | -2.646E-02 |
| **Supplementary Table 6.** Continued | | |
| 44. | hsa-mir-5193_hsa-miR-5193 | -2.721E-02 |
| 45. | hsa-mir-31_hsa-miR-31-3p | -2.756E-02 |
| 46. | hsa-mir-31_hsa-miR-31-5p | -3.016E-02 |
| 47. | hsa-mir-193b_hsa-miR-193b-3p | -3.636E-02 |
| 48. | hsa-mir-138-2_hsa-miR-138-5p | -4.167E-02 |
| 49. | hsa-mir-138-1_hsa-miR-138-5p | -4.259E-02 |

**Supplementary Table 7:** Evaluation of the correlation between miRNA transcripts belonging to miR-486 family and risk factors of LEAD. Spearman rank correlation coefficients with *P* values (for age and BMI) and DESeq2 *P* values (for sex and smoking) were calculated. *P* values below 0.05 were marked with bold font.

| **miRNA transcript** | **miRNA ID^*^** | **Age^†^** | **BMI^†^** | **Gender** | **Smoking** |
| --- | --- | --- | --- | --- | --- |
|  |  |  |  |  |  |
| hsa-mir-486-2_hsa-miR-486-3p | hsa-miR-486-3p | 0.30 (**2.05E-02**) | 0.37 (**4.19E-03**) | 1.18E-01 | **2.26E-03** |
| hsa-mir-486_hsa-miR-486-5p | hsa-miR-486-5p | 0.30 (**2.90E-02**) | 0.37 (**3.70E-03**) | 1.18E-01 | **2.26E-03** |
| hsa-mir-486_hsa-miR-486-3p | hsa-miR-486-3p | 0.28 (**2.90E-02**) | 0.37 (**3.70E-03**) | 1.57E-01 | **2.26E-03** |
| hsa-mir-486-2_hsa-miR-486-5p | hsa-miR-486-5p | 0.28 (**2.04E-02**) | 0.37 (**3.96E-03**) | 1.57E-01 | **2.26E-03** |

^*^According to miRBase 22 (http://www.mirbase.org/)

^†^Spearman rank correlation coefficient (*P* value)

**Supplementary Table 8.** Results of ROC analysis of 33 selected miRNA transcripts.

| **miRNA transcript** | **ROC-AUC^1^** | **Threshold** | **Specificity** | **Sensitivity** | **Accuracy** | **Positive Predictive Value** | **Negative Predictive Value** |
| --- | --- | --- | --- | --- | --- | --- | --- |
| hsa-mir-34a_hsa-miR-34a-5p | 0.969 | 9.168 | 0.850 | 1.000 | 0.898 | 0.760 | 1.000 |
| hsa-mir-766_hsa-miR-766-3p | 0.957 | 10.245 | 0.900 | 0.947 | 0.915 | 0.818 | 0.973 |
| hsa-mir-330_hsa-miR-330-3p | 0.931 | 7.336 | 0.825 | 0.895 | 0.847 | 0.708 | 0.943 |
| hsa-mir-15a_hsa-miR-15a-5p | 0.925 | 14.790 | 0.925 | 0.737 | 0.864 | 0.824 | 0.881 |
| hsa-mir-21_hsa-miR-21-5p | 0.923 | 17.603 | 0.925 | 0.842 | 0.898 | 0.842 | 0.925 |
| hsa-mir-30e_hsa-miR-30e-3p | 0.911 | 9.757 | 0.875 | 0.947 | 0.898 | 0.782 | 0.972 |
| hsa-mir-122_hsa-miR-122-5p | 0.907 | 8.624 | 0.700 | 1.000 | 0.797 | 0.613 | 1.000 |
| hsa-mir-3591_hsa-miR-3591-3p | 0.907 | 8.624 | 0.700 | 1.000 | 0.797 | 0.613 | 1.000 |
| hsa-mir-1301_hsa-miR-1301-3p | 0.906 | 8.706 | 0.800 | 0.947 | 0.847 | 0.692 | 0.970 |
| hsa-mir-34a_hsa-miR-34a-3p | 0.905 | 3.661 | 0.875 | 0.842 | 0.864 | 0.761 | 0.921 |
| hsa-mir-423_hsa-miR-423-3p | 0.903 | 12.572 | 0.700 | 1.000 | 0.797 | 0.613 | 1.000 |
| hsa-mir-3184_hsa-miR-3184-5p | 0.902 | 12.572 | 0.700 | 1.000 | 0.797 | 0.613 | 1.000 |
| hsa-mir-125b-2_hsa-miR-125b-5p | 0.901 | 7.569 | 0.775 | 0.947 | 0.831 | 0.667 | 0.969 |
| hsa-mir-1261_hsa-miR-1261 | 0.896 | 5.811 | 0.725 | 0.947 | 0.797 | 0.621 | 0.967 |
| hsa-mir-125b-1_hsa-miR-125b-5p | 0.878 | 7.735 | 0.825 | 0.842 | 0.831 | 0.696 | 0.917 |
| hsa-mir-34b_hsa-miR-34b-5p | 0.877 | 2.997 | 0.725 | 0.895 | 0.780 | 0.607 | 0.935 |
| hsa-mir-339_hsa-miR-339-3p | 0.876 | 7.098 | 0.825 | 0.842 | 0.831 | 0.696 | 0.917 |
| hsa-mir-548d-2_hsa-miR-548d-5p | 0.872 | 6.005 | 0.875 | 0.789 | 0.847 | 0.750 | 0.897 |
| hsa-mir-548aa-1_hsa-miR-548aa | 0.857 | 6.068 | 0.950 | 0.632 | 0.847 | 0.857 | 0.844 |
| hsa-let-7f-1_hsa-let-7f-1-3p | 0.856 | 7.444 | 0.775 | 0.842 | 0.797 | 0.640 | 0.912 |
| hsa-mir-196a-2_hsa-miR-196a-5p | 0.855 | 4.111 | 0.675 | 0.947 | 0.763 | 0.581 | 0.964 |
| hsa-mir-548t_hsa-miR-548t-3p | 0.847 | 5.909 | 0.650 | 0.947 | 0.746 | 0.563 | 0.963 |
| hsa-mir-486-2_hsa-miR-486-3p | 0.842 | 8.335 | 0.550 | 1.000 | 0.695 | 0.514 | 1.000 |
| hsa-mir-486_hsa-miR-486-5p | 0.840 | 8.332 | 0.550 | 1.000 | 0.695 | 0.514 | 1.000 |
| hsa-mir-548d-1_hsa-miR-548d-5p | 0.840 | 5.888 | 0.900 | 0.684 | 0.831 | 0.765 | 0.857 |
| hsa-mir-424_hsa-miR-424-3p | 0.832 | 6.740 | 0.650 | 0.947 | 0.746 | 0.563 | 0.963 |
| hsa-mir-486_hsa-miR-486-3p | 0.831 | 8.048 | 0.675 | 0.895 | 0.746 | 0.567 | 0.931 |
| hsa-mir-486-2_hsa-miR-486-5p | 0.831 | 8.048 | 0.675 | 0.895 | 0.746 | 0.567 | 0.931 |
| hsa-mir-4423_hsa-miR-4423-3p | 0.827 | 2.061 | 0.825 | 0.737 | 0.797 | 0.667 | 0.868 |
| hsa-mir-138-2_hsa-miR-138-5p | 0.822 | 3.618 | 0.775 | 0.895 | 0.814 | 0.654 | 0.939 |
| hsa-mir-6087_hsa-miR-6087 | 0.821 | 4.245 | 0.925 | 0.632 | 0.831 | 0.800 | 0.841 |
| hsa-mir-196a-1_hsa-miR-196a-5p | 0.813 | 3.015 | 0.850 | 0.632 | 0.780 | 0.667 | 0.829 |
| hsa-mir-99a_hsa-miR-99a-3p | 0.807 | 3.270 | 0.550 | 0.947 | 0.678 | 0.500 | 0.957 |
| ^1^Area under ROC curve. | | | | |  |  |  |

**Supplementary Table 9.** 108 upregulated genes resulted from DESeq2 differential expression analysis with *P* < 0.05 in patients with LEAD, compared to healthy controls. Genes were ordered according to decreasing Fold change value.

| **Gene symbol** | **Gene name** | **Fold change** | ***P* value** | **Gene symbol** | **Gene name** | **Fold change** | ***P* value** |
| --- | --- | --- | --- | --- | --- | --- | --- |
| GPR15 | G protein-coupled receptor 15 | 3.7132 | 2.2E-12 | ACADVL | acyl-CoA dehydrogenase very long chain | 1.5172 | 2.345E-02 |
| SLC1A3 | solute carrier family 1 member 3 | 2.1269 | 3.04E-03 | CELSR3 | cadherin EGF LAG seven-pass G-type receptor 3 | 1.5101 | 3.864E-02 |
| HSD3B7 | hydroxy-delta-5-steroid dehydrogenase, 3 beta- and steroid delta-isomerase 7 | 2.0703 | 4.16E-03 | ITGAM | integrin subunit alpha M | 1.5022 | 5.486E-03 |
| MAP3K6 | mitogen-activated protein kinase kinase kinase 6 | 1.9753 | 8.93E-05 | RP4-647J21.1 | N/A | 1.4903 | 1.857E-03 |
| TPRG1-AS1 | TPRG1 antisense RNA 1 | 1.9354 | 6.863E-03 | RRAS | RAS related | 1.4853 | 4.037E-02 |
| MPO | myeloperoxidase | 1.9223 | 6.863E-03 | F5 | coagulation factor V | 1.4851 | 3.11E-02 |
| RP5-1109J22.1 | N/A | 1.9175 | 6.783E-03 | CLTCL1 | clathrin heavy chain like 1 | 1.4830 | 3.476E-02 |
| GPER1 | G protein-coupled estrogen receptor 1 | 1.9151 | 2.402E-02 | CCR2 | C-C motif chemokine receptor 2 | 1.4782 | 3.913E-02 |
| MMP25 | matrix metallopeptidase 25 | 1.8759 | 1.785E-02 | LGALS3 | galectin 3 | 1.4766 | 2.499E-02 |
| APCDD1 | APC down-regulated 1 | 1.8585 | 2.483E-02 | USP46 | ubiquitin specific peptidase 46 | 1.4742 | 2.442E-02 |
| HP | haptoglobin | 1.8489 | 3.124E-02 | C1orf216 | chromosome 1 open reading frame 216 | 1.4675 | 2.374E-03 |
| RAB6B | member RAS oncogene family | 1.8489 | 1.496E-02 | RRP12 | ribosomal RNA processing 12 homolog | 1.4669 | 3.146E-02 |
| MCEMP1 | mast cell expressed membrane protein 1 | 1.8387 | 2.442E-02 | PLA2G15 | phospholipase A2 group XV | 1.4585 | 1.482E-02 |
| RP11-89M16.1 | N/A | 1.8355 | 3.669E-02 | CDYL2 | chromodomain Y like 2 | 1.4552 | 6.803E-03 |
| ELANE | elastase, neutrophil expressed | 1.8274 | 3.649E-02 | GAPDH | glyceraldehyde-3-phosphate dehydrogenase | 1.4470 | 4.157E-03 |
| DHCR24 | matrix metallopeptidase 9 | 1.8111 | 8.93E-05 | PGD | phosphogluconate dehydrogenase | 1.4461 | 4.032E-03 |
| MMP9 | 24-dehydrocholesterol reductase | 1.8103 | 4.495E-02 | SQLE | squalene epoxidase | 1.4396 | 3.728E-02 |
| HBA2 | hemoglobin subunit alpha 2 | 1.8059 | 2.747E-02 | PTGER2 | prostaglandin E receptor 2 | 1.4389 | 4.157E-03 |
| HBA1 | hemoglobin subunit alpha 1 | 1.8058 | 2.896E-02 | ANXA2 | annexin A2 | 1.4388 | 1.208E-03 |
| TMEM200A | transmembrane protein 200A | 1.8028 | 2.994E-02 | STOM | stomatin | 1.4378 | 3.941E-02 |
| VSIG10L | V-set and immunoglobulin domain containing 10 like | 1.7636 | 3.803E-02 | LGALS1 | galectin 1 | 1.4328 | 3.649E-02 |
| GABRR2 | gamma-aminobutyric acid type A receptor rho2 subunit | 1.7634 | 2.352E-02 | SLC36A1 | solute carrier family 36 member 1 | 1.4122 | 2.753E-02 |
| MIAT | myocardial infarction associated transcript (non-protein coding) | 1.7567 | 3.051E-03 | NATD1 | N-acetyltransferase domain containing 1 | 1.4063 | 4.368E-02 |
| HSF4 | heat shock transcription factor 4 | 1.7554 | 6.863E-03 | LINC00341 (SYNE3) | long intergenic non-protein coding RNA 341 | 1.3988 | 1.024E-02 |
| RP11-229P13.19 | N/A | 1.7366 | 4.704E-02 | IL17RA | interleukin 17 receptor A | 1.3988 | 6.384E-04 |
| RP11-545E17.3 | N/A | 1.7321 | 2.994E-02 | SIRPA | signal regulatory protein alpha | 1.3979 | 1.496E-02 |
| E2F2 | E2F transcription factor 2 | 1.7199 | 1.208E-03 | CDC42EP3 | CDC42 effector protein 3 | 1.3925 | 4.704E-02 |
| HRH2 | histamine receptor H2 | 1.7134 | 3.828E-02 | LDHA | lactate dehydrogenase A | 1.3902 | 1.714E-02 |
| CPT1A | carnitine palmitoyltransferase 1A | 1.6824 | 4.178E-03 | PLIN3 | perilipin 3 | 1.3790 | 2.65E-02 |
| GGT1 | tumor protein p63 regulated 1 | 1.6811 | 6.78E-05 | CYFIP1 | cytoplasmic FMR1 interacting protein 1 | 1.3745 | 2.747E-02 |
| **Supplementary Table 9.** Continued | | | | | | | |
| TPRG1 | gamma-glutamyltransferase 1 | 1.6810 | 8.185E-03 | CD84 | CD84 molecule | 1.3675 | 8.446E-03 |
| ABCC4 | ATP binding cassette subfamily C member 4 | 1.6744 | 4.333E-02 | ITGB1 | integrin subunit beta 1 | 1.3606 | 2.352E-02 |
| CDKN2B | cyclin dependent kinase inhibitor 2B | 1.6682 | 4.887E-02 | VDR | vitamin D receptor | 1.3572 | 4.887E-02 |
| C9orf47 | chromosome 9 open reading frame 47 | 1.6616 | 4.037E-02 | CAPG | capping actin protein, gelsolin like | 1.3504 | 4.884E-02 |
| MMP14 | matrix metallopeptidase 14 | 1.6497 | 2.753E-02 | TMEM173 | transmembrane protein 173 | 1.3476 | 1.766E-02 |
| GAS6 | growth arrest specific 6 | 1.6496 | 6.794E-03 | DPYSL2 | dihydropyrimidinase like 2 | 1.3348 | 2.606E-02 |
| CD36 | CD36 molecule | 1.6482 | 4.677E-03 | PLEK | pleckstrin | 1.3256 | 2.442E-02 |
| RNASE2 | ribonuclease A family member 2 | 1.6473 | 3.217E-02 | RCBTB2 | RCC1 and BTB domain containing protein 2 | 1.3116 | 4.164E-02 |
| MMRN1 | multimerin 1 | 1.6375 | 2.753E-02 | SYNE3 | spectrin repeat containing nuclear envelope family member 3 | 1.3080 | 3.601E-02 |
| LINC00426 | long intergenic non-protein coding RNA 426 | 1.6332 | 8.16E-03 | C1RL | complement C1r subcomponent like | 1.3078 | 3.568E-02 |
| FAM129B | family with sequence similarity 129 member B | 1.6316 | 7.246E-03 | MYO1G | myosin IG | 1.3026 | 3.942E-02 |
| EVC2 | EvC ciliary complex subunit 2 | 1.6255 | 2.345E-02 | ALAS1 | 5'-aminolevulinate synthase 1 | 1.2966 | 3.588E-02 |
| PLBD1 | phospholipase B domain containing 1 | 1.6206 | 4.552E-02 | SREBF2 | sterol regulatory element binding transcription factor 2 | 1.2886 | 1.714E-02 |
| MARC1 | mitochondrial amidoxime reducing component 1 | 1.6117 | 1.882E-03 | MAP4 | microtubule associated protein 4 | 1.2857 | 1.141E-02 |
| FAM129A | family with sequence similarity 129 member A | 1.5991 | 2.78E-08 | PIAS3 | protein inhibitor of activated STAT 3 | 1.2764 | 2.345E-02 |
| ASS1P1 | argininosuccinate synthetase 1 pseudogene 1 | 1.5917 | 1.024E-02 | GYG1 | glycogenin 1 | 1.2610 | 4.704E-02 |
| IL2RA | interleukin 2 receptor subunit alpha | 1.5824 | 1.012E-02 | PPP4R1 | protein phosphatase 4 regulatory subunit 1 | 1.2486 | 2.999E-02 |
| PTGFRN | prostaglandin F2 receptor inhibitor | 1.5727 | 3.864E-02 | MAPK14 | mitogen-activated protein kinase 14 | 1.2469 | 3.568E-02 |
| RP11-147I3.1 | N/A | 1.5711 | 4.322E-02 | CASP2 | caspase 2 | 1.2319 | 4.037E-02 |
| IMPA2 | inositol monophosphatase 2 | 1.5658 | 2.402E-02 | ATXN1 | ataxin 1 | 1.2284 | 4.156E-02 |
| AP1S1 | adaptor related protein complex 1 sigma 1 subunit | 1.5638 | 6.863E-03 | CFL1 | cofilin 1 | 1.2269 | 4.543E-02 |
| FKBP5 | FK506 binding protein 5 | 1.5439 | 3.649E-02 | WWP2 | WW domain containing E3 ubiquitin protein ligase 2 | 1.2211 | 3.211E-02 |
| IGSF9B | immunoglobulin superfamily member 9B | 1.5435 | 4.543E-02 | CDS2 | CDP-diacylglycerol synthase 2 | 1.2174 | 3.018E-04 |
| TPM4 | tropomyosin 4 | 1.5240 | 1.714E-02 | CTDSP1 | CTD small phosphatase 1 | 1.2048 | 2.753E-02 |

**Supplementary Table 10.** 113 downregulated genes resulted from DESeq2 differential expression analysis with *P* < 0.05 in patients with LEAD, in comparison healthy controls. Genes were ordered according to increasing Fold change value.

| **Gene symbol** | **Gene name** | **Fold change** | ***P* value** | **Gene symbol** | **Gene name** | **Fold change** | ***P* value** |
| --- | --- | --- | --- | --- | --- | --- | --- |
| SLC4A10 | solute carrier family 4 member 10 | 0.2448 | 7.094E-18 | PMEPA1 | prostate transmembrane protein, androgen induced 1 | 0.6125 | 2.747E-02 |
| NRCAM | neuronal cell adhesion molecule | 0.3024 | 2.730E-09 | PDE4B | phosphodiesterase 4B | 0.6128 | 1.115E-02 |
| CD248 | CD248 molecule | 0.3241 | 7.494E-08 | WHRN | whirlin | 0.6129 | 3.211E-02 |
| NOG | noggin | 0.3390 | 2.025E-07 | MS4A1 | membrane spanning 4-domains A1 | 0.6147 | 2.148E-02 |
| B3GNT7 | UDP-GlcNAc:betaGal beta-1,3-N-acetylglucosaminyltransferase 7 | 0.3863 | 7.621E-08 | FCRLA | Fc receptor like A | 0.6157 | 4.187E-02 |
| FBLN2 | fibulin 2 | 0.3928 | 5.539E-06 | CCDC171 | coiled-coil domain containing 171 | 0.6172 | 2.572E-02 |
| ADGRA3 | adhesion G protein-coupled receptor A3 | 0.4148 | 1.409E-06 | ZNF331 | zinc finger protein 331 | 0.6235 | 9.673E-05 |
| MMP28 | matrix metallopeptidase 28 | 0.4705 | 4.157E-03 | IL18RAP | interleukin 18 receptor accessory protein | 0.6237 | 3.601E-02 |
| SLC16A10 | solute carrier family 16 member 10 | 0.4752 | 6.168E-05 | PLXDC1 | plexin domain containing 1 | 0.6286 | 4.552E-02 |
| GAL3ST4 | galactose-3-O-sulfotransferase 4 | 0.4881 | 2.396E-04 | AMZ2P1 | archaelysin family metallopeptidase 2 pseudogene 1 | 0.6294 | 4.157E-03 |
| ZNF667-AS1 | ZNF667 antisense RNA 1 (head to head) lncRNA | 0.4952 | 5.774E-03 | OBSCN | obscurin, cytoskeletal calmodulin and titin-interacting RhoGEF | 0.6303 | 8.006E-03 |
| RP11-641A6.2 | N/A | 0.4953 | 7.844E-03 | EPPK1 | epiplakin 1 | 0.6368 | 2.753E-02 |
| SOBP | sine oculis binding protein homolog | 0.4998 | 7.844E-03 | KLRB1 | killer cell lectin like receptor B1 | 0.6399 | 4.691E-02 |
| SIK1 | salt inducible kinase 1 | 0.5008 | 2.468E-03 | RETREG1 | reticulophagy regulator 1 | 0.6450 | 3.211E-02 |
| DBNDD1 | dysbindin domain containing 1 | 0.5080 | 6.393E-03 | ZNF439 | zinc finger protein 439 | 0.6472 | 3.040E-03 |
| TTC24 | tetratricopeptide repeat domain 24 | 0.5086 | 1.049E-03 | SDK2 | sidekick cell adhesion molecule 2 | 0.6474 | 4.543E-02 |
| AK5 | adenylate kinase 5 | 0.5112 | 1.569E-05 | IL18R1 | interleukin 18 receptor 1 | 0.6487 | 1.115E-02 |
| OLMALINC | oligodendrocyte maturation-associated long intergenic non-coding RNA | 0.5166 | 1.024E-02 | TRABD2A | TraB domain containing 2A | 0.6490 | 4.704E-02 |
| EDAR | ectodysplasin A receptor | 0.5194 | 1.496E-02 | ZNF814 | zinc finger protein 814 | 0.6539 | 1.923E-02 |
| ZNF154 | zinc finger protein 154 | 0.5195 | 2.468E-03 | AUTS2 | AUTS2, activator of transcription and developmental regulator | 0.6563 | 1.024E-02 |
| CCR12P | C-C motif chemokine receptor 12, pseudogene | 0.5238 | 2.442E-02 | RP11-796E2.4 | N/A | 0.6567 | 2.542E-02 |
| NKD1 | naked cuticle homolog 1 | 0.5254 | 4.178E-03 | CD7 | CD7 molecule | 0.6585 | 4.240E-03 |
| SLC7A8 | solute carrier family 7 member 8 | 0.5256 | 2.542E-02 | KLHL3 | kelch like family member 3 | 0.6588 | 6.803E-03 |
| LINC01088 | long intergenic non-protein coding RNA 1088 | 0.5302 | 1.714E-02 | DHRS3 | dehydrogenase/reductase 3 | 0.6617 | 1.496E-02 |
| IL31RA | interleukin 31 receptor A | 0.5309 | 2.650E-02 | RALGPS2 | Ral GEF with PH domain and SH3 binding motif 2 | 0.6625 | 4.339E-02 |
| RP11-158G18.1 | N/A | 0.5314 | 4.167E-03 | LTBP3 | latent transforming growth factor beta binding protein 3 | 0.6635 | 2.071E-02 |
| PDE9A | phosphodiesterase 9A | 0.5381 | 1.175E-02 | FCMR | Fc fragment of IgM receptor | 0.6642 | 1.141E-02 |
| ZNF542P | zinc finger protein 542, pseudogene | 0.5405 | 2.125E-04 | GPA33 | glycoprotein A33 | 0.6680 | 4.357E-02 |
| **Supplementary Table 10.** Continued | | | | | | | |
| CCR9 | C-C motif chemokine receptor 9 | 0.5465 | 2.442E-02 | LINC01550 | long intergenic non-protein coding RNA 1550 | 0.6704 | 2.753E-02 |
| AEBP1 | AE binding protein 1 | 0.5542 | 2.571E-03 | ZNF347 | zinc finger protein 347 | 0.6730 | 3.019E-02 |
| ABCG1 | ATP binding cassette subfamily G member 1 | 0.5546 | 4.330E-05 | BRD7P2 | bromodomain containing 7 pseudogene 2 | 0.6731 | 1.088E-02 |
| PTPRK | protein tyrosine phosphatase, receptor type K | 0.5571 | 8.185E-03 | MAN1C1 | mannosidase alpha class 1C member 1 | 0.6763 | 1.227E-03 |
| RP11-136K14.1 | N/A | 0.5572 | 4.704E-02 | GPRASP1 | G protein-coupled receptor associated sorting protein 1 | 0.6763 | 2.442E-02 |
| NR4A2 | nuclear receptor subfamily 4 group A member 2 | 0.5600 | 1.482E-02 | TTN | titin | 0.6897 | 4.505E-02 |
| LINC00865 | long intergenic non-protein coding RNA 865 | 0.5609 | 2.747E-02 | PDE3B | phosphodiesterase 3B | 0.6936 | 6.863E-03 |
| GRAPL | GRB2 related adaptor protein like | 0.5663 | 1.496E-02 | BCL11A | B-cell CLL/lymphoma 11A | 0.6971 | 2.492E-02 |
| SPIB | Spi-B transcription factor | 0.5677 | 4.240E-03 | TCF4 | transcription factor 4 | 0.7006 | 3.211E-02 |
| CTD-3018O17.3 | N/A | 0.5695 | 4.837E-02 | CTD-2368P22.1 | N/A | 0.7036 | 4.884E-02 |
| AMN | amnion associated transmembrane protein | 0.5716 | 3.030E-02 | SNORD116-20 | small nucleolar RNA, C/D box 116-20 | 0.7099 | 4.959E-02 |
| FCGBP | Fc fragment of IgG binding protein | 0.5733 | 3.154E-03 | SATB1 | SATB homeobox 1 | 0.7156 | 3.568E-02 |
| ZSCAN18 | zinc finger and SCAN domain containing 18 | 0.5774 | 1.409E-06 | PDE7A | phosphodiesterase 7A | 0.7184 | 3.869E-04 |
| CR2 | complement C3d receptor 2 | 0.5804 | 3.568E-02 | FLNB | filamin B | 0.7185 | 2.747E-02 |
| CHRM3-AS2 | CHRM3 antisense RNA 2, lncRNA | 0.5809 | 3.750E-02 | PRAG1 | PEAK1 related, kinase-activating pseudokinase 1 | 0.7227 | 3.154E-03 |
| LTK | leukocyte receptor tyrosine kinase | 0.5824 | 3.967E-02 | PTMAP2 | prothymosin, alpha pseudogene 2 | 0.7305 | 4.543E-02 |
| IGHM | immunoglobulin heavy constant mu | 0.5848 | 3.080E-02 | GRAP | GRB2-related adaptor protein | 0.7309 | 8.325E-03 |
| TMIGD2 | transmembrane and immunoglobulin domain containing 2 | 0.5853 | 3.512E-02 | HIP1R | huntingtin interacting protein 1 related | 0.7439 | 1.175E-02 |
| ADAM12 | ADAM metallopeptidase domain 12 | 0.5853 | 3.601E-02 | NAA16 | N(alpha)-acetyltransferase 16, NatA auxiliary subunit | 0.7490 | 8.325E-03 |
| OBSCN-AS1 | OBSCN antisense RNA 1, lncRNA | 0.5863 | 3.772E-02 | PITPNC1 | phosphatidylinositol transfer protein, cytoplasmic 1 | 0.7585 | 1.482E-02 |
| KLHL29 | kelch like family member 29 | 0.5917 | 3.601E-02 | OPN3 | opsin 3 | 0.7588 | 2.442E-02 |
| CLEC17A | C-type lectin domain containing 17A | 0.5961 | 7.571E-03 | SNORA72 | small nucleolar RNA, H/ACA box 72 | 0.7611 | 1.505E-02 |
| GPR18 | G protein-coupled receptor 18 | 0.5965 | 1.208E-03 | FOXO1 | forkhead box O1 | 0.7660 | 5.497E-03 |
| TRBV2 | T-cell receptor beta variable 2 | 0.6003 | 3.110E-02 | ATM | ATM serine/threonine kinase | 0.7685 | 4.660E-03 |
| PLAG1 | PLAG1 zinc finger | 0.6004 | 7.246E-03 | SLC12A2 | solute carrier family 12 member 2 | 0.7812 | 1.173E-02 |
| BACH2 | BTB domain and CNC homolog 2 | 0.6051 | 1.128E-03 | RING1 | ring finger protein 1 | 0.8017 | 2.753E-02 |
| STAP1 | signal transducing adaptor family member 1 | 0.6099 | 4.458E-02 | FOXP1 | zinc finger protein 439 | 0.8055 | 4.543E-02 |
| COBLL1 | cordon-bleu WH2 repeat protein like 1 | 0.6102 | 2.243E-02 | TIA1 | small nucleolar RNA, C/D box 116-20 | 0.8079 | 2.573E-02 |
| CREM | cAMP responsive element modulator | 0.6121 | 4.444E-03 |  |  |  |  |

**Supplementary Table 11.** Results of ROC analysis of 14 selected genes.

| **Gene** | **ROC-AUC^1^** | **Threshold** | **Specificity** | **Sensitivity** | **Accuracy** | **Positive Predictive Value** | **Negative Predictive Value** |
| --- | --- | --- | --- | --- | --- | --- | --- |
| *AK5* | 1.000 | 6.134 | 1.000 | 1.000 | 1.000 | 1.000 | 1.000 |
| *CD248* | 1.000 | 3.988 | 1.000 | 1.000 | 1.000 | 1.000 | 1.000 |
| *FAM129A* | 1.000 | 9.758 | 1.000 | 1.000 | 1.000 | 1.000 | 1.000 |
| *FBLN2* | 1.000 | 4.562 | 1.000 | 1.000 | 1.000 | 1.000 | 1.000 |
| *GGT1* | 1.000 | 6.420 | 1.000 | 1.000 | 1.000 | 1.000 | 1.000 |
| *NOG* | 1.000 | 4.508 | 1.000 | 1.000 | 1.000 | 1.000 | 1.000 |
| *NRCAM* | 1.000 | 3.725 | 1.000 | 1.000 | 1.000 | 1.000 | 1.000 |
| *RP11-545E17.3* | 1.000 | 3.521 | 1.000 | 1.000 | 1.000 | 1.000 | 1.000 |
| *SLC12A2* | 1.000 | 7.379 | 1.000 | 1.000 | 1.000 | 1.000 | 1.000 |
| *SLC4A10* | 1.000 | 5.029 | 1.000 | 1.000 | 1.000 | 1.000 | 1.000 |
| *ZSCAN18* | 1.000 | 7.161 | 1.000 | 1.000 | 1.000 | 1.000 | 1.000 |
| *CDS2* | 0.982 | 10.019 | 0.875 | 1.000 | 0.933 | 0.875 | 1.000 |
| *SLC16A10* | 0.982 | 4.954 | 0.875 | 1.000 | 0.933 | 0.875 | 1.000 |
| *PDE7A* | 0.964 | 9.666 | 1.000 | 0.857 | 0.933 | 1.000 | 0.889 |
| ^1^ Area under ROC curve. | | | | |  |  |  |

**Supplementary Table 12:** Experimentally validated interactions between 26 miRNAs and 14 genes indicative for LEAD.

| **Database** | **miRNA ID** | **Gene symbol** | **Method** |  |
| --- | --- | --- | --- | --- |
| mirecords | hsa-miR-21-5p | *SLC16A10* | - |  |
| mirtarbase | hsa-miR-548t-3p | *FBLN2* | PAR-CLIP^1^ |  |
|  | hsa-miR-548aa | *FBLN2* | PAR-CLIP^1^ |  |
|  | hsa-miR-21-5p | *SLC16A10* | Microarray |  |
|  | hsa-miR-122-5p | *SLC16A10* | HITS-CLIP^2^ |  |
|  | hsa-miR-15a-5p | *CDS2* | HITS-CLIP^2^ |  |
|  | hsa-miR-766-3p | *FAM129A* | HITS-CLIP^2^ |  |
| tarbase | hsa-miR-21-5p | *SLC16A10* | Microarray |  |
| ^1^Photoactivatable Ribonucleoside-Enhanced Crosslinking and Immunoprecipitation | | | | |
| ^2^High-Throughput Sequencing of RNA isolated by Cross-Linking Immunoprecipitation | | | | |

**Supplementary Table 13:** Top 10% predicted interactions between 26 miRNAs and 14 genes indicative for LEAD.

| **Database** | **miRNA ID** | **Gene symbol** | **Database-specific probability value** |
| --- | --- | --- | --- |
| diana_microt | hsa-miR-125b-5p | *SLC4A10* | 0.994 |
|  | hsa-miR-3591-3p | *SLC4A10* | 0.988 |
|  | hsa-miR-30e-3p | *SLC12A2* | 0.961 |
|  | hsa-miR-15a-5p | *SLC12A2* | 0.951 |
|  | hsa-miR-548d-5p | *NRCAM* | 0.933 |
|  | hsa-miR-548t-3p | *NRCAM* | 0.916 |
|  | hsa-miR-548aa | *NRCAM* | 0.916 |
|  | hsa-miR-548t-3p | *CDS2* | 0.916 |
|  | hsa-miR-548aa | *CDS2* | 0.916 |
|  | hsa-miR-21-5p | *SLC16A10* | 0.912 |
|  | hsa-miR-15a-5p | *SLC4A10* | 0.894 |
|  | hsa-miR-30e-3p | *NRCAM* | 0.878 |
|  | hsa-miR-766-3p | *SLC4A10* | 0.873 |
|  | hsa-miR-330-3p | *SLC4A10* | 0.852 |
|  | hsa-miR-34a-3p | *SLC4A10* | 0.845 |
|  | hsa-miR-330-3p | *SLC12A2* | 0.843 |
|  | hsa-miR-125b-5p | *CDS2* | 0.835 |
|  | hsa-miR-34a-3p | *SLC12A2* | 0.822 |
|  | hsa-miR-548d-5p | *SLC12A2* | 0.822 |
|  | hsa-miR-30e-3p | *SLC16A10* | 0.818 |
|  | hsa-miR-330-3p | *CDS2* | 0.816 |
|  | hsa-miR-548d-5p | *SLC16A10* | 0.807 |
|  | hsa-miR-548t-3p | *FBLN2* | 0.805 |
|  | hsa-miR-548aa | *FBLN2* | 0.805 |
| elmmo | hsa-miR-125b-5p | *SLC4A10* | 0.78 |
|  | hsa-miR-15a-5p | *SLC12A2* | 0.767 |
|  | hsa-miR-125b-5p | *PDE7A* | 0.75 |
|  | hsa-miR-15a-5p | *CDS2* | 0.734 |
|  | hsa-miR-15a-5p | *SLC12A2* | 0.73 |
|  | hsa-miR-330-3p | *PDE7A* | 0.672 |
|  | hsa-miR-330-3p | *SLC12A2* | 0.657 |
|  | hsa-miR-330-3p | *SLC4A10* | 0.645 |
|  | hsa-miR-330-3p | *NRCAM* | 0.622 |
|  | hsa-miR-34a-5p | *SLC12A2* | 0.612 |
|  | hsa-miR-548d-5p | *SLC12A2* | 0.57 |
|  | hsa-let-7f-1-3p | *AK5* | 0.509 |
|  | hsa-let-7f-1-3p | *PDE7A* | 0.509 |
| microcosm | hsa-miR-34b-5p | *PDE7A* | 18.2142 |
|  | | | |
|  | | | |
| **Supplementary Table 13.** Continued | | | |
| miranda | hsa-miR-125b-5p | *PDE7A* | -0.99 |
|  | hsa-miR-138-5p | *ZSCAN18* | -0.9969 |
|  | hsa-miR-21-5p | *SLC16A10* | -1.1057 |
|  | hsa-miR-330-3p | *SLC4A10* | -1.1536 |
|  | hsa-miR-99a-3p | *FAM129A* | -1.1648 |
| mirdb | hsa-miR-15a-5p | *SLC12A2* | 96.055 |
|  | hsa-miR-330-3p | *SLC4A10* | 93.776 |
|  | hsa-miR-330-3p | *SLC4A10* | 93.775 |
|  | hsa-miR-125b-5p | *PDE7A* | 92.548 |
| pictar | hsa-miR-15a-5p | *CDS2* | 45.942 |
| pita | hsa-miR-34a-5p | *CDS2* | -10.96 |
|  | hsa-miR-34a-5p | *PDE7A* | -11.08 |
|  | hsa-miR-423-3p | *FAM129A* | -11.7 |
|  | hsa-miR-548d-5p | *NRCAM* | -12.08 |
|  | hsa-miR-766-3p | *FAM129A* | -12.64 |
|  | hsa-miR-34a-5p | *PDE7A* | -14.78 |
|  | hsa-miR-34a-5p | *SLC12A2* | -15.56 |
|  | hsa-miR-34a-5p | *PDE7A* | -18 |
|  | hsa-miR-138-5p | *PDE7A* | -18.79 |
|  | hsa-miR-423-3p | *CDS2* | -18.9 |
| targetscan | hsa-miR-1301-3p | *SLC16A10* | -0.25 |
|  | hsa-miR-125b-5p | *SLC4A10* | -0.26 |
|  | hsa-miR-99a-3p | *PDE7A* | -0.271 |
|  | hsa-miR-21-5p | *SLC16A10* | -0.336 |
|  | hsa-miR-99a-3p | *FAM129A* | -0.361 |
|  | hsa-miR-125b-5p | *PDE7A* | -0.372 |
|  | hsa-miR-6087 | *NOG* | -0.427 |
|  | hsa-miR-138-5p | *ZSCAN18* | -0.469 |

| **Supplementary Table 14.** Comparison of methodology reported in literature with methodology applied in current study by Bogucka-Kocka et al. | | | | | | | | | |
| --- | --- | --- | --- | --- | --- | --- | --- | --- | --- |
| **Reported experiment** | **Biological material** | **Disease/Condition** | **Number of samples** | **Number of controls** | **Method of experiment** | **Number of microRNA/gene expression analyzed** | **Verification** | **miRNA/gene findings from reported experiments** | **Comments** |
| Bogucka-Kocka et al (current study) | PBMCs | LEAD | 40 patients (microRNA), 8 patients (transcriptome) | 19 healthy controls (microRNA), 7 healthy controls (transcriptome) | NGS, ion torrent | ca. 3000 microRNA,  ca. 55000 genes and splicing variants | boardened strict statistical analysis: p<0.0001, UVE-PLS, ROC; evaluation of miRNA targets by transcriptome sequencing | ***let-7f-1-3p ↑***,  miR-34a-5p,  ***-122-5p ↑***,  -3591-3p,  -***34a-3p* ↑**,  -1261,  -***21-5p ↑***,  -15a-5p,  -548d-5p,  -34b-5p,  -424-3p,  -548aa,  -548t-3p,  -4423-3p,  -196a-5p,  -330-3p,  -766-3p,  ***-30e-3p ↓***,  ***-125b-5p ↓***,  -1301-3p,  -3184-5p,  ***-423-3p ↓***,  -339-3p,  -138-5p,  -99a-3p  -6087  AK5, CD248,  CDS2, FAM129A, FBLN2, GGT1,  NOG, NRCAM, PDE7A, RP11-545E17.3,  SLC12A2, SLC16A10,  SLC4A10  ZSCAN18 | current study |
| Al-Kafaji 2016 | peripheral blood | CAD patients | 45 patients,  45 type 2 diabetes patients | 45 healthy controls | qRT-PCR | 1 microRNA | area under the ROC curves | miR 126 | low count of microRNA analyzed |
| Cippolone 2011 | atherosclerotic plaques | symptomatic and asymptomatic plaques patients | symptomatic plaques(n=22), asymptomatic plaques (n=31) | symptomatic vs asymptomatic plaques | TaqMan miRNA assays | 41 microRNAs | RT-qPCR of 5 miRNAs:  miRNA-100, miRNA-127, miRNA-145, miRNA-133a, miRNA-133b in independent 38 plaques specimens | miRNA-100, miRNA-127, miRNA-145, miRNA-133a, miRNA-133b | direct comparsion difficult- different experiment design (controls), low count of microRNA analyzed |
| D’Alessandra 2013 | platelet depleted plasma | CAD patients with stable and unstable angina | 53 patients,  **4 taken for microarray card analysis** | 20 healthy controls, **4 taken for microarray card analysis** | TaqMan Human microRNA Card A Arrays version 2.0 | 367 microRNAs | RT-PCR of 3 microRNAs in cohorts of SAP, UAP patients and controls (10 samples from each cohort), literature miRs validation | miR-337-5p,  miR-433,  miR-485-3p  literature miRs validation:  miR-1, miR-126, miR-485-3p (SAP);  miR-1, miR-126, miR133a (UAP) | direct comparsion difficult- different sample count |
| Dolz 2017 | plasmal exosomes | ACAS | 10 patients with ACAS progression | 6 patients without ACAS progression | microarray Affymetrix GeneChip miRNA 4.0 | 6600 human noncoding RNA probes, including 2578 mature miRNAs, 2025 premiRNAs | qRT-PCR of 14 micro RNA in 39 ACAS patients | miR-199b-3p,  miR-130a-3p,  miR-146a-5p,  miR-221-3p,  miR-361-5p,  miR-26a-5p,  miR-27b-3p,  miR-103a-3p,  miR-151a-5p,  miR-199a-5p,  miR-222-3p,  miR-23b-3p,  miR-24-3p | direct comparsion difficult- different experiment design (controls) |
| Dong 2017 | PBMCs | CAD patients | 161 stable CAD patients | 149 health controls | qRT-PCR | 7 micro RNAs | area under the ROC curves | miR-24  miR-33a  miR-103a  ***miR-122*** ↑ | low count of microRNA analyzed |
| Fichtlscherer 2010 | plasma | CAD | 8 patients | 8 healthy controls | Geniom Biochip MPEA Homo sapiens (Febit biomed GmbH, Heidelberg, Germany) | no data | qRT-PCR of 8 selected micro RNAs in 31 CAD patients and 14 healthy controls | hsa-miR-126  hsa-miR-17  hsa-miR-20a  hsa-miR-92a  hsa-miR-221  hsa-miR-199a-5p  hsa-miR-27a  hsa-miR-130a  hsa-let-7d  ***hsa-miR-21 ↓***  hsa-miR-1  hsa-miR-133a  hsa-miR-133b  hsa-miR-208b  hsa-miR-208a  hsa-miR-499–3p  hsa-miR-499–5p  hsa-miR-143  hsa-miR-145 | direct comparsion difficult- different samples count |
| Han 2015 | plasma of humans (52 samples) and mice (9 samples) | CAD | 32 patients | 20 healthy controls | Taqman microRNA assays (humans), miRCURY LNA Array v.16.0 (Exiqon) (mice) | 752 murine miRNAs | PCR of human mmu miRs counterparts in CAD and healthy controls | ***mmu miR-34a ↑,***  ***mmu miR-21 ↑,***  mmu miR-23a,  mmu miR-30a,  mmu miR-106b | direct comparsion difficult- murine microRNA in initial studies |
| Hoekstra 2010 | PMBCs | CAD patients | 25 unstable angina pectoris, 25 stable angina pectoris cases  **RNA pooled into 6 samples** | 20 healthy controls  **RNA pooled into 3 samples** | TaqMan miRNA assays | initially 157 microRNAs | CAD both groups vs control,  SAP vs UAP | CAD (both groups) vs control mir-135a,  miR-147  SAP vs UAP  miR-134,  miR-370,  miR-198 | direct comparsion difficult- pooled samples, low count of microRNA analyzed |
| Huang 2014 | plasma | AMI Acute myocardial Infraction patients | 20 patients  **pooled plasma**  validation I: 178 patients  validation II 198 patients | 20 controls  **pooled plasma** | NGS, Illumina Solexa sequencer, qRT-PCR of 2 micrRNAs | whole transcriptome, subsequent analysis of 2 microRNAs | case–control  studies validation:  I (178 AMI vs 198 controls)  II (150 AMI vs 150 controls)  with   \| miR-320b \| \| --- \| \| miR-125b \| | \| ***miR-125b ↓*** \| \| --- \| \| miR-320b \| \| miR-25 \| \| miR-483-5p \| \| miR-106b \|   (vs *Caenorhabditis elegans* mir-39) | direct comparsion difficult- pooled samples, microRNA analyzed |
| Jiang 2014 | peripheral blood | atherosclerotic patients | 20 atherosclerotic patients, 20 patients with atherosclerosis indicators (preatherosclerotic condition) | 20 healthy controls | qRT-PCR MirCountTM system (Chi Biotechnology, Jiangyin, China) | 8 microRNAs: | p < 0.01 Athero vs control, pre-Athero vs control, and Athero vs pre-Athero | ***miR-21 ↑***  miR-92a  ***miR-122 ↑***  miR-126  miR-130a  miR-211  miR-222  miR-370 | direct comparsion difficult- different count of microRNA analyzed |
| Karakas 2017 | peripheral blood | CAD patients: SAP, ACS | 1112 patients | SAP vs ACS | qRT-PCR | 8 miRNAs: | SAP vs ACS | miR-19a,  miR-19b,  miR-132,  miR-140-3p,  miR-142-5p,  miR-150,  miR-186,  miR-210 | direct comparsion difficult- different experiment design (controls), different count of microRNA analyzed |
| Leistner 2016 | plasma | CAD patients | 52 samples | correlations of miRNA levels with coronary atherosclerotic plaque characteristics | TaqMan miRNA assays | 13 microRNAs | area under the ROC curves | miR-29b-3p  miR-126-5p  miR-145-5p  miR-155-5p | direct comparsion difficult- different experiment design, low count of microRNA analyzed (controls) |
| Li 2010 | peripheral blood (serum),  sclerotic intima,  normal adjacent intima | ASO/PAD patients | 51 sclerotic intima and normal intima samples | sclerotic intima vs normal intima | Sybr green RT-PCR | 13 microRNAs in intima samples from 51 ASO patients. | RT-PCR of 7 miRs from serum of 104 ASO/PAD patients and 105 healthy controls | ***miR-21 ↑***,  miR-130a,  miR-27b,  ***let-7f ↑***  miR-210  miR-221  miR-222  sclerotic samples vs normal samples  104 ASO vs 105 controls  miR-130a,  miR-27b  miR-210 | direct comparsion difficult- different experiment design (controls), low count of microRNA analyzed |
| Patino 2005 | monocytes from blood,  macrophages from carotid artery plaques | CAD patients | monocyte libraries from **2 patients, 2 younger patients**, | **1 healthy control** | LongSAGE protocol for SpectruMedix 192-capillary automated sequencer | whole transcriptome (mRNA) | qRT-PCR in 25 CAD patients and 19 controls of 6 candidate genes, FOS expression in monocytes and plaque macrophages | FOS  DUSP1  NFKBIA  ID2  PER1  SAP30 | direct comparsion difficult- different samples count |
| Raitoharju 2011 | arterial endarterectomy samples | atherosclerosis patients | 16 samples microarray,  18 samples Genome-wide expression analysis | non-atherosclerotic left internal thoracic arteries (LITA)  **6 for microarray**,  20 for qRT-PCR, **20 for sequencing** | Human miRNA Microarray Version 3 (Agilent), containing 866 human and 89 human viral miRNAs, Illumina HumanHT-12 v3 Expression Bead-Chip | 955 microRNAs | PCR of all 50 samples (plaques + controls) of 6 microRNAs | ***miR-21 ↑***  ***miR-34 ↑***  miR-146a  miR-146b-5p  miR-210 | direct comparsion difficult- different experiment design (controls) |
| Ren 2013 | plasma | CAD patients | 13 patients with Unstable Angina Pectoris (UAP) | 13 patients with chest pain or distress attributable to non-cardiac  causes | Taqman low-density miRNA array. Human MicroRNA TLDA card A+B version 3.0 (Applied Biosystems) | 754 mature miRNAs | RT-qPCR of 7 selected miRNAs  miR-106b  miR-25  miR-92a  miR-21  miR-590-5p  miR-126*  miR-451  in independent cohorts of:  45 UAP patients  31 SAP patients  37 controls | hsa-miR-106b  hsa-miR-25  hsa-miR-93  hsa-miR-17  hsa-miR-19b  hsa-miR-20a  hsa-miR-92a  ***hsa-miR-21 ↑***  hsa-miR-590-5p  hsa-miR-106a  hsa-miR-20b  hsa-miR-16  hsa-miR-195  hsa-miR-26a  hsa-miR-26b  hsa-miR-30a-5p  hsa-miR-30b  hsa-miR-30c  hsa-miR-30d  hsa-miR-126*  hsa-miR-1274b  hsa-miR-140-5p  hsa-miR-142-3p  hsa-miR-146a  hsa-miR-185  hsa-miR-192  hsa-miR-223  hsa-miR-27a  hsa-miR-29a  hsa-miR-328  hsa-miR-374a  hsa-miR-375  hsa-miR-425  hsa-miR-451 | direct comparsion difficult- different experiment design (controls) |
| Sondermeijer 2011 | platelets | CAD at young age patients (premature CAD) | 12 patients | 12 healthy controls | Illumina Human v2 MicroRNA Beadarrays | 1142 microRNAs | validation 1: 40 premature CAD patients, 40 healthy controls, RT-PCR of 7 miRNAs  validation 2: families with prevalence of premature CAD 27, CAD 40 healthy controls, RT-PCR of 2 miRNAs | 7 miRNAs taken for further validation out of 214 miRNAs differentially expressed:  miR340*, miR615-5p, miR545:9.1, miR451, miR454* miR624*, miR-1280 |  |
| Stather 2013 | peripheral blood cells | PAD | 5 patients | 6 healthy controls | Whole-genome miRNA  expression profiling TaqMan Array Human  MicroRNA A+B Cards Set v3.0 | 754 miRNAs | 10 patients, 10 controls  qRT-PCR of 12 microRNAs using miRNA individual assays | hsa-let-7e  hsa-miR-15b  hsa-miR-16  hsa-miR-20b  hsa-miR-25  hsa-miR-26b  hsa-miR-27b  hsa-miR-28-5p  hsa-miR-126  hsa-miR-195  hsa-miR-335  hsa-miR-363 | direct comparsion difficult- different samples count, |
| Taurino 2010 | blood | severe CAD | 12 patients (10 of which after postcardiac rehabilitation) | 12 healthy controls | Illumina Beadstation platform, using Illumina Sentrix human ref-6 beadchips | 887 microRNAs | 7 predicted miRNA targeted genes confirmation by RT-PCR:  COX7C  UQCRQ  NDUFB3  NDUFA1  ATP5L  CASP3  ATP5I | CAD vs CAD postrehabilitation vs control:  miR-140-3p  miR-182  miR-92a  miR-92b,  20 mRNA genes as markers of mitochondrial function  ATP5C1  ATP5I  COX7B  COX7C  NDUFA4  NDUFB3  UQCRQ  ATP5J  ATP5L  ATP5O  ATP6V1D  IHPK2  NDUFA1  NDUFB2  NDUFB6  NDUFS5  APH1A  CASP3  CASP8  CAT |  |
| Vegter 2017 | plasma | atherosclerotic disease manifestation patients: CAD, PAD, stroke/TIA | 114 patients | 10 healthy controls from other study  (Wong et al 2011) | customized Exiqon miRNA PCR panel | 11 microRNAs | patients vs controls | CAD vs control:  let-7i-5p  miR-16-5p  miR-18a-5p  miR-26b-5p  miR-27a-3p  ***miR-30e-5p ↓***  miR-106a-5p  miR-199a-3p  miR-223-3p  ***miR-423-5p ↑***  miR-652-3p | direct comparsion difficult- different experiment design (controls), different count of microRNA analyzed |
| Weber 2011 | blood | CAD | patients  (**5 microarray** + 15 PCR validation) | Healthy subjects  (**5 microarray** + 15 PCR validation) | Affymetrix GeneChip miRNA arrays. MicroRNA targets were biotinylated using an Asuragen developed direct labeling procedure.  Quality assessment of the samples: TaqMan assays | 1770 microRNAs | 11 microRNAs RT-PCR on 10 CAD patients and 15 healthy subjects. | miR-150, miR-584, miR-19a, miR-145, miR-155, miR-222, miR-378, miR-29a, ***miR-30e-5p ↓***, miR-342, miR-181d | direct comparsion difficult- different sample count |
| ACAS- Asymptomatic Carotid Artery Stenosis; ACS- Acute Coronary Syndrome; AMI- Acute Myocardial Infraction; ASO- Atherosclerosis Obliterans; CAD- Carotid Artery Disease; LEAD- Low Artery Disease; PAD Peripheral Artery Disease; PBMCs- Peripheral Blood Mononuclear Cells; SAP- stable angina pectoris; TIA- Transient Ischemic Attack; UAP- Unstable Angina Pectoris, ***microRNAs differentially expressed in reported experiments, confirmed in current study***, ↓downregulated, ↑ upregulated | | | | | | | | | |

**References for Supplementary Table 14:**

Al-Kafaji, G., Al-Mahroos, G., Abdulla Al-Muhtaresh, H., Sabry, M.A., Abdul Razzak, R., and Salem, A.H. (2010). Circulating endothelium-enriched microRNA-126 as a potential biomarker for coronary artery disease in type 2 diabetes mellitus patients. *Biomarkers.* 22, 268-278. doi: 10.1080/1354750X.2016.1204004

Cipollone, F., Felicioni, L., Sarzani, R., Ucchino, S., Spigonardo, F., Mandolini, C., et al. (2011). A unique microRNA signature associated with plaque instability in humans. *Stroke*. 42, 2556-2563. doi: 10.1161/STROKEAHA.110.597575

D'Alessandra, Y., Carena, M. C., Spazzafumo, L., Martinelli, F., Bassetti, B., Devanna, P., et al. (2013). Diagnostic potential of plasmatic MicroRNA signatures in stable and unstable angina. PloS one, 8(11), e80345. doi:10.1371/journal.pone.0080345

Dolz, S., Górriz, D., Tembl, J.I., Sánchez, D., Fortea, G., Parkhutik, V., et al. (2017). Circulating MicroRNAs as Novel Biomarkers of Stenosis Progression in Asymptomatic Carotid Stenosis. *Stroke.* 48, 10-16. doi: 10.1161/STROKEAHA.116.013650

Dong, J., Liang, Y.Z., Zhang, J., Wu, L.J., Wang, S., Hua, Q., et al. (2017). Potential Role of Lipometabolism-Related MicroRNAs in Peripheral Blood Mononuclear Cells as Biomarkers for Coronary Artery Disease. *J Atheroscler Thromb*. 24, 430-441. doi: 10.5551/jat.35923

Fichtlscherer, S., De Rosa, S., Fox, H., Schwietz, T., Fischer, A., Liebetrau, C., et al. (2010). Circulating microRNAs in patients with coronary artery disease. *Circ Res*. 107, 677-684. doi: 10.1161/CIRCRESAHA.109.215566

Han, H., Qu, G., Han, C., Wang, Y., Sun, T., Li, F., et al. (2015). MiR-34a, miR-21 and miR-23a as potential biomarkers for coronary artery disease: a pilot microarray study and confirmation in a 32 patient cohort. *Exp Mol Med*. 47, e138. doi: 10.1038/emm.2014.8

Hoekstra, M., van der Lans, C.A., Halvorsen, B., Gullestad, L., Kuiper, J., Aukrust, P., et al. (2010). The peripheral blood mononuclear cell microRNA signature of coronary artery disease. *Biochem Biophys Res Commun.* 394, 792-797. doi: 10.1016/j.bbrc.2010.03.075

Huang, S., Chen, M., Li, L., He, M., Hu, D., Zhang, X., et al. (2014). Circulating MicroRNAs and the occurrence of acute myocardial infarction in Chinese populations. *Circ Cardiovasc Genet.* 7, 189-198. doi: 10.1161/CIRCGENETICS.113.000294

Jiang, Y., Wang, H.Y., Li, Y., Guo, S.H., Zhang, L., and Cai, J.H. (2014). Peripheral blood miRNAs as a biomarker for chronic cardiovascular diseases. *Sci Rep.* 4, 5026. doi: 10.1038/srep05026

Karakas, M., Schulte, C., Appelbaum, S., Ojeda, F., Lackner, K.J., Münzel, T., et al. (2017). Circulating microRNAs strongly predict cardiovascular death in patients with coronary artery disease-results from the large AtheroGene study. *Eur Heart J.* 38, 516-523. doi: 10.1093/eurheartj/ehw250

Leistner, D.M., Boeckel, J.N., Reis, S.M., Thome, C.E., De Rosa, R., Keller, T., et al. (2016). Transcoronary gradients of vascular miRNAs and coronary atherosclerotic plaque characteristics. *Eur Heart J.* 37, 1738-1749. doi: 10.1093/eurheartj/ehw047

Li, T., Cao, H., Zhuang, J., Wan, J., Guan, M., Yu, B., et al. (2011). Identification of miR-130a, miR-27b and miR-210 as serum biomarkers for atherosclerosis obliterans. *Clin Chim Acta.* 412, 66-70. doi: 10.1016/j.cca.2010.09.029

Patino, W.D., Mian, O.Y., Kan, J.G., Matoba, S., Bartlett, L.D., Holbrook, B., et al. (2005). Circulating transcriptome reveals markers of atherosclerosis. *PNAS.* 102, 3423-3428. doi: 10.1073/pnas.0408032102

Raitoharju, E., Lyytikäinen, L.P., Levula, M., Oksala, N., Mennander, A., Tarkka, M., et al. (2011). miR-21, miR-210, miR-34a, and miR-146a/b are up-regulated in human atherosclerotic plaques in the Tampere Vascular Study. *Atherosclerosis.* 219, 211-217. doi: 10.1016/j.atherosclerosis.2011.07.020

Ren, J., Zhang, J., Xu, N., Han, G., Geng, Q., Song, J., et al. (2013). Signature of circulating microRNAs as potential biomarkers in vulnerable coronary artery disease. *PLoS One*. 8, e80738. doi: 10.1371/journal.pone.0080738

Sondermeijer, B. M., Bakker, A., Halliani, A., de Ronde, M. W., Marquart, A. A., Tijsen, et al. (2011). Platelets in patients with premature coronary artery disease exhibit upregulation of miRNA340* and miRNA624*. PloS one, 6(10), e25946. doi:10.1371/journal.pone.0025946

Stather, P.W., Sylvius, N., Wild, J.B., Choke, E., Sayers, R.D., and Bown, M.J. (2013). Differential microRNA expression profiles in peripheral arterial disease. *Circ Cardiovasc Genet.* 6, 490-497. doi: 10.1161/CIRCGENETICS.111.000053

Taurino, C., Miller, W. H., McBride, M. W., McClure, J. D., Khanin, R., Moreno, M. U., et al. (2010). Gene expression profiling in whole blood of patients with coronary artery disease. Clinical science (London, England : 1979), 119(8), 335–343. doi:10.1042/CS20100043

Vegter, E.L., Ovchinnikova, E.S., van Veldhuisen, D.J., Jaarsma, T., Berezikov, E., van der Meer, P., et al. (2017). Low circulating microRNA levels in heart failure patients are associated with atherosclerotic disease and cardiovascular-related rehospitalizations. *Clin Res Cardiol*. 106, 598-609. doi: 10.1007/s00392-017-1096-z

Weber, M., Baker, M. B., Patel, R. S., Quyyumi, A. A., Bao, G., & Searles, C. D. (2011). MicroRNA Expression Profile in CAD Patients and the Impact of ACEI/ARB. Cardiology research and practice, 2011, 532915. doi:10.4061/2011/532915
